# A convenient and eco-friendly cerium(III) chloride-catalysed synthesis of methoxime derivatives of aromatic aldehydes and ketones

**DOI:** 10.1098/rsos.180279

**Published:** 2018-05-23

**Authors:** Iván Cortés, Teodoro S. Kaufman, Andrea B. J. Bracca

**Affiliations:** Instituto de Química Rosario (CONICET-UNR), Suipacha 531, S2002LRK, Rosario, SF, Argentina

**Keywords:** cerium(III) chloride-promoted reaction, methoximation, eco-friendly transformation

## Abstract

The use of CeCl_3_·7H_2_O as an efficient and eco-friendly promoter for the convenient synthesis of methoximes derived from aromatic aldehydes and ketones, is reported. The transformations entail the use of equimolar amounts of MeONH_2_·HCl and NaOAc in EtOH at 50°C, and no special precautions are needed with regard to the presence of oxygen. The scope and limitations of the transformation were studied and a reaction mechanism was proposed.

## 1. Introduction

**Figure 1.**
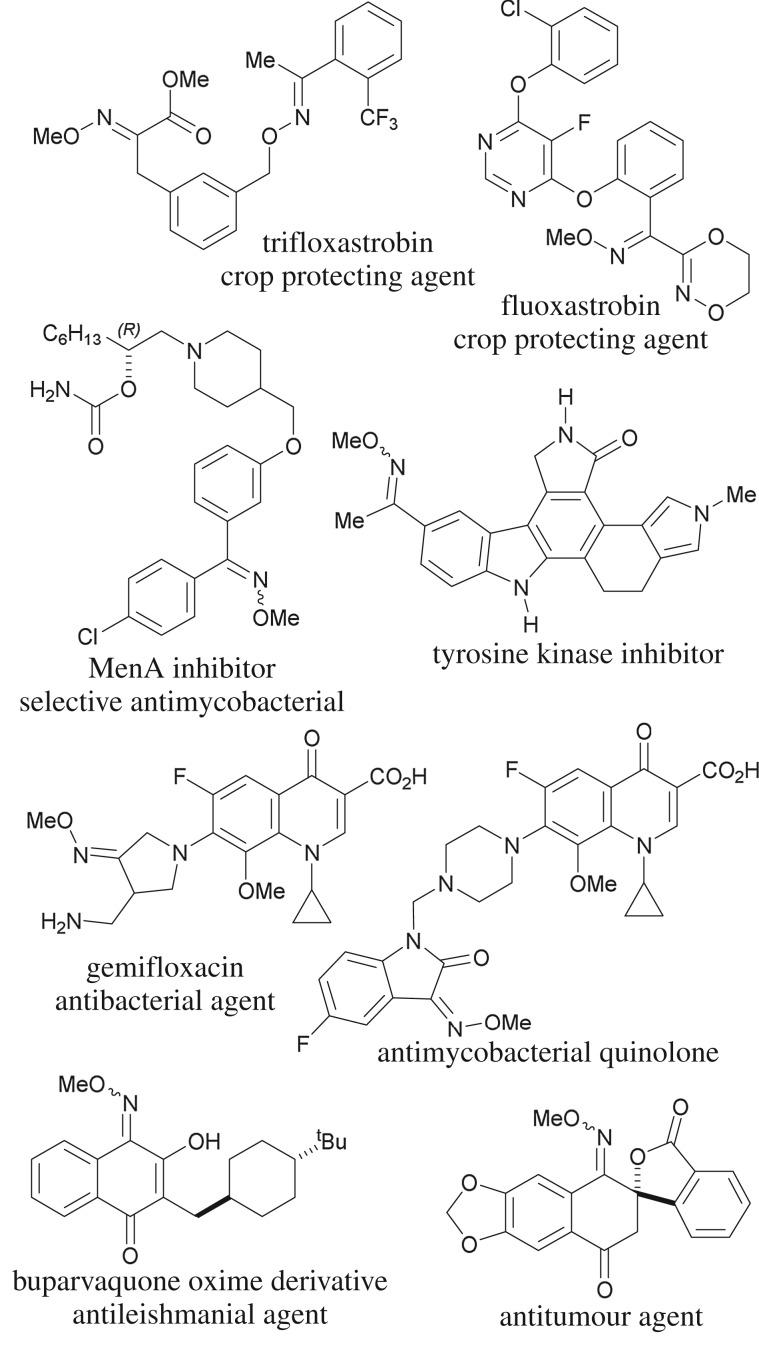
Selected relevant compounds displaying the methoxime motif.

The oxime ether moiety is an important structural motif found in relatively few natural products [1,2] and in a wide variety of pharmacologically relevant compounds. These include some anticonvulsants [3], antimycobacterials and antidepressants (fluvoxamine) [4], as well as antiparasitic (moxidectin) [5], antimicrobials (gemifloxacin, cefetamet) [6], antitumorals [7], enzyme inhibitors [8] (figure 1) and prodrugs, among others [9].

This functional group is also widespread among approved crop-protecting agents, as exemplified by the synthetic strobilurins trifloxastrobin and fluoxastrobin, for conferring them valuable selectivity in their biocidal properties [10].

Furthermore, oxime ethers are frequently present in the patent literature, being broadly used as intermediates in the chemical industry and in synthetic organic chemistry [11], especially for electrocyclization [12,13] and more recently for *ortho*-functionalization reactions of inactive ArC–H bonds [14,15], being also useful as aldehyde and ketone protecting groups [16] or as chromatographic derivatization agents [17]. In addition, oxime ethers have been employed as precursors of other functional groups, such as alkoxy-amides/lactams [18], nitriles [19] and amidines [20].

The methoximes are a special group among the oxime ethers. Conventional methods towards their synthesis [21] mostly rely on the reaction between methoxylamine and aromatic or aliphatic aldehydes or ketones [22]. (Hydro)alcoholic media, pyridine [22,23] and, occasionally, chlorinated compounds are employed as solvents [24]. Refluxing conditions are generally required for improved yields.

Some protocols include the use of molecular sieves, Na_2_SO_4_ or MgSO_4_ as water scavengers, to drive the reaction to a more rapid completion and/or to avoid heating [25,26]. Usual yields exceed 70%. The methoximation reagent is available as MeONH_2_·HCl, which is seldom employed alone; usually, a base (pyridine, Et_3_N, NaOAc, NaHCO_3_, Na_2_CO_3_ or K_2_CO_3_) is added [27,28], which enables the use of milder reaction conditions (less time, lower temperature).

Less conventional alternatives have also been reported, such as the *O*-methylation of oximes with different alkylating agents [4,29], the use of mineral acids (HCl) [30], stronger bases (NaOH) [31] and solid catalysts (Amberlyst A-21 [32] or silica gel [33]), under ultrasound [34], microwaves [35] and mechanochemical [36] promotion.

However, despite the significance of these protocols, most of them require a great excess of reagents [13], unnecessarily prolonged reaction times or heating under reflux [37], which may be incompatible with highly functionalized or sensitive compounds. Furthermore, although these disadvantages are known, the final attainment of reasonable yields after such unfavourable conditions has conspired against the development of milder procedures towards these widely used compounds.

We have repeatedly used methoximes as synthetic intermediates towards the elaboration of complex isoquinoline skeletons and have experienced the need of more efficient alternatives towards their preparation [38–40]. In this context we have observed the beneficial effects of the addition of CeCl_3_·7H_2_O [40].

Not long ago, it was considered that ‘catalytic processes … which take advantage of the unique properties of lanthanides are still rare' [41, p. 1922]. Therefore, herein, we wish to report the development of an expedient approach for the methoximation of aromatic aldehydes and ketones, under mild Lewis acid catalysis by CeCl_3_·7H_2_O. Despite the huge advances in catalysis which have taken place during the last two decades, to the best of our knowledge, promotion of the methoximation reaction has not yet been described with this handy compound or under any other lanthanide salt.

## 2. Material and methods

### 2.1. General information

The reactions were executed employing oven-dried glassware and freshly distilled anhydrous solvents. Absolute EtOH was obtained by refluxing the analytical reagent solvent with magnesium turnings and a crystal of iodine, followed by distillation from the so formed magnesium ethoxide. The other anhydrous solvents were dried according to the conventional procedures [42]. Anhydrous NaOAc was prepared by careful heating of the trihydrate and drying the so fused salt [42]. All other reagents were used as received.

The reactions were monitored by thin-layer chromatography (TLC). The chromatographic spots were detected by exposure to 254 nm ultraviolet light, and by spraying with ethanolic *p*-anisaldehyde/sulphuric acid reagent, followed by careful heating to improve selectivity. All new compounds gave single spots on TLC plates run in different solvent systems (hexanes or hexanes–EtOAc).

The flash column chromatographies were run with silica gel 60 H (particle size 63–200 µm), eluting with hexane–EtOAc mixtures, under positive pressure and employing gradient of solvent polarity techniques.

### 2.2. Apparatus

The melting points were measured on an Ernst Leitz Wetzlar model 350 hot-stage microscope. The Fourier transform infrared spectroscopy (FT-IR) spectra were recorded on a Shimadzu Prestige 21 spectrophotometer, as solid dispersions in potassium bromide (KBr) disks or as thin films held between NaCl cells.

The nuclear magnetic resonance (NMR) spectra were acquired in CDCl_3_ unless otherwise noted, on a Fourier transform nuclear magnetic resonance (FT-NMR) Bruker Avance 300 spectrometer, at 300.13 (^1^H) and 75.48 (^13^C) MHz. The chemical shifts are consigned in parts per million in the *δ* scale. Tetramethylsilane was used as the internal standard (resonances of CHCl_3_ in CDCl_3_: *δ* 7.26 and 77.0 for ^1^H and ^13^C NMR, respectively). An asterisk (*) designates signals which attribution can be exchanged. The coupling constants (*J*) and half-width (*w*_1/2_) values are given in Hertz. Some 2D-NMR experiments (COSY, HSQC) were also performed.

The high-resolution mass spectra were obtained from UMYMFOR (Buenos Aires, Argentina) and ICYTAC (Córdoba, Argentina) with Bruker MicroTOF-Q II instruments. Detection of the ions was performed in electrospray ionization, positive ion mode. The gas chromatography-mass spectrum (GC-MS) runs were carried out in a Shimadzu QP2010 Plus GC-MS instrument. Fragments are described with regards to their *m/z* ratios, in terms of relative intensity (%) of their signals. The specific optical rotations were measured at room temperature, with a Jasco DIP-1000 polarimeter using a microcell (1 cm path length).

### 2.3. General procedure for the syntheses of the methoximes **2a**–**v**

A stirred mixture of the aldehyde or ketone (**1**, 0.30 mmol), MeONH_2_·HCl (37.5 mg, 0.45 mmol, 1.5 equiv.) and anhydrous NaOAc (37 mg, 0.45 mmol, 1.5 equiv.) in absolute EtOH (2.5 ml) was treated with CeCl_3_·7H_2_O (5.6 mg, 5 mol%), and the reaction was heated to 50°C in a test tube, without special protection against atmospheric oxygen. The progress of the reaction was monitored by TLC. After completion, brine (10 ml) was added and the products were extracted with EtOAc (3 × 10 ml). The organic layers were combined, dried over MgSO_4_ and concentrated under reduced pressure. The residue was purified by column chromatography, eluting with mixtures of hexanes and EtOAc.

#### 2.3.1. (1*e*)-*N*-Methoxy-1-phenylethanimine (**2a**) [28]

Colourless oil; yield: 81%. IR (film, υ): 3000, 1647, 1604, 1529, 1497, 1480, 1385, 1325, 1204, 1100, 1085, 1000, 900, 785, 770 and 700 cm^−1^. ^1^H NMR: 2.23 (s, 3H, Me), 4.00 (s, 3H, OMe), 7.33–7.40 (m, 3H, H-3′, H-4′ and H-5′) and 7.60–7.68 (m, 2H, H-2′ and H-6′). ^13^C NMR: 12.6 (Me), 61.9 (N-OMe), 126.0 (C-2′ and C-6′), 128.4 (C-3′ and C-5′), 129.0 (C-4′), 136.7 (C-1′) and 154.7 (C-1). Electron ionization-mass spectrum (EI-MS) (*m/z*, %): 149 (M^+^, 59), 134 [(M-15)^+^, 5], 118 (38), 108 (7) and 77 (100).

#### 2.3.2. (1*e*)-1-(4-Bromophenyl)-*N*-methoxyethanimine (**2b**) [28]

Colourless oil; yield: 80%. IR (film, υ): 2936, 1609, 1587, 1485,1395, 1317, 1084, 1049, 1009, 895 and 824 cm^−1^. ^1^H NMR: 2.19 (s, 3H, Me), 3.99 (s, 3H, OMe) and 7.46–7.55 (m, 4H). ^13^C NMR: 12.4 (Me), 62.0 (N-OMe), 123.3 (C-4′), 127.6 (C-2′ and C-6′), 135.5 (C-3′ and C-5′) and 153.5 (C-1). EI-MS (*m/z*, %): 227 (M^+^, 100), 212 [(M-15)^+^, 1], 155 (68), 77 (35), 76 (54) and 75 (61).

#### 2.3.3. (1*e*)-*N*-Methoxy-1-(2-methoxyphenyl)ethanimine (**2c**) [24]

Colourless oil; yield: 79%. IR (film, υ): 2999, 2959, 2938, 2899, 2837, 2816, 1601, 1580, 1493, 1454, 1435, 1366, 1319, 1296, 1273, 1240, 1182, 1125, 1078, 1043, 1028, 883, 808, 756 and 632 cm^−1^. ^1^H NMR: 2.18 (s, 3H, Me), 3.82 (s, 3H, ArOMe), 3.82 (s, 3H, N-OMe), 6.90 (d, *J* = 8.3, 1H, H-3′), 6.95 (dt, *J* = 1.0 and 7.4, 1H, H-5′) and 7.27–7.36 (m, 2H, H-4′ and H-6′). ^13^C NMR: 16.0 (Me), 55.4 (ArOMe), 61.7 (N-OMe), 111.0 (C-3′), 120.6 (C-6′), 127.0 (C-5′), 129.5 (C-6′), 130.1 (C-4′) and 156.6 (2C, C-1 and C-2′). EI-MS (*m/z*, %): 179 (M^+^, 11), 133 (100), 105 (95) and 77 (75).

#### 2.3.4. (1*e*)-1-(3,4-Dimethoxyphenyl)-*N*-methoxyethanimine (**2d**) [21]

White solid; m.p.: 56–58°C; yield: 84%. IR (KBr, υ): 2961, 1576, 1516, 1464, 1416, 1339, 1277, 1252, 1231, 1175, 1150, 1045, 1020, 918, 866 and 812 cm^−1^. ^1^H NMR: 2.19 (s, 3H, Me), 3.88 (s, 3H, OMe-3),* 3.91 (s, 3H, OMe-4),* 3.97 (s, 3H, N-OMe), 6.83 (d, *J* = 8.4, 1H, H-5′), 7.13 (dd, *J* = 2.0 and 8.4, 1H, H-6′) and 7.29 (d, *J* = 2.0, 1H, H-2′). ^13^C NMR: 12.5 (Me), 55.9 (2C, 2 × OMe), 61.8 (N-OMe), 108.6 (C-2′), 110.6 (C-5′), 119.1 (C-6′), 129.4 (C-1′), 149.8 (C-3′),* 150.0 (C-4′)* and 154.2 (C-1). EI-MS (*m/z*, %): 209 (M^+^, 100), 194 [(M-15)^+^, 6], 178 (59) and 137 (41).

#### 2.3.5. 3-[(1*E*)-*N*-Methoxyethanimidoyl]aniline (**2e**) [43]

Colourless oil; yield: 86%. IR (film, υ): 3456, 3441, 3362, 2961, 2936, 1620, 1614, 1580, 1493, 1447, 1368, 1333, 1248, 1049, 920, 870, 785, 692 and 602 cm^−1^. ^1^H NMR: 2.18 (s, 3H, Me), 3.63 (s, 2H, NH_2_), 3.98 (s, 3H, N-OMe), 6.68 (ddd, *J* = 0.9, 2.3 and 7.8, 1H, H-4′), 6.97–7.02 (m, 1H, H-2′ and H-6′) and 7.15 (t, *J* = 8.0, 1H, H-5′). ^13^C NMR: 12.8 (Me), 61.8 (N-OMe), 112.4 (C-2′), 116.0 (C-4′), 116.6 (C-6′), 129.3 (C-5′), 137.7 (C-1′), 146.4 (C-3′) and 155.0 (C-1). EI-MS (*m/z*, %): 164 (M^+^, 24), 149 [(M-15)^+^, 4], 133 (20), 118 (18), 108 (21), and 92 (100).

#### 2.3.6. 4-[(1*E*)-*N*-Methoxyethanimidoyl]-2-nitrophenol (**2f**) [30]

White solid; m.p.: 89–92°C; yield: 84%. IR (KBr, υ): 3242, 2940, 1628, 1537, 1323, 1312, 1177, 1040, 883, 845, 764, 687 and 602 cm^−1^. ^1^H NMR: 2.21 (s, 3H, Me), 3.94 (s, 3H, N-OMe), 7.14 (d, *J* = 8.8, 1H, H-5′), 8.0 (dd, *J* = 2.3 and 8.8, 1H, H-6′), 8,30 (d, *J* = 2.3, 1H, H-2′) and 10.65 (s, 1H, OH). ^13^C NMR: 12.0 (Me), 62.2 (N-OMe), 120.1 (C-5′), 122.2 (C-2′), 129.4 (C-1′), 133.3 (C-6′), 134.9 (C-3′), 151.7 (C-1) and 155.5 (C-4′). EI-MS (*m/z*, %): 210 (M^+^, 88), 179 (73) and 133 (100).

#### 2.3.7. (1*e*)-1-[3,5-*bis*(Benzyloxy)phenyl]-*N*-methoxyethanimine (**2g**)

White solid; m.p.: 89–90°C; yield: 80%. IR (KBr, υ): 2930, 1603, 1580, 1435, 1377, 1360, 1169, 1061, 1043, 1022, 866, 839, 756, 742 and 700 cm^−1^. ^1^H NMR: 2.29 (s, 3H, Me), 4.02 (s, 3H, N-OMe), 5.06 (s, 4H, H-1a and H-1b), 6.64 (t, *J* = 2.2, 1H, H-4′), 6.94 (d, *J *= 2.2, 2H, H-2′ and H-6′) and 7.30–7.49 (m, 10H, Ar-H′ and Ar-H′′). ^13^C NMR: 12.7 (Me), 62.0 (N-OMe), 70.4 (C-1a and C-1b), 102.8 (C-2′ and C-6′), 105.5 (C-4′), 127.7 (4C, C-2′′, C-6′′, C-2′′′ and C-6′′′), 128.1 (2C, C-4′′ and C-4′′′), 128.6 (4C, C-3′′, C-5′′, C-3′′′ and C-5′′′), 136.8 (2C, C-1′′ and C-1′′′), 138.7 (C-1′), 154.4 (C-1) and 160.0 (2C, C-3′ and C-5′). High resolution mass spectrum (HRMS) (ESI-TOF, *m/z*): obsd. 384.1569; C_23_H_2_NNaO_3_ [(M + Na)^+^] requires 384.1576.

#### 2.3.8. (1*E*)-*N*-Methoxy-1-(2-nitrophenyl)ethanimine (**2h**) [44]

Yellowish oil; yield: 98%. IR (film, υ): 2938, 2820, 1611, 1537, 1346, 1047, 893, 787 and 630 cm^−1^. ^1^H NMR: 2.16 (s, 3H, Me), 3.96 (s, 3H, N-OMe), 7.45 (dd, *J* = 1.5 and 7.5, 1H, H-6′), 7.51 (td, 1H, H-4′), 7.63 (td, *J* = 1.3 and 7.5, 1H, H-5′) and 8.00 (dd, *J* = 1.3 and 8.1, 1H, H-3′). ^13^C NMR: 16.0 (Me), 62.1 (N-OMe), 124.6 (C-3′), 129.5 (C-4′), 130.6 (C-6′), 133.2 (C-1′), 133.3 (C-5′), 148.0 (C-2′) and 154.7 (C-1). EI-MS (*m/z*, %): 194 (M^+^, 6), 179 [(M-15)^+^, 1], 104 (52), 91 (73) and 77 (100).

#### 2.3.9. 3,5-Dimethoxy-2-[(1*E*)-*N*-methoxyethanimidoyl]phenol (**2i**)

Colourless oil; yield: 87%. IR (film, υ): 2938, 2841, 1643, 1454, 1368, 1215, 1155, 1111, 1051, 905 and 818 cm^−1^. ^1^H NMR: 2.26 (s, 3H, Ar-Me), 3.77 (s, 3H, OMe-5),* 3.79 (s, 3H, OMe-3),* 3.95 (s, 3H, N-OMe), 6.02 (d, *J* = 2.4, 1H, H-4′), 6.14 (d, *J* = 2.4, 1H, H-6′) and 10.66 (s, 1H, OH). ^13^C NMR: 16.1 (Me), 55.3 (OMe-5),* 55.5 (OMe-3),* 62.0 (N-OMe), 91.4 (C-4′), 93.6 (C-6′), 103.6 (C-2′), 158.2 (C-1), 158.4 (C-1′), 160.2 (C-3′) and 161.7 (C-4′). EI-MS (*m/z*, %): 225 (M^+^, 79), 194 (33), 179 (100) and 150 (30). HRMS (ESI-TOF, *m/z*): obsd. 226.1077; C_11_H_16_NO_4_ [(M + H)^+^] requires 226.1079.

#### 2.3.10. 4-[(1*E*)-*N*-Methoxypropanimidoyl]benzene-1,3-diol (**2j**)

Yellowish oil; yield: 89%. IR (film, υ): 3379, 2978, 2938, 1703, 1634, 1614, 1520, 1454, 1250, 1047, 970, 891, 851 and 743 cm^−1^. ^1^H NMR: 1.17 (t, *J* = 7.6, 3H, H-3), 2.77 (q, *J* = 7.6, 2H, H-2), 3.96 (s, 3H, N-OMe), 5.67 (s, 1H), 6.41 (dd, *J* = 2.6 and 8.6, 1H, H-4′), 6.46 (d, *J* = 2.5, 1H, H-2′), 7.27 (d, *J* = 8.5, 1H, H-5′) and 11.73 (s, 1H). ^13^C NMR: 11.4 (C-3), 18.8 (Me), 62.3 (N-OMe), 103.9 (C-2′), 107.1 (C-4′), 110.7 (C-6′), 128.8 (C-5′), 157.9 (C-3′), 160.0 (C-1′) and 163.2 (C-1). EI-MS (*m/z*, %): 195 (M^+^, 83), 180 [(M-15)^+^, 1], 164 (33), 135 (100) and 108 (57). HRMS (ESI-TOF, *m/z*): obsd. 196.0970; C_10_H_14_NO_3_ [(M + H)^+^] requires 196.0974.

#### 2.3.11. (1*e*)-3-Chloro-*N*-methoxy-1-phenylpropan-1-imine (**2k**) [45]

Colourless oil; yield: 96%. IR (film, υ): 2938, 2818, 1495, 1445, 1342, 1186, 1049, 899, 694 and 610 cm^−1^. ^1^H NMR: 3.23 (t, *J* = 7.7, 2H, H-2), 3.71 (t, *J* = 7.7, 2H, H-3), 4.01 (s, 3H, N-OMe), 7.36–7.44 (m, 3H, H-3′, H-4′ and H-5′) and 7.61–7.69 (m, 2H, H-2′ and H-6′). ^13^C NMR: 30.6 (C-2), 40.2 (C-3), 62.2 (N-OMe), 126.3 (2C, C-2′ and C-3′), 128.6 (2C, C-3′ and C-5′), 129.4 (C-4′), 135.1 (C-1′) and 154.6 (C-1). EI-MS (*m/z*, %): 197 (M^+^, 11), 162 (66), 130 (35), 104 (54) and 77 (100).

#### 2.3.12. *N*-Methoxy-1,1-diphenylmethanimine (**2l**) [46]

Colourless oil; yield: 94%. IR (film, υ): 3059, 2936, 2816, 1589, 1494, 1445, 1325, 1165, 1053, 1030, 982, 878, 772 and 696 cm^−1^. ^1^H NMR: 4.00 (s, 3H, N-OMe) and 7.30–7.53 (m, 10H, Ar-H and Ar-H′). ^13^C NMR: 62.5 (N-OMe), 127.9 (2C, C-3′ and C-5′), 128.2 (2C, C-3 and C-5), 128.3 (2C, C-2 and C-4), 128.9 (C-4′), 129.3 (2C, C-2′ and C-6′), 129.3 (C-4), 133.4 (C-1′), 136.5 (C-1) and 156.8 (C-1). EI-MS (*m/z*, %): 211 (M^+^, 45), 180 (54) and 77 (100).

#### 2.3.13. (1*e*)-4-Methylbenzaldehyde *O*-methyl oxime (**2m**) [29,47]

Colourless oil; yield: 69%. IR (film, υ): 2990, 2959, 2936, 2899, 1614, 1512, 1462, 1209, 1179, 1055, 955, 918, 854, 814 and 772 cm^−1^. ^1^H NMR: 2.37 (s, 3H, Ar-Me), 3.97 (s, 3H, N-OMe), 7.17 (d, *J* = 8.0, 2H, H-3′ and H-5′), 7.48 (d, *J* = 8.0, 2H, H-2′ and H-6′) and 8.04 (s, 1H, H-1). ^13^C NMR: 21.4 (Ar-Me), 61.9 (OMe), 127.0 (C2′ and C-6′), 129.4 (C-1′, C-2′ and C-3′), 140.0 (C-4′) and 148.6 (C-1). EI-MS (*m/z*, %): 149 (M^+^, 73), 134 [(M-15)^+^, 1], 122 (20), 118 (19), 107 (7), 91 (100), 79 (12) and 77 (15).

#### 2.3.14. (1*e*)-2-Hydroxy-3-methoxybenzaldehyde *O*-methyl oxime (**2n**)

White solid; m.p.: 75–76°C (Lit.: 79–80°C) [48]; yield: 97%. IR (KBr, υ): 2930, 1607, 1464, 1429, 1406, 1356, 1254, 1225, 1049, 916, 777, 731, 689 and 611 cm^−1^. ^1^H NMR: 3.89 (s, 3H, OMe), 3.97 (s, 3H, N-OMe), 6.79 (dd, *J* = 1.9 and 7.7, 1H, H-4′), 6.84 (t, *J* = 7.7, 1H, H-5′), 6.90 (dd, *J* = 1.9 and 7.7, 1H, H-6′), 8.14 (s, 1H, H-1) and 9.86 (s, 1H, OH). ^13^C NMR: 56.2 (OMe), 62.5 (N-OMe), 113.4 (C-4′), 116.5 (C-1′), 119.4 (C-5′), 122.3 (C-6′), 147.1 (C-3′), 148.2 (C-2′) and 151.1 (C-1). EI-MS (*m/z*, %): 181 (M^+^, 100), 166 [(M-15)^+^, 2], 150 (21), 132 (30) and 108 (26).

#### 2.3.15. (1*e*)-2,3-Dimethoxybenzaldehyde *O*-methyl oxime (**2o**)

White solid; m.p.: 51–52°C (Lit.: 58–59°C) [24]; yield: 92%. IR (KBr, υ): 2936, 1574, 1477, 1464, 1449, 1431, 1342, 1269, 1221, 1045, 997, 918, 795, 758 and 741 cm^−1^. ^1^H NMR: 3.83* (s, 3H, OMe-2′), 3.86* (s, 3H, OMe-3′), 3.97 (s, 3H, N-OMe), 6.91 (dd, *J* = 1.3 and 8.0, 1H, H-4′), 7.04 (t, *J* = 8.0, 1H, H-5′), 7.40 (dd, *J* = 1.3 and 8.0, 1H, H-6′) and 8.40 (s, 1H, H-1). ^13^C NMR: 55.8 (OMe-3′), 61.5 (OMe-2′), 62.0 (N-OMe), 113.5 (C-4′), 117.8 (C-1′), 124.2 (C-5′), 126.1 (C-6′), 144.5 (C-2′), 148.0 (C-1) and 152.9 (C-3′). EI-MS (*m/z*, %): 195 (M^+^, 28), 164 (5) and 149 (100).

#### 2.3.16. 4-[(*E*)-(Methoxyimino)methyl]benzene-1,3-diol (**2p**)

White solid; m.p.: 116–118°C (Lit.: 117–118°C) [48]; yield: 97%. IR (KBr, υ): 3995, 3397, 1636, 1614, 1578, 1512, 1437, 1420, 1317, 1298, 1217, 1167, 1125, 1057, 962, 941, 812, 692 and 602 cm^−1^. ^1^H NMR: 3.94 (s, 3H, N-OMe), 5.68 (s, 1H, OH-1), 6.41 (dd, *J* = 2.4 and 8.3, 1H, H-5′), 6.46 (d, *J* = 2.4, 1H, H-3′), 7.00 (d, *J* = 8.3, 1H, H-6′), 8.08 (s, 1H, H-1), 10.13 (s, 1H, OH-3). ^13^C NMR: 62.4 (N-OMe), 103.4 (C-3′), 107.7 (C-5′), 110.0 (C-1′), 132.2 (C-6′), 151.1 (C-1), 158.4 (C-4′), 159.1 (C-2′). EI-MS (*m/z*, %): 167 (M^+^,100), 135 (29), 109 (21), 108 (82) and 94 (34).

#### 2.3.17. (*E*)-2-Chlorobenzaldehyde *O*-methyl oxime (**2q**) [24]

Colourless oil; yield: 92%. IR (film, υ): 2936, 1601, 1472, 1433, 1342, 1209, 1063, 1045, 924, 851, 754, 704, 619 and 602 cm^−1^. ^1^H NMR: 3.99 (s, 3H, N-OMe), 7.21–7.32 (m, 2H, H-4′ and H-5′), 7.33–7.39 (m, 1H, H-3′), 7.88 (dd, *J* = 2.5 and 7.0, 1H, H-6′) and 8.48 (s, 1H, H-1). ^13^C NMR: 62.2 (N-OMe), 126.9 (C-3′), 127.1 (C-6′), 129.8 (C-4′), 130.0 (C-1′), 130.7 (C-5′), 133.8 (C-2′) and 145.6 (C-1). EI-MS (*m/z*, %): 169 (M^+^, 95), 138 (31), 134 (58), 111 (73), 102 (100) and 75 (80).

#### 2.3.18. (*E*)-2-(Trifluoromethyl)benzaldehyde *O*-methyl oxime (**2r**)

Colourless oil; yield: 88%. IR (film, υ): 2968, 2941, 2903, 2822, 1487, 1454, 1360, 1315, 1283, 1171, 1125, 1067, 1049, 1034, 962, 932, 854, 768, 750, 662 and 629 cm^−1^. ^1^H NMR: 4.02 (s, 3H, OMe), 7.46* (t, *J* = 7.5, 1H, H-4′), 7.54* (t, *J* = 7.5, 1H, H-5′), 7.67 (d, *J* = 7.8, 1H, H-3′), 8.06 (d, *J* = 7.8, 1H, H-6′) and 8.43 (q, *J* = 2.1, 1H, H-1). ^13^C NMR: 62.3 (N-OMe), 125.8 (q, *J* = 5.5, C-3′), 127.2 (C-6′), 128.0 (CF_3_), 128.4 (C-2′), 129.4 (C-4′), 130.4 (C-1′), 131.9 (C-5′) and 145.2 (C-1). EI-MS (*m/z*, %): 203 (M^+^,89), 152 (77), 145 (100), 125 (26) and 75 (51). HRMS (ESI-TOF, *m/z*): obsd. 204.0608; C_9_H_9_F_3_NO [(M + H)^+^] requires 204.0611.

#### 2.3.19. (*E*)-1-(3,4-Dimethoxyphenyl)propan-2-one *O*-methyl oxime (**2s**) [49]

Colourless oil; *E/Z* diastereomeric mixture (*E/Z*: 2.8:1); yield: 96%. IR (KBr, υ): 2997, 2938, 2907, 2835, 2816, 1607, 1591, 1514, 1504, 1454, 1445, 1418, 1263, 1236, 1153, 1140, 1055, 1030, 881, 806, 766 and 664 cm^−1^. *E*-isomer: ^1^H NMR: 1.73 (s, 3H, Me-3), 3.39 (s, 2H, H-1), 3.85 (s, 3H, OMe-4′) 3.86 (s, 3H, OMe-3′), 3.88 (s, 3H, N-OMe) and 6.69–6.82 (m, 3H, H-2′, H-5′ and H6′). ^13^C NMR: 13.5 (Me), 41.7 (C-2), 55.9 (OMe-2′ and OMe-4′), 61.3 (N-OMe), 111.2 (C-5′), 112.0 (C-2′), 121.0 (C-6′), 129.4 (C-1′), 147.9 (C-4′), 149.0 (C-3′) and 156.8 (C-2). *Z*-isomer: 1.77 (s, 3H, Me), 3.60 (s, 2H, H-1), 3.85 (s, 3H, OMe-4′), 3.86 (s, 3H, OMe-3′), 3.88 (s, 3H, N-OMe) and 6.69–6.82 (m, 3H, H-2′, H-5′ and H-6′). ^13^C NMR: 19.6 (Me-3), 34.9 (C-2), 55.9 (2C, OMe-2′ and OMe-4′), 61.3 (N-OMe), 111.2 (C-5′), 112.0 (C-2′), 121.0 (C-6′), 129.4 (C-1′), 147.9 (C-4′), 149.0 (C-3′) and 156.8 (C-2). EI-MS (*m/z*, %): 223 (M^+^,82), 176 (51), 151 (100), 135 (21) and 105 (19).

#### 2.3.20. Methoxy({[(4*S*)-4-(prop-1-en-2-yl)cyclohex-1-en-1-yl]methylidene})amine[*S*-perillaldehyde(*E*)-*O*-methyl oxime] (**2t**) [50]

Colourless oil; yield: 79% (greater than 98% *E*). [*α*]_D_^16^ = –134.4 (*c*, 0.84, CHCl_3_). IR (film, υ): 2936, 2899, 1643, 1454, 1435, 1373, 1179, 1059, 1045, 947, 897 and 667 cm**^−^**^1^. ^1^H NMR: 1.47 (dddd, 1H, *J* = 5.4, 11.2, 12.4 and 12.8, H-5_ax_), 1.74 (s, 3H, H-9), 1.83−1.93 (m, 1H, H-5_eq_), 2.01−2.09 (m, 1H, H-4), 2.11−2.29 (m, 3H, H-3_ax_ and H-6), 2.47 (ddd, 1H, *J* = 2.7, 2.3 and 17.3, H-3_eq_), 3.85 (s, 3H, N-OMe), 4.72 (bs, 1H, H-8a), 4.74 (bs, 1H, H-8b), 5.98 (bdd, 1H, *J* = 2.4 and 5.2, H-2) and 7.65 (s, 1H, H-10). ^13^C NMR: 20.7 (H-9), 23.9 (H-5), 26.8 (H-6), 31.3 (H-3), 40.9 (H-4), 61.6 (N-OMe), 109.0 (H-8), 132.6, 134.9, 149.2 and 151.6. EI-MS (*m/z*, %): 179 (M^+^, 2), 164 [(M-15)^+^, 2], 138 (4), 110 (50), 80 (100) and 68 (44).

#### 2.3.21. (*R,E*)-2-Methyl-5-(prop-1-en-2-yl)cyclohex-2-enone *O*-methyl oxime [*R*-carvone (*E*)-*O*-methyl oxime] (**2u**) [51]

Colourless oil; yield: 82% (greater than 98% *E*). [*α*]_D_^16^ = –11.0 (*c*, 0.15, CHCl_3_). IR (film, υ): 2965, 2957, 2936, 2924, 2899, 2855, 2837, 2816, 1645, 1441, 1375, 1123, 1051, 908, 891 and 799 cm**^−^**^1^. ^1^H NMR *δ*: 1.74 (s, 3H, H-10), 1.79 (bs, 3H, *w*_1/2_ = 5.0, H-9), 2.01 (dd, 1H, J = 12.6 and 16.4, H-6_ax_), 2.03**–**2.13 (m, 1H, H-4_ax_), 2.18**–**2.38 (m, 2H, H-4_eq_, and H-5), 3.13 (ddd, 1H, *J* = 1.6, 3.9 and 16.5, H-6_eq_), 3.90 (s, 3H, N-OMe), 4.79 (m, 2H, *w*_1/2_ = 8.7, H-8), and 5.91 (m, 1H, *w*_1/2_ = 10.9, H-3). ^13^C NMR *δ*: 17.6 (C-10), 20.7 (C-9), 27.8 (C-6), 30.3 (C-4), 40.4 (C-5), 61.7 (N-OMe), 109.8 (C-8), 130.5 (C-2), 132.3 (C-3), 148.0 (C-7) and 156.1 (C-1). EI-MS (*m/z*, %): 179 (M^+^, 20), 164 [(M-15)^+^, 4], 148 (14), 138 (62), 107 (100), 105 (89), 91 (70) and 80 (51).

#### 2.3.22. (2*r*,5*R*,*E*)-2-Methyl-5-(prop-1-en-2-yl)cyclohexanone *O*-methyl oxime [1,2-dihydrocarvone (*E*)-*O*-methyl oxime] (**2v**) [51]

Colourless oil; yield: 96% (greater than 95% *E*). [*α*]_D_^16^ = –86.0 (*c*, 0.74, CHCl_3_); IR (film, υ): 2963, 2930, 2855, 1643, 1447, 1378, 1261, 1051, 905, 880 and 849 cm^−1^; ^1^H NMR: 1.10 (d, *J* = 6.8, 3H, H-10), 1.25 (ddd, 1H, *J* = 3.2, 12.7 and 24.2, H-4_ax_), 1.41 (ddd, 1H, *J* = 3.2, 12.5 and 24.2, H-3_ax_), 1.58 (t, 1H, J = 13.2, H-6_eq_), 1.74 (s, 3H, H-9), 1.80–1.90 (m, 1H, H-3_eq_), 1.90–2.00 (m, 1H, H-4_eq_), 2.00–2.11 (ddt, 1H, *J* = 4.0, 12.2 and 13.2, H-5), 2.12–2.24 (m, 1H, H-2), 3.32 (ddd, 1H, *J* = 2.1, 4.0 and 13.2, H-6), 3.82 (s, 3H, N-OMe) and 4.73 (bs, 2H, *w*_1/2_= 3.0, H-8). ^13^C NMR: 16.4 (C-10), 20.8 (C-9), 29.8 (C-6), 30.9 (C-3), 35.4 (C-4), 37.2 (C-2), 44.8 (C-5), 61.1 (N-OMe), 109.2 (C-8), 148.7 (C-7) and 161.7 (C-1). EI-MS (*m/z*, %): 181 (M^+^, 2), 166 [(M-15)^+^, 2], 149 (2), 125 (8), 109 (13), 97 (50) and 71 (100).

#### 2.3.23. (1*e*)-*N*-Hydroxy-1-phenylethanimine (**2w**)

A stirred mixture of acetophenone (**1a**, 36** **mg, 0.30** **mmol), HONH_2_·HCl (37.5** **mg, 0.45** **mmol, 1.5 equiv.) and anhydrous NaOAc (37** **mg, 0.45** **mmol, 1.5 equiv.) in absolute EtOH (2.5 ml) was treated with CeCl_3_·7H_2_O (8.5** **mg, 5 mol%), and the reaction was heated to 50°C in a test tube, without special protection against atmospheric oxygen. The progress of the reaction was monitored by TLC. After 25** **min, brine (10 ml) was added and the products were extracted with EtOAc (3 × 10 ml). The organic layers were combined, dried over MgSO_4_ and concentrated under reduced pressure. The residue was purified by column chromatography (hexanes : EtOAc) affording **2w** (42.5** **mg, 95%), as a white solid, m.p.: 56–58°C (Lit.: 55–57°C) [52]. IR (KBr, υ): 3316, 2926, 1445, 1371, 1302, 1080, 1009, 928, 762 and 692 cm^−1^; ^1^H NMR: 2.32 (s, 3H, Me), 7.45–7.34 (m, 3H, H-3′, H-4′ and H-5′), 7.69–7.59 (m, 2H, H-2′ and H-6′) and 9.45 (s, 1H, OH); ^13^C NMR: 12.4 (Me), 126.0 (C-2′ and C-6′), 128.6 (C-3′ and C-5′), 129.3 (C-4′), 136.5 (C-1′) and 156.0 (C-2). EI-MS (*m/z*, %): 135 (M^+^, 61), 120 [(M-15)^+^, 3], 118 (12), 106 (20), 103 (19), 94 (27) and 77(100).

## 3. Results and discussion

At the outset of the study, based on previous experience [40] and on the literature regarding non-promoted methoximations, the development of an optimal protocol was sought, with acetophenone (**1a**) as the chosen model starting material, in EtOH as solvent. Trial and error experiments, which included heating from room temperature to reflux, revealed that incubating the reaction at 50°C provided the most convenient conversions in short times and under mild conditions.

On the other hand, progressive reduction of the excess of methoxylamine (range 2.5–1.1 equiv.) exposed that 1.5 equiv. of each, MeONH_2_·HCl and the base, were required for optimal results. Lowering the amount of MeONH_2_·HCl to 1.2 equiv. or below afforded either unfinished transformations or long reaction times. Hence, warming the reaction at 50°C in the presence of 1.5 equiv. each methoxylamine hydrochloride and the base were set as the initial conditions for further improvements.

Then, the qualitative and quantitative effects of the addition of catalytic amounts of different Lewis acids were explored, using Ba(II), Cd(II), Ce(III), Cu(I), Cu(II), Fe(II), Fe(III), La(III), Mn(II), Mn(III), Ni(II), Sc(III) and Zn(II), salts, at a level of 5 mol%.

The transformations were monitored by GC-MS, employing anisole as internal standard, with the results detailed in table 1. This enabled us to observe that, in the absence of a promoter, the reaction afforded only 21% yield of the expected methoxime **2a** after 40** **min (entry 1) and required 12** **h to reach completion. It was also evident that Ba(OAc)_2_·H_2_O was ineffective under these conditions (entry 2), with results analogous to the non-promoted process.

**Table 1.** Screening of Lewis acids as promoters for the methoximation of acetophenone (**1a**).^a^ 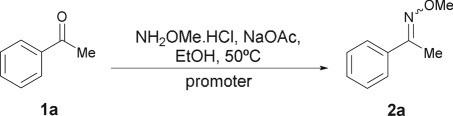

		yield (%)^b^		
entry no	promoter	20 min	30 min	40 min
1	—	10	17	21
2	Ba(OAc)_2_·H_2_O	12	16	20
3	CuI	20	26	30
4	Cu(OAc)_2_·H_2_O	18	25	31
5	La(NO_3_)_3_·6H_2_O	18	24	33
6	FeSO_4_·7H_2_O	27	33	42
7	FeCl_3_	25	37	43
8	Mn(OAc)_3_·2H_2_O	29	36	44
9	CdCl_2_·*x*H_2_O	31	39	47
10	Sc(TfO)_3_	34	51	65
11	ZnI_2_	53	79	100
12	MnSO_4_·4H_2_O	83	96	100
13	MnCl_2_·4H_2_O	80	100	100
14	CeCl_3_·7H_2_O	89	100	100
15	CeCl_3_ (anhydrous)	100	100	100

^a^Reaction conditions: acetophenone (0.3 mmol, 1.0 equiv.), MeOHNH_2_·HCl (1.5 equiv.), NaOAc (1.5 equiv.), promoter (5 mol%), absolute EtOH (2.5 ml), anisole (internal standard, 0.3 mmol), 50°C.

^b^Yields were determined by GC-MS analysis, employing anisole as internal standard.

Copper salts performed only slightly better; however, regardless of their different solubility and oxidation state, the outcome was similar. At 40 min CuI afforded only 30% yield of the product **2a** (entry 3), whereas Cu(OAc)_2_·H_2_O gave the methoxime in 31% yield (entry 4), both being found to be not viable to favour this transformation. Interestingly, an undisclosed copper salt supported on silica gel has been recently proposed as promoter for the preparation of oximes derived from aromatic aldehydes, in reactions taking 2–3 h to reach completion [53].

The use of La(NO_3_)_3_.6H_2_O gave similar results (entry 5), while the tested iron salts furnished approximately 10% increment in the yields (entries 6 and 7); an analogous behaviour was observed in the reactions promoted by Mn(OAc)_3_·2H_2_O and CdCl_2_·*x*H_2_O, which resulted in 44% and 47% of **2a**, respectively, at the 40 min check time (entries 8 and 9).

On the other hand, addition of Sc(OTf)_3_ caused a performance jump to 65% yield at the end of the standard 40 min period (entry 10), while the reactions run under promotion by ZnI_2_, MnSO_4_·4H_2_O MnCl_2_·4H_2_O and CeCl_3_·7H_2_O afforded quantitative yields of the methoxime **2a** at this time-point (entries 11–14).

Interestingly, despite the importance of the counterions in Lewis acid-mediated carbonyl activation on the outcome of the thus triggered transformations [54], the performances of the reactions promoted by MnSO_4_·4H_2_O and MnCl_2_·4H_2_O were similar.

Moreover, inspection of the reaction yields at earlier times, revealed that the behaviour of MnSO_4_·4H_2_O, MnCl_2_·4H_2_O and CeCl_3_·7H_2_O was quite similar and better than ZnI_2_, with CeCl_3_·7H_2_O slightly outperforming the manganous salts, when the reaction was checked at the 20 min mark (89% versus 80% for MnCl_2_·4H_2_O and 83% for MnSO_4_·4H_2_O).

Notably, the superior promotion effects observed for CeCl_3_·7H_2_O are fully consistent with those reported in other transformations involving carbonyl moieties [41,55,56]. Furthermore, anhydrous CeCl_3_ [57] performed even better than the hydrate at short reaction times (entry 15). However, because the eco-friendly CeCl_3_·7H_2_O is inexpensive, commercially available and easier to handle, it was selected for further optimization of the model reaction.

The nature of the reaction solvent was also optimized (table 2, entries 1–11), with the use of CeCl_3_·7H_2_O as promoter in the presence of NaOAc. It was observed that the transformation did not proceed at all in PhMe, CHCl_3_, MeCN and dioxane (entries 1–4), whereas it afforded a meagre 30% yield of **2a** in THF, after 40 min (entry 5).

**Table 2.** Optimization of the reaction conditions for the methoximation of acetophenone (**1a**).^a^ 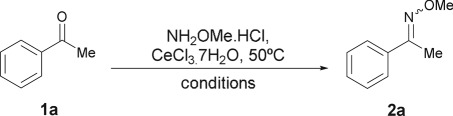

				yield (%)^b^		
entry no.	solvent	base (1.5 equiv.)	promoter load (mol%)	20 min	30 min	40 min
1	PhMe	NaOAc	5	0	0	0
2	CHCl_3_	NaOAc	5	0	0	0
3	MeCN	NaOAc	5	0	0	0
4	dioxane	NaOAc	5	0	0	0
5	THF	NaOAc	5	11	19	30
6	MeOH	NaOAc	5	77	84	91
7	*i*-PrOH	NaOAc	5	64	65	75
8	*t-*BuOH	NaOAc	5	81	82	89
9	EtOH	NaOAc	5	89	100	100
10	EtOH 96%	NaOAc	5	89	99	100
11	EtOH 80%	NaOAc	5	73	93	99
12	EtOH	Et_3_N	5	74	92	100
13	EtOH	NaHCO_3_	5	83	93	100
14	EtOH	K_2_HPO_4_	5	84	99	100
15	EtOH	K_2_CO_3_	5	73	84	90
16	EtOH	NaOAc	2	24	37	47
17	EtOH	NaOAc	4	74	92	100
18	EtOH	NaOAc	6	91	100	100
19	EtOH	NaOAc	8	93	100	100

^a^Reaction conditions: ketone **1a** (0.3 mmol, 1.0 equiv.), MeONH_2_·HCl (1.5 equiv.), solvent (2.5 ml), 50°C.

^b^Yields determined by GC-MS analysis, with anisole as internal standard.

Oppositely, alcoholic solvents (MeOH, EtOH, *^i^*PrOH, *^t^*BuOH) proved to be suitable media to achieve moderate-to-excellent yields of the product (64–89% after 20 min, entries 6–9). Probably, this is owing to the better solubility of all the reactants in alcoholic solvents and to their potential interaction with the promoter. Interestingly, it was shown that CeCl_3_ forms a dimeric adduct with MeOH, [Ce_2_Cl_6_(MeOH)_8_] that persists in solution and the corresponding ethanol adduct can also be prepared [58,59].

Hence, EtOH emerged from these experiments as the most advantageous solvent alternative (entry 9). It was also found that the reactions can also be carried out without special protection against oxygen. However, the absolute grade solvent proved to be more efficient than its mixtures (up to 20% v/v) with water (entries 9–11). In these cases, it was observed that the presence of H_2_O did not hinder the transformation, but it seemed to slightly lower the reaction rate.

On the other hand, the aptitude of mild bases (NaHCO_3_, K_2_CO_3_, K_2_HPO_4_ and Et_3_N) other than NaOAc, to free the methoxime base was also evaluated (entries 12–15) in EtOH. However, despite their excellent performances, especially in the case of K_2_HPO_4_ (99% at 30** **min and 100% at 40** **min, entry 14), none of them surpassed that of NaOAc.

In addition, when 0.15 M solutions of MeONH_2_·HCl in EtOH (2.5 ml) were treated with NaOAc (0.90, 1.0 and 1.1 equiv.) and diluted with water (5.0 ml), they exhibited essentially the same pH values (4.83, 4.89 and 4.91, respectively), confirming the robustness of the method. Under these conditions Ce(III) is stable in solution and it has been shown that oximation reactions are slow and their rate has a maximum between pH 4 and 5 [60]. Accordingly, NaOAc was selected as the added base for further experiments.

Finally, the load of the promoter was analysed, in the range 2–8 mol% (entries 9, 16–19), observing that the product yield was quantitative at the 30 min checkpoint with loads of at least 5 mol% (entries 9, 18 and 19). However, while it was found that the reaction was complete in 40 min at a 4 mol% promoter level, it was also concluded that loads above 5 mol% did not produce any substantial improvement at the 20 min control time. Therefore, the latter level was chosen as the optimum.

The resulting protocol (MeONH_2_·HCl and NaOAc (1.5 equiv. each) and 5 mol% CeCl_3_.7H_2_O in EtOH at 50°C) proved to be mild and respectful towards sensitive compounds, while remaining a highly discriminating condition against the non-catalysed process.

Next, the scope of the optimized methodology was explored, employing various aromatic ketones and aldehydes with different substituents and substitution patterns (table 3). In general, very good-to-excellent yields were obtained at 50°C with both, aromatic ketones (entries 1–12) and aldehydes (entries 13–18), usually taking place in short reaction times. Being more reactive, the best results were achieved with the latter ones, in which cases the transformations were also completed in comparatively shorter times.

**Table 3.** Scope of the CeCl_3_·7H_2_O-promoted methoximation reaction.^a^ 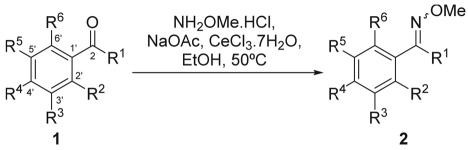

entry no.	R^1^	R^2^	R^3^	R^4^	R^5^	R^6^	time (h)	prod. no.	yield (%)^b^
1	Me	H	H	H	H	H	0.5	**2a**	100
2	Me	H	H	Br	H	H	0.67	**2b**	80
3	Me	OMe	H	H	H	H	1.0	**2c**	79
4	Me	H	OMe	OMe	H	H	1.0	**2d**	84
5	Me	H	NH_2_	H	H	H	0.4	**2e**	86
6	Me	H	NO_2_	OH	H	H	2.0	**2f**	84
7	Me	H	OBn	H	OBn	H	1.1	**2g**	80
8	Me	NO_2_	H	H	H	H	18	**2h**	98
9	Me	OH	H	OMe	H	OMe	18*^d^*	**2i**	87
10	CH_2_Me	OH	H	OH	H	H	18*^d^*	**2j**	89
11	CH_2_CH_2_Cl	H	H	H	H	H	0.67	**2k**	96
12	Ph	H	H	H	H	H	18	**2l**	94
13	H	H	H	Me	H	H	0.33	**2m**	90
14	H	OH	OMe	H	H	H	0.4	**2n**	97
15	H	OMe	OMe	H	H	H	0.33	**2o**	92
16	H	OH	H	OH	H	H	0.4	**2p**	97
17	H	Cl	H	H	H	H	0.67	**2q**	92
18	H	CF_3_	H	H	H	H	0.4	**2r**	88
19	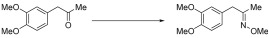						0.4	**2s**	96
20	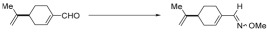						0.6	**2t**	79
21	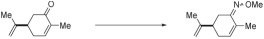						1.33	**2u**	82
22	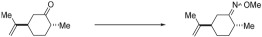						0.4	**2v**	96
23*^c^*	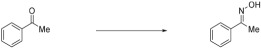						0.4	**2w**	95

^a^Reaction conditions: aldehyde or ketone (0.3 mmol, 1.0 equiv.), MeONH_2_·HCl (1.5 equiv.), CeCl_3_·7H_2_O (5 mol%), NaOAc (1.5 equiv.), EtOH (2.5 ml), 50°C.

^b^Isolated product yields after column chromatography.

*^c^*The reaction was performed with 1.5 equiv. HONH_2_·HCl.

*^d^*The reaction was terminated at the informed time. Starting material left.

Further analysis of the results revealed that no significant effects were detected owing to the presence of either electron withdrawing (entries 2, 6, 8, 17 and 18) or electron donating (entries 3–5, 7, 9 and 10) groups attached to different positions of the aromatic ring. However, the nitro derivatives of entries 6 and 8 reacted at a lower rate, taking longer times to reach completion.

The reaction conditions also proved to be compatible with *ortho*-substituents (entries 3, 8–10 and 14–18), without substantial loss of performance, except that compounds exhibiting bulky *ortho*-substituents (entries 8, 9 and 12) and ketones displaying *ortho* hydroxy groups (entries 9 and 10) were methoximated in good yields, at the expense of rather longer reaction periods.

In the case of 2-hydroxyketones, some starting material was recovered at the end of the reaction period. Presumably, this may be a result of the presence of a hydrogen bond between the phenol and the carbonyl moieties. The transformation was also viable in the presence of free phenols and free amines in different positions (entries 5, 6, 9, 10, 14 and 16) and took place with aromatic ketones other than acetophenones (entries 10–12).

On the other side, the performances of the reactions with 3,4-dimethoxyphenylacetone (entry 19) [61,62], perillaldehyde (entry 20) [63], carvone (entry 21) [64,65] and (+)-1,2-dihydrocarvone (entry 22) [65] confirmed that the transformation can also take place efficiently with aliphatic/alicyclic aldehydes and ketones, even in the presence of some steric hindrance and double bond conjugation.

The conjugate addition of hydroxylamine derivatives to *α*,β-unsaturated carbonyls is a serious side reaction in certain systems, leading sometimes to undesired products [49,50,66]. Lewis acids have been found to promote carbonyl activation, favouring this process [51,67,68]. Fortunately, however, no products arising from conjugate addition were observed in the experiments of entries 20 and 21, suggesting that the reaction conditions are mild enough to prevent this reaction, and that carbonyl methoximation is faster than the Michael addition. The methoxime product, being less reactive, is less likely to undergo a conjugate addition.

In addition, it was observed that the Ce(III)-promoted reaction was also successful with hydroxylamine. When acetophenone was used as substrate, 95% yield of the expected oxime **2w** was obtained after 25** **min (entry 23); in comparison, the non-catalysed process took over 140** **min to reach completion under the same conditions.

The structures of the different products were assessed by their melting points, as well as by IR and NMR (^1^H and ^13^C) spectroscopy, being all in full agreement with their proposed structures and with the corresponding literature data. Not unexpectedly, in many cases they were obtained as mixtures of *anti/syn* (*E/Z*) isomeric compounds that could not be separated chromatographically.

The major products were assigned as the *anti*-isomers on the basis of comparative analysis of their ^1^H NMR spectral data and the known tendency of acetophenone oximes to preferentially adopt the anti-configuration [69]. Furthermore, most of the methoximes are known compounds and were chosen with the purpose of comparing the performance of the proposed cerium(III)-promoted transformation with previous results (cf. electronic supplementary material).

In this analysis it was observed that, in general, the proposed alternative was advantageous and outperformed the previously reported methodologies, providing improved yields of the products under milder conditions, in shorter reaction times and/or at the expense of lower excess of reagents (MeONH_2_·HCl and base).

**Scheme 1.**
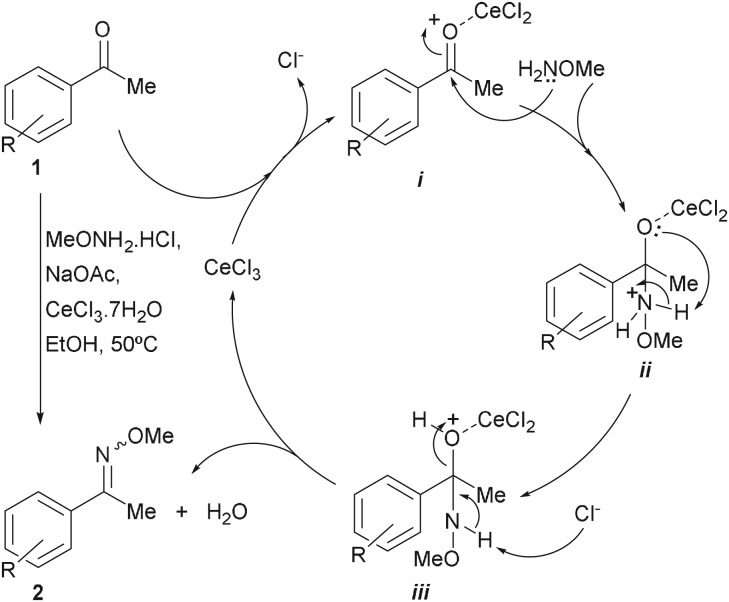
Proposed reaction mechanism for the catalytic cycle of the CeCl_3_·7H_2_O-promoted methoximation of aromatic aldehydes and ketones.

Although the exact mechanistic details of the reaction remain unknown, a catalytic cycle, like that depicted in scheme 1, can be drawn by analogy with similar transformations, such as other Lewis acid-mediated protection of ketones and aldehydes.

The reaction can be assumed to take place through a stepwise process. In the first stage, the oxophilic promoter coordinates with the carbonyl moiety of **1** to afford the activated intermediate ***i***. This intermediate is more likely than the starting carbonyl derivative to undergo nucleophilic attack by the nitrogen of the methoxylamine and furnish intermediate ***ii*** in a second step.

In the next phase, a proton transfer within this intermediate would generate intermediate ***iii***, which is prone to suffer dehydration, with concomitant deprotonation, release of the methoxime product **2** and regeneration of the promoter. Most probably, the steps from **1** to ***iii*** are readily reversible under the mild reaction conditions, whereas once formed compound **2**, it is less likely that it could revert to the starting carbonyl derivative **1** under the same conditions, owing to the relative hydrolytic stability of the oxime ethers [70].

## 4. Conclusion

In conclusion, we have developed an expeditious and efficient CeCl_3_·7H_2_O-based catalytic method for the methoximation of aromatic aldehydes and ketones under mild conditions and demonstrated that the system is also operative on their aliphatic counterparts and for the synthesis of acetophenone oximes. This efficacious reagent reduced substantially the reaction times, the amounts of MeONH_2_·HCl and base required, and afforded very good-to-excellent product yields.

The transformation takes place with an eco-friendly promoter and a sustainable solvent. Further, it was observed that there is no need to employ the anhydrous reagent nor an anhydrous solvent; however, for shorter reaction times, the use of CeCl_3_·7H_2_O in absolute EtOH is preferred.

This catalytic system proved to be robust, and capable to accept a wide variety of aldehydes and ketones. It is also tolerant to electron poor and electron rich substrates, as well as those with some steric demand; in the latter case, at the expense of longer reaction times. These are promising results in the field of synthesis of oximes, which suggest they will find wide use in multistep syntheses of more complex molecules.

## Supplementary Material

ESM - NMR spectra of the compounds

## Supplementary Material

ESM - Literature survey of conditions and yields of other methods

## Data accessibility

All data used in this research are included in figures and tables. The datasets supporting this article have been uploaded as part of the electronic supplementary material.
