# Supplementary material for: A convenient and eco-friendly cerium(III) chloride-catalysed synthesis of methoxime derivatives of aromatic aldehydes and ketones
Source: R Soc Open Sci. 2018 May 23;5(5):180279. doi: 10.1098/rsos.180279 (PMC5990813; doi:10.1098/rsos.180279)
Supplement: ESM - NMR spectra of the compounds [file rsos180279supp1.pdf]

Electronic Supplementary Material (ESI)
for Royal Society Open Science

Supplementary Material for

A convenient and eco-friendly cerium(III) chloride-catalyzed synthesis of methoxime derivatives of aromatic aldehydes and ketones

Iván Cortés, Teodoro S. Kaufman* and Andrea B. J. Bracca*

Instituto de Química Rosario (IQUIR, CONICET-UNR) and Facultad de Ciencias Bioquímicas y Farmacéuticas, Universidad Nacional de Rosario, Suipacha 531, S2002LRK Rosario, Argentina

E-mail: kaufman@iquir-conicet.gov.ar; bracca@iquir-conicet.gov.ar

TABLE OF CONTENTS

**Figure S1.** ^{1}H NMR (300 MHz) spectrum of compound **2a** in $CDCl_3$	S4
**Figure S2.** ^{13}C NMR (75 MHz) spectrum of compound **2a** in $CDCl_3$	S4
**Figure S3.** ^{1}H NMR (300 MHz) spectrum of compound **2b** in $CDCl_3$	S5
**Figure S4.** ^{13}C NMR (75 MHz) spectrum of compound **2b** in $CDCl_3$	S5
**Figure S5.** ^{1}H NMR (300 MHz) spectrum of compound **2c** in $CDCl_3$	S6
**Figure S6.** ^{13}C NMR (75 MHz) spectrum of compound **2c** in $CDCl_3$	S6
**Figure S7.** ^{1}H NMR (300 MHz) spectrum of compound **2d** in $CDCl_3$	S7
**Figure S8.** ^{13}C NMR (75 MHz) spectrum of compound **2d** in $CDCl_3$	S7
**Figure S9.** ^{1}H NMR (300 MHz) spectrum of compound **2e** in $CDCl_3$	S8
**Figure S10.** ^{13}C NMR (75 MHz) spectrum of compound **2e** in $CDCl_3$	S8
**Figure S11.** ^{1}H NMR (300 MHz) spectrum of compound **2f** in $CDCl_3$	S9
**Figure S12.** ^{13}C NMR (75 MHz) spectrum of compound **2f** in $CDCl_3$	S9
**Figure S13.** ^{1}H NMR (300 MHz) spectrum of compound **2g** in $CDCl_3$	S10
**Figure S14.** ^{13}C NMR (75 MHz) spectrum of compound **2g** in $CDCl_3$	S10
**Figure S15.** ^{1}H NMR (300 MHz) spectrum of compound **2h** in $CDCl_3$	S11
**Figure S16.** ^{13}C NMR (75 MHz) spectrum of compound **2h** in $CDCl_3$	S11
**Figure S17.** COSY spectrum of compound **2h** in $CDCl_3$	S12
**Figure S18.** HSQC spectrum of compound **2h** in $CDCl_3$	S12
**Figure S19.** ^{1}H NMR (300 MHz) spectrum of compound **2i** in $CDCl_3$	S13
**Figure S20.** ^{13}C NMR (75 MHz) spectrum of compound **2i** in $CDCl_3$	S13
**Figure S21.** HSQC spectrum of compound **2i** in $CDCl_3$	S14
**Figure S22.** ^{1}H NMR (300 MHz) spectrum of compound **2j** in $CDCl_3$	S15
**Figure S23.** ^{13}C NMR (75 MHz) spectrum of compound **2j** in $CDCl_3$	S15
**Figure S24.** COSY spectrum of compound **2j** in $CDCl_3$	S16
**Figure S25.** HSQC spectrum of compound **2j** in $CDCl_3$	S16
**Figure S26.** ^{1}H NMR (300 MHz) spectrum of compound **2k** in $CDCl_3$	S17
**Figure S27.** ^{13}C NMR (75 MHz) spectrum of compound **2k** in $CDCl_3$	S17
**Figure S28.** ^{1}H NMR (300 MHz) spectrum of compound **2l** in $CDCl_3$	S18
**Figure S29.** ^{13}C NMR (75 MHz) spectrum of compound **2l** in $CDCl_3$	S18
**Figure S30.** COSY spectrum of compound **2l** in $CDCl_3$	S19
**Figure S31.** HSQC spectrum of compound **2l** in $CDCl_3$	S19

**Figure S32.** ^{1}H NMR (300 MHz) spectrum of compound **2m** in $CDCl_3$	S20
**Figure S33.** ^{13}C NMR (75 MHz) spectrum of compound **2m** in $CDCl_3$	S20
**Figure S34.** ^{1}H NMR (300 MHz) spectrum of compound **2n** in $CDCl_3$	S21
**Figure S35.** ^{13}C NMR (75 MHz) spectrum of compound **2n** in $CDCl_3$	S21
**Figure S36.** ^{1}H NMR (300 MHz) spectrum of compound **2o** in $CDCl_3$	S22
**Figure S37.** ^{13}C NMR (75 MHz) spectrum of compound **2o** in $CDCl_3$	S22
**Figure S38.** ^{1}H NMR (300 MHz) spectrum of compound **2p** in $CDCl_3$	S23
**Figure S39.** ^{13}C NMR (75 MHz) spectrum of compound **2p** in $CDCl_3$	S23
**Figure S40.** ^{1}H NMR (300 MHz) spectrum of compound **2q** in $CDCl_3$	S24
**Figure S41.** ^{13}C NMR (75 MHz) spectrum of compound **2q** in $CDCl_3$	S24
**Figure S42.** ^{1}H NMR (300 MHz) spectrum of compound **2r** in $CDCl_3$	S25
**Figure S43.** ^{13}C NMR (75 MHz) spectrum of compound **2r** in $CDCl_3$	S25
**Figure S44.** ^{1}H NMR (300 MHz) spectrum of compound **2s** in $CDCl_3$	S26
**Figure S45.** ^{13}C NMR (75 MHz) spectrum of compound **2s** in $CDCl_3$	S26
**Figure S46.** ^{1}H NMR (300 MHz) spectrum of compound **2t** in $CDCl_3$	S27
**Figure S47.** ^{13}C NMR (75 MHz) spectrum of compound **2t** in $CDCl_3$	S27
**Figure S48.** COSY spectrum of compound **2t** in $CDCl_3$	S28
**Figure S49.** HSQC spectrum of compound **2t** in $CDCl_3$	S28
**Figure S50.** ^{1}H NMR (300 MHz) spectrum of compound **2u** in $CDCl_3$	S29
**Figure S51.** ^{13}C NMR (75 MHz) spectrum of compound **2u** in $CDCl_3$	S29
**Figure S52.** COSY spectrum of compound **2u** in $CDCl_3$	S30
**Figure S53.** HSQC spectrum of compound **2u** in $CDCl_3$	S30
**Figure S54.** ^{1}H NMR (300 MHz) spectrum of compound **2v** in $CDCl_3$	S31
**Figure S55.** ^{13}C NMR (75 MHz) spectrum of compound **2v** in $CDCl_3$	S31
**Figure S56.** HSQC spectrum of compound **2v** in $CDCl_3$	S32
**Figure S57.** ^{1}H NMR (300 MHz) spectrum of compound **2w** in $CDCl_3$	S32
**Figure S58.** ^{13}C NMR (75 MHz) spectrum of compound **2w** in $CDCl_3$	S33
**Figure S59.** COSY spectrum of compound **2w** in $CDCl_3$	S34
**Figure S60.** HSQC spectrum of compound **2w** in $CDCl_3$	S34

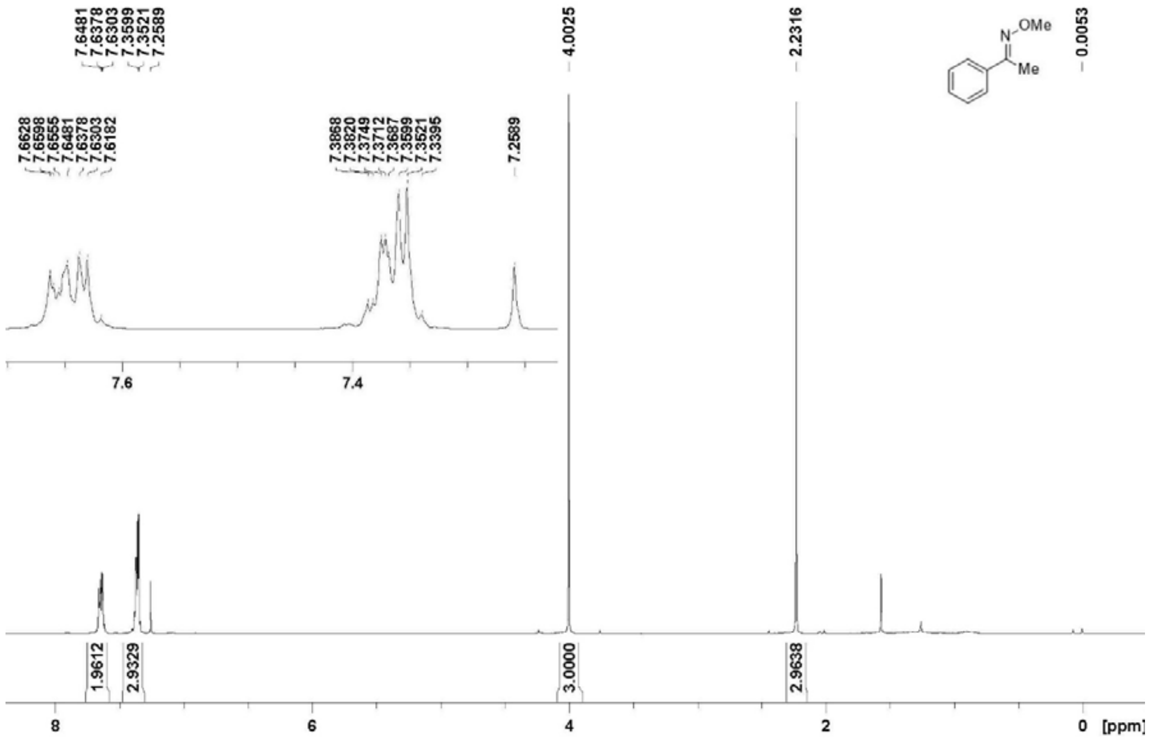

Figure S1. ^{1}H NMR (300 MHz) spectrum of compound **2a**.

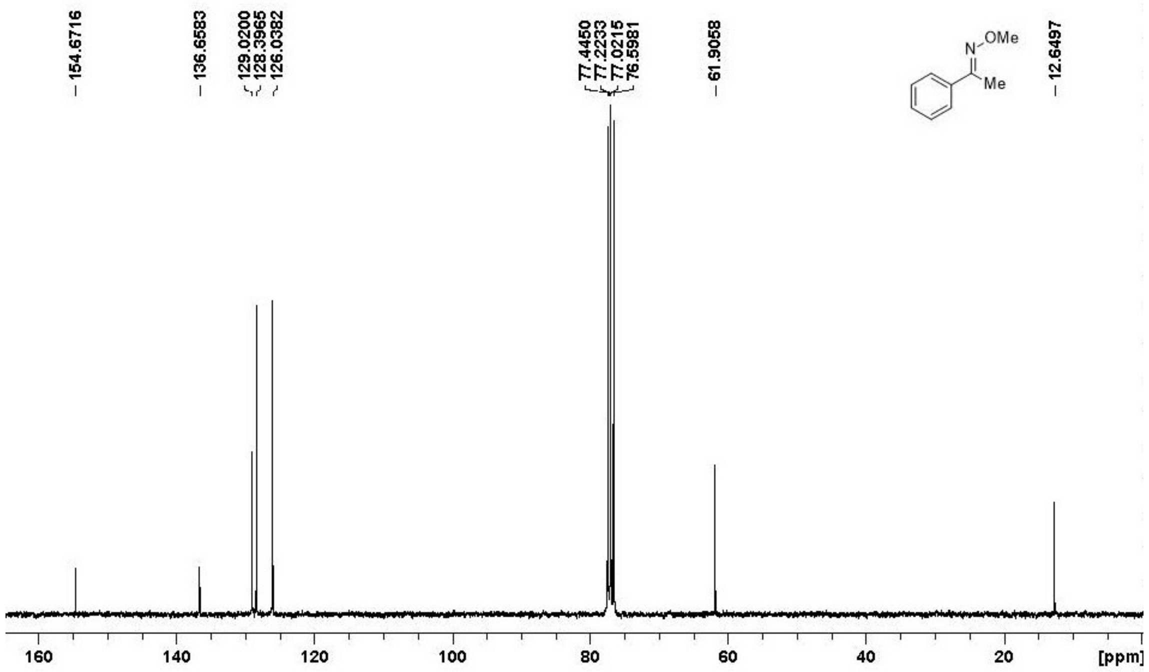

Figure S2. ^{13}C NMR (75 MHz) spectrum of compound **2a**.

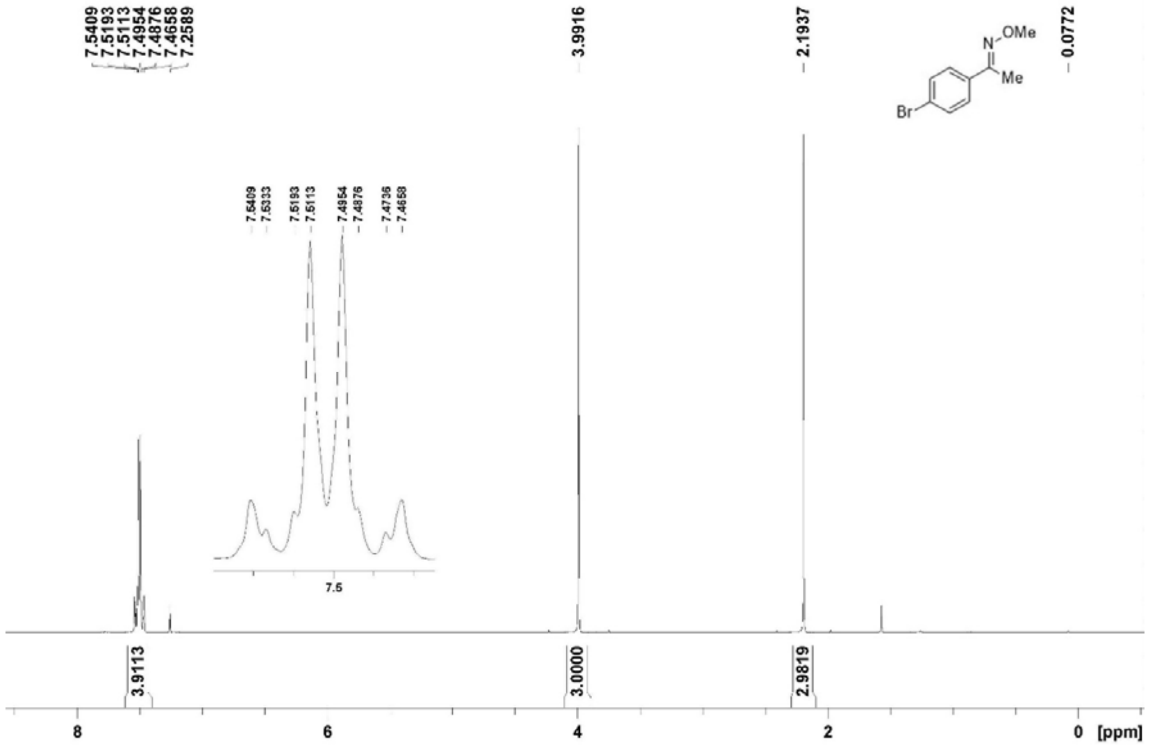

Figure S3. ^{1}H NMR (300 MHz) spectrum of compound **2b**.

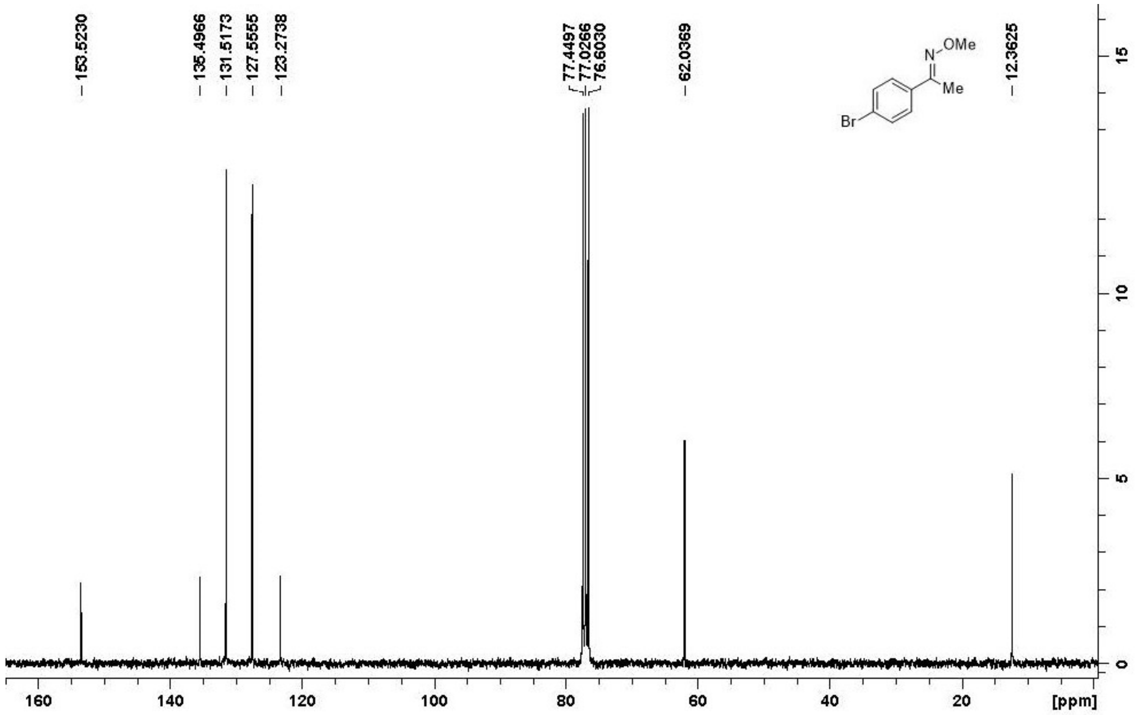

Figure S4. ^{13}C NMR (75 MHz) spectrum of compound **2b**.

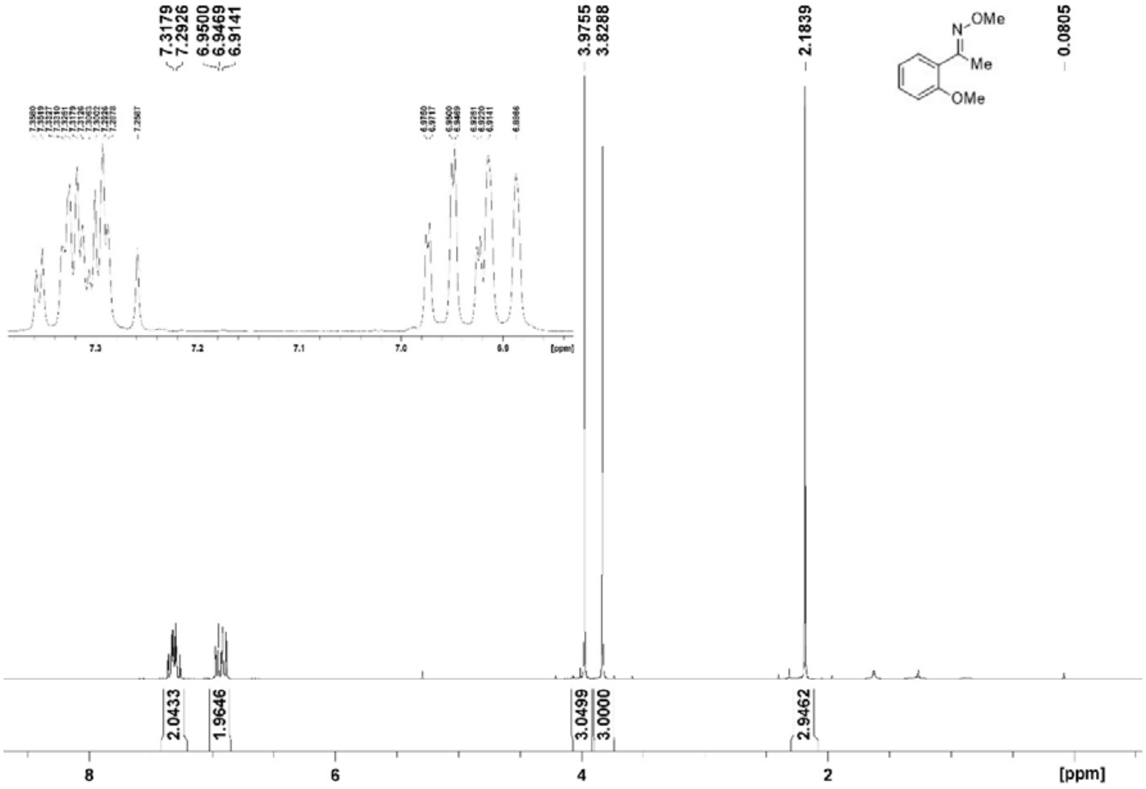

Figure S5. ^{1}H NMR (300 MHz) spectrum of compound **2c**.

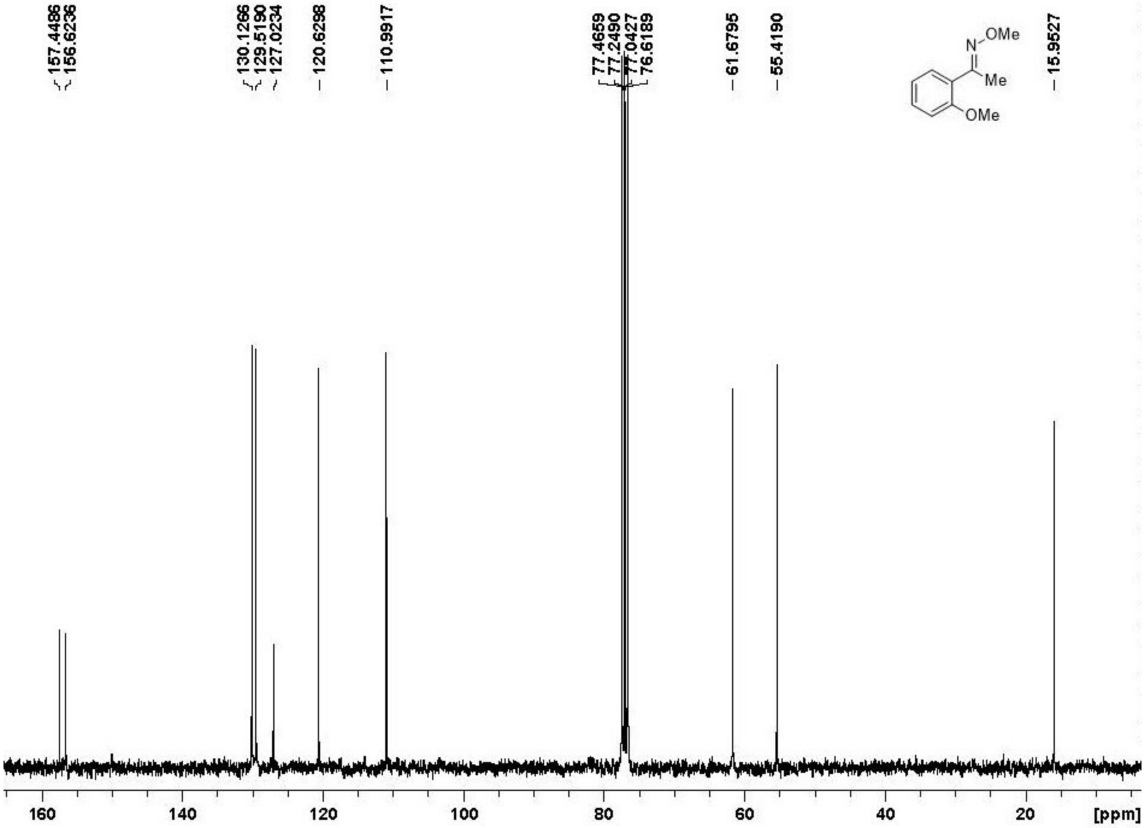

Figure S6. ^{13}C NMR (75 MHz) spectrum of compound **2c**.

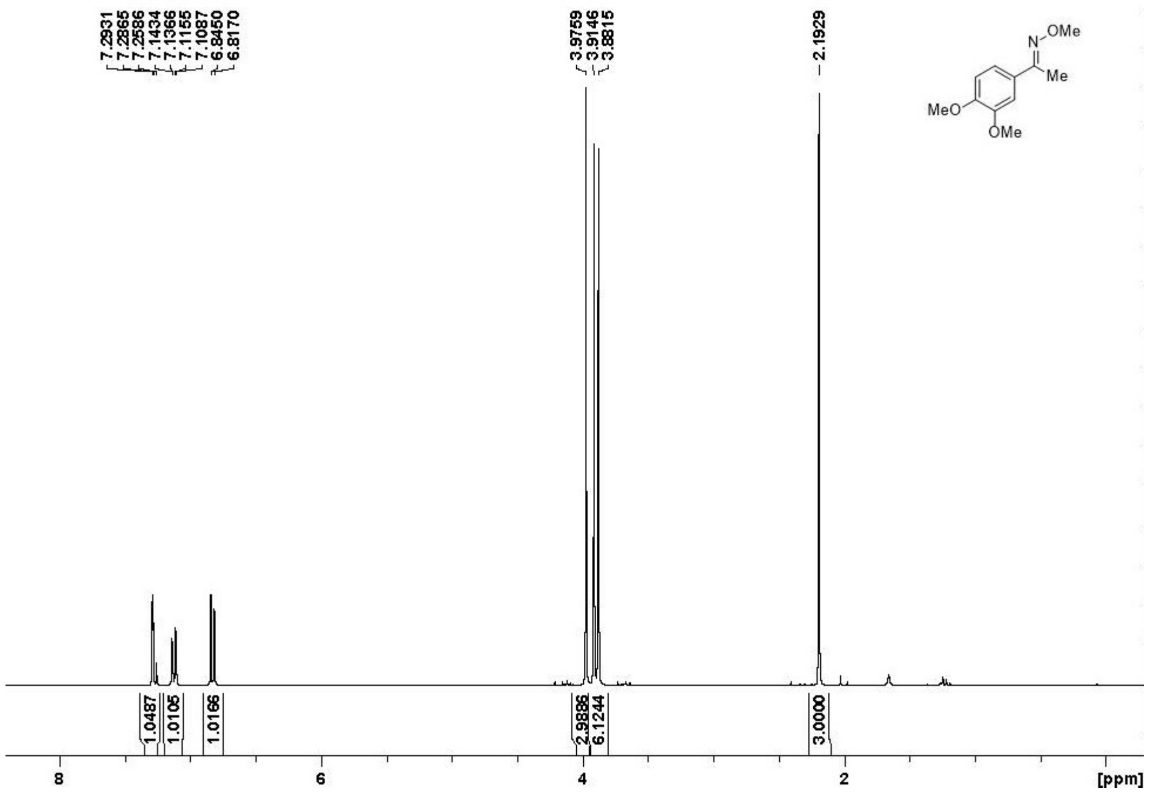

Figure S7. ^{1}H NMR (300 MHz) spectrum of compound **2d**.

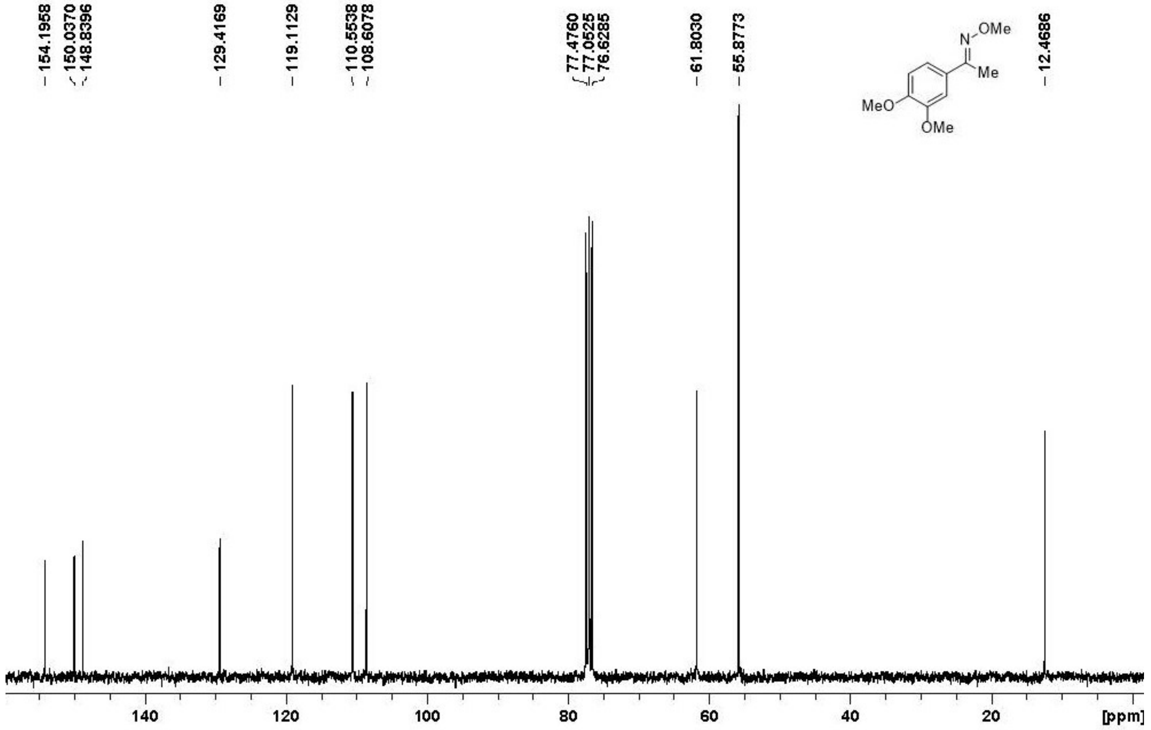

Figure S8. ^{13}C NMR (75 MHz) spectrum of compound **2d**.

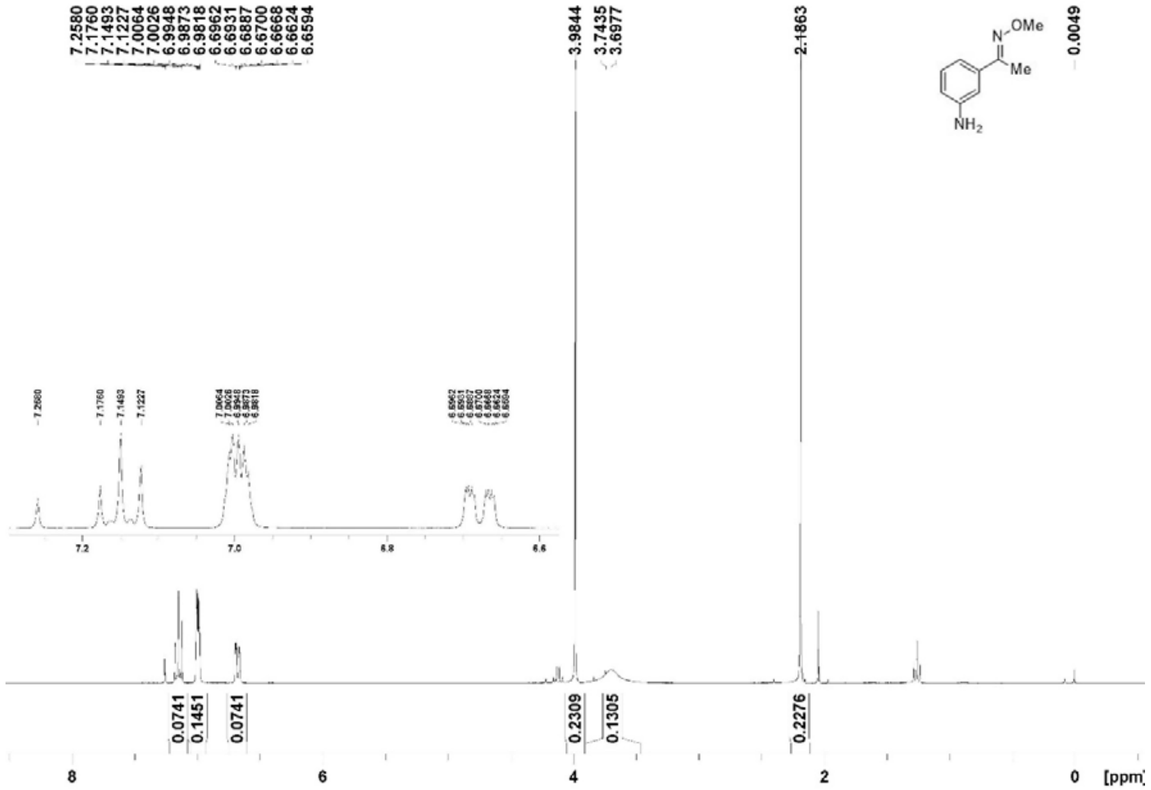

Figure S9. ^{1}H NMR (300 MHz) spectrum of compound **2e**.

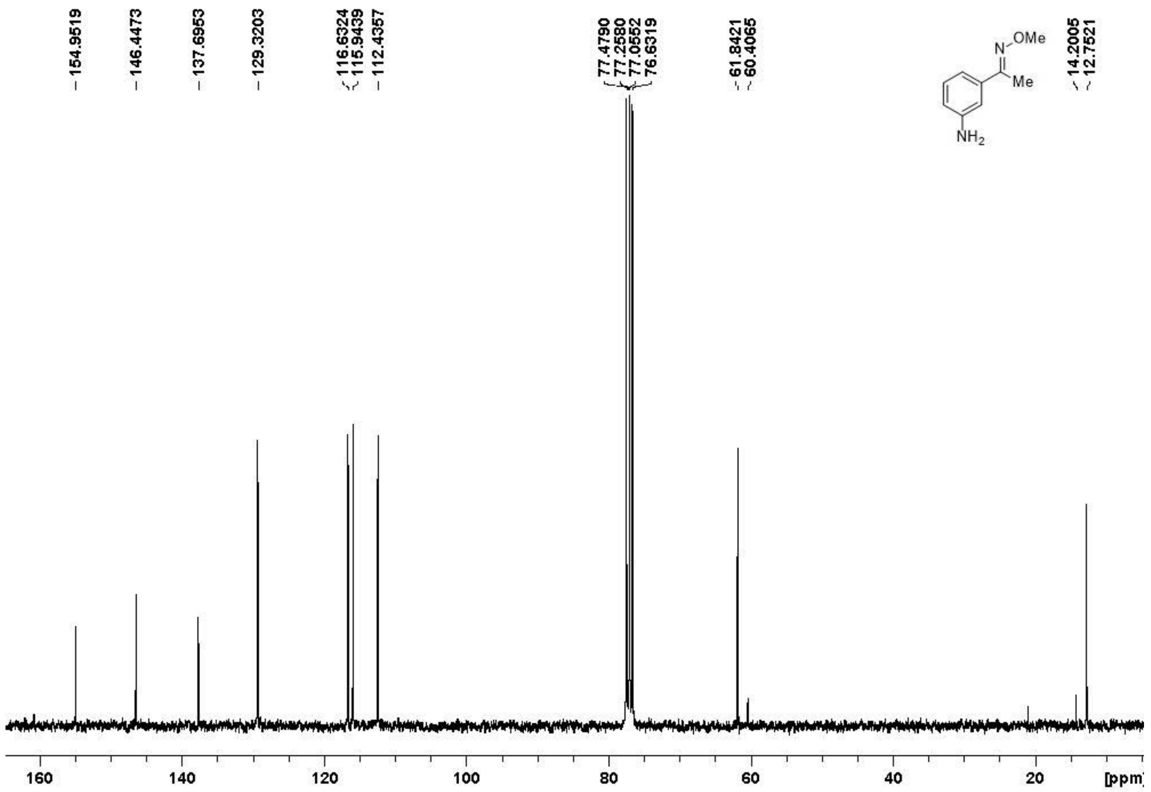

Figure S10. ^{13}C NMR (75 MHz) spectrum of compound **2e**.

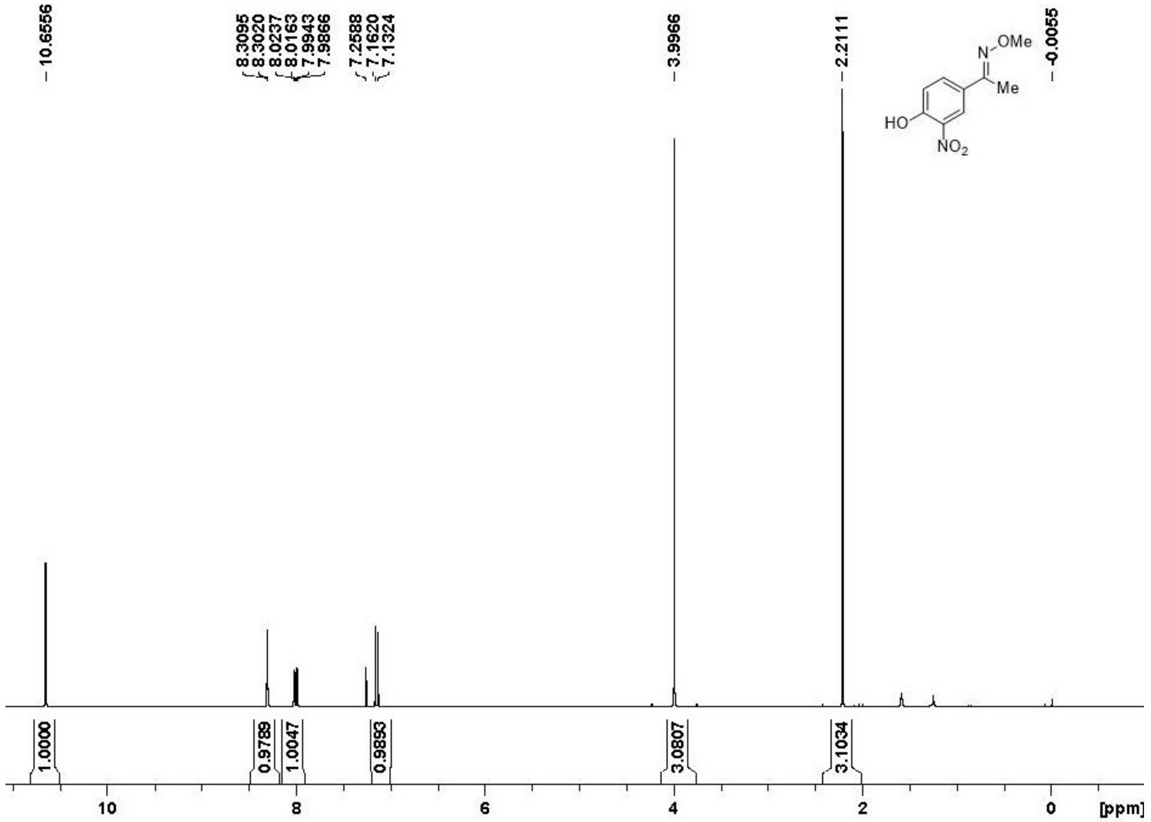

Figure S11. ^{1}H NMR (300 MHz) spectrum of compound **2f**.

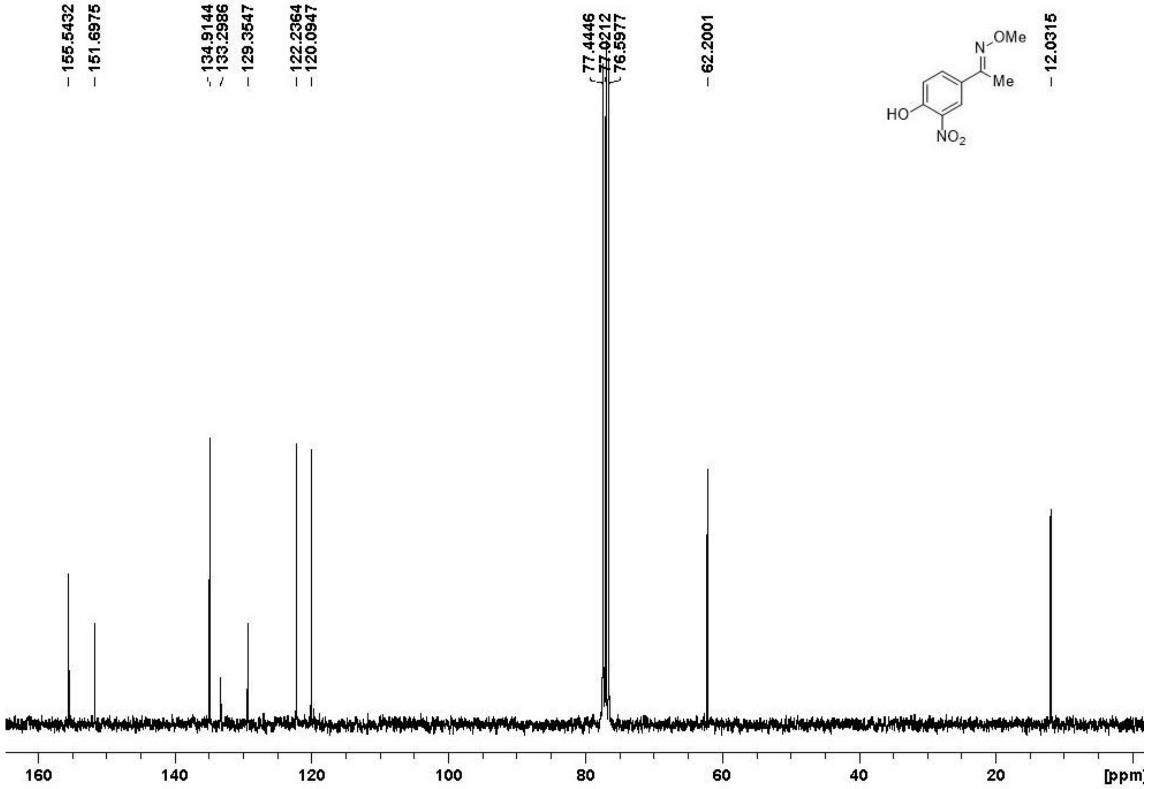

Figure S12. ^{13}C NMR (75 MHz) spectrum of compound **2f**.

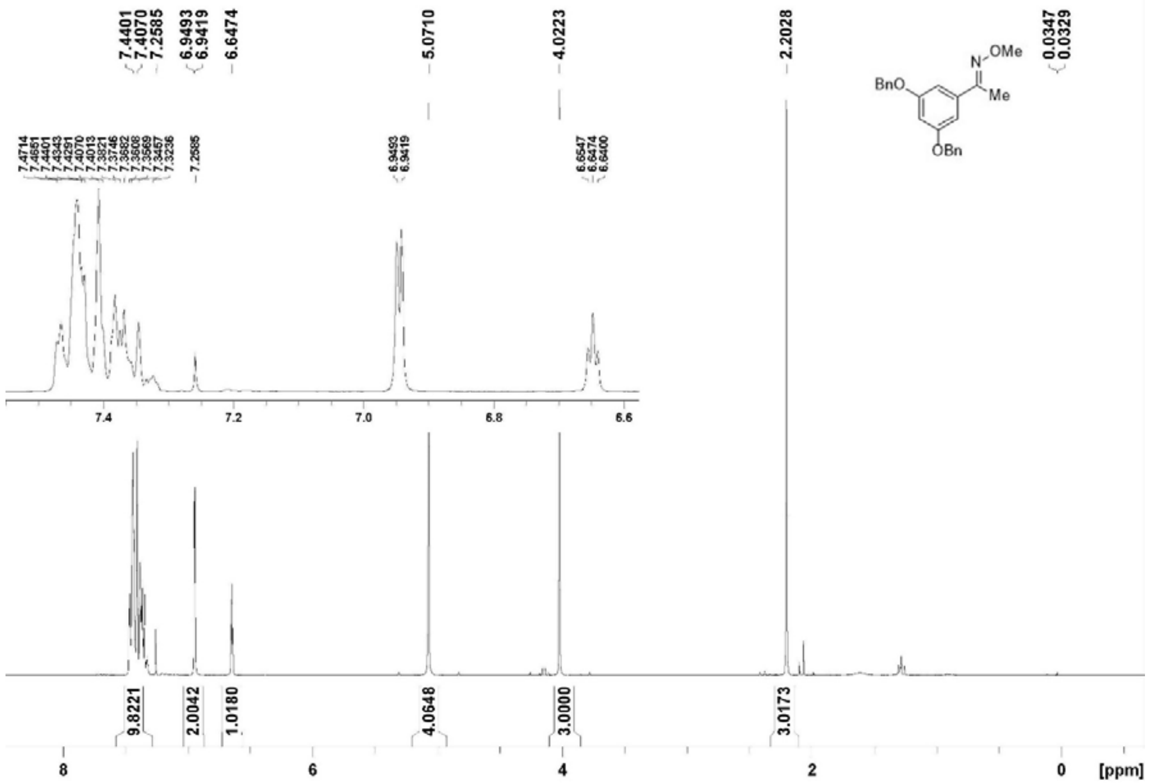

Figure S13. ^{1}H NMR (300 MHz) spectrum of compound **2g**.

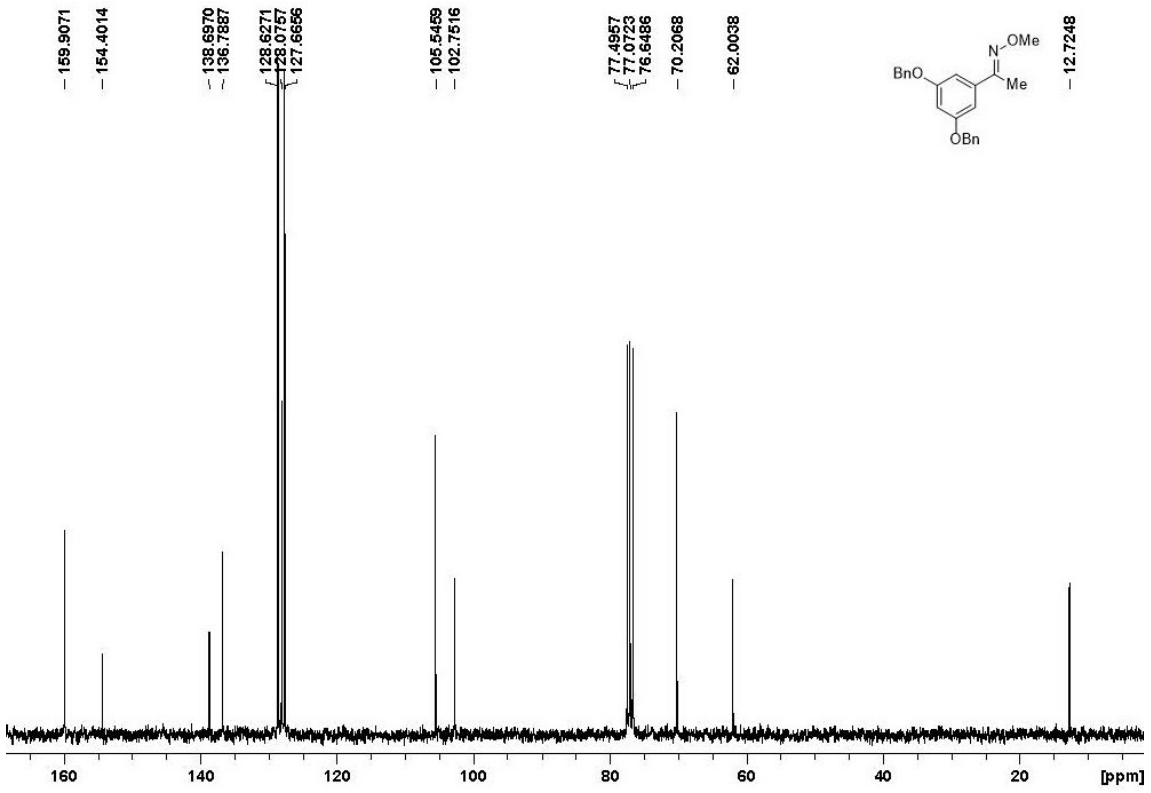

Figure S14. ^{13}C NMR (75 MHz) spectrum of compound **2g**.

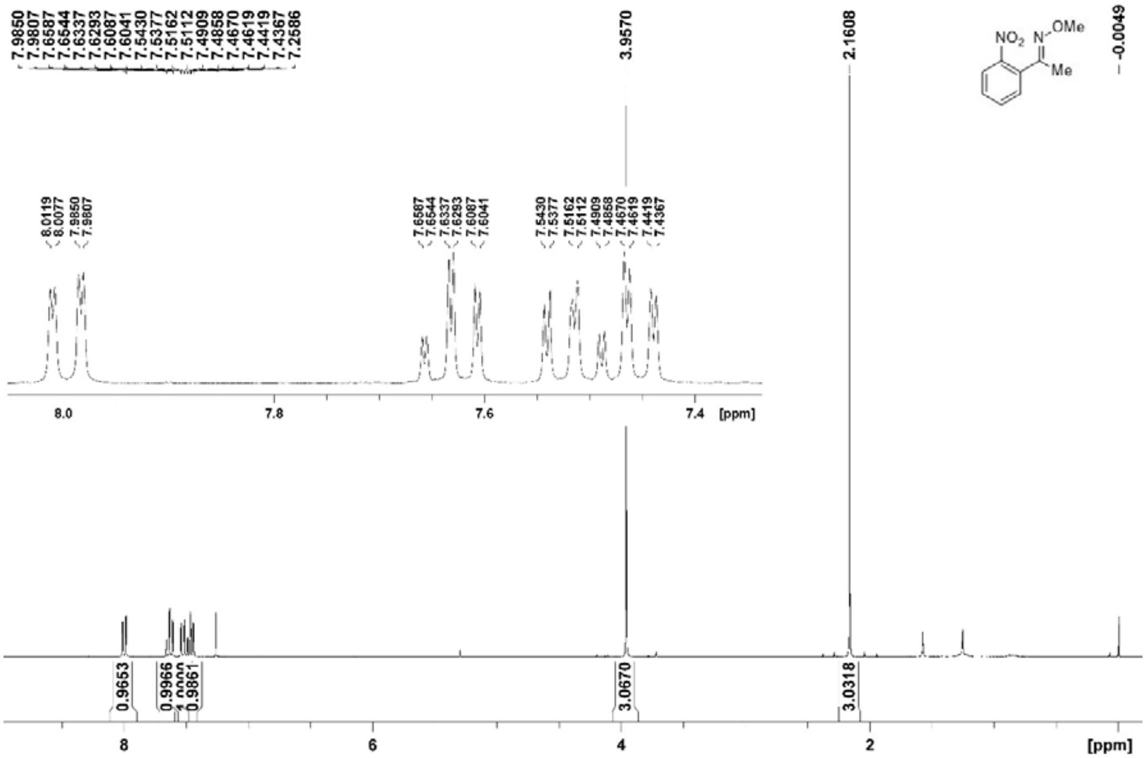

Figure S15. ^{1}H NMR (300 MHz) spectrum of compound **2h**.

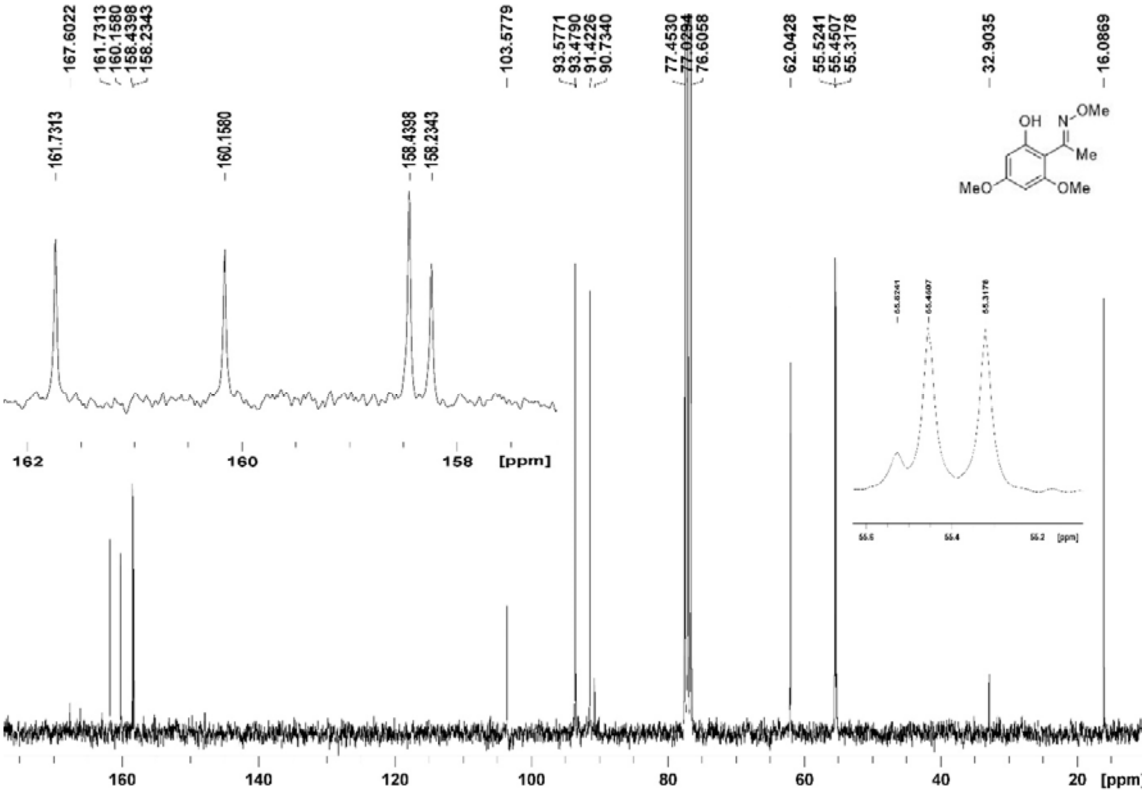

Figure S16. ^{13}C NMR (75 MHz) spectrum of compound **2h**.

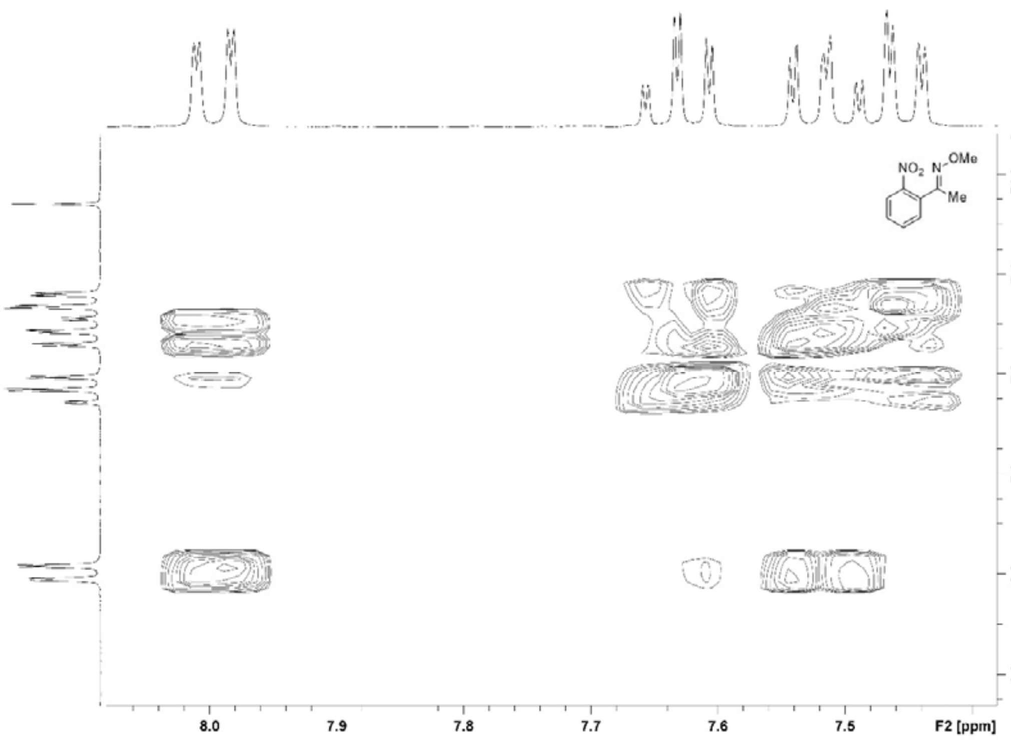

Figure S17. COSY spectrum of compound **2h**.

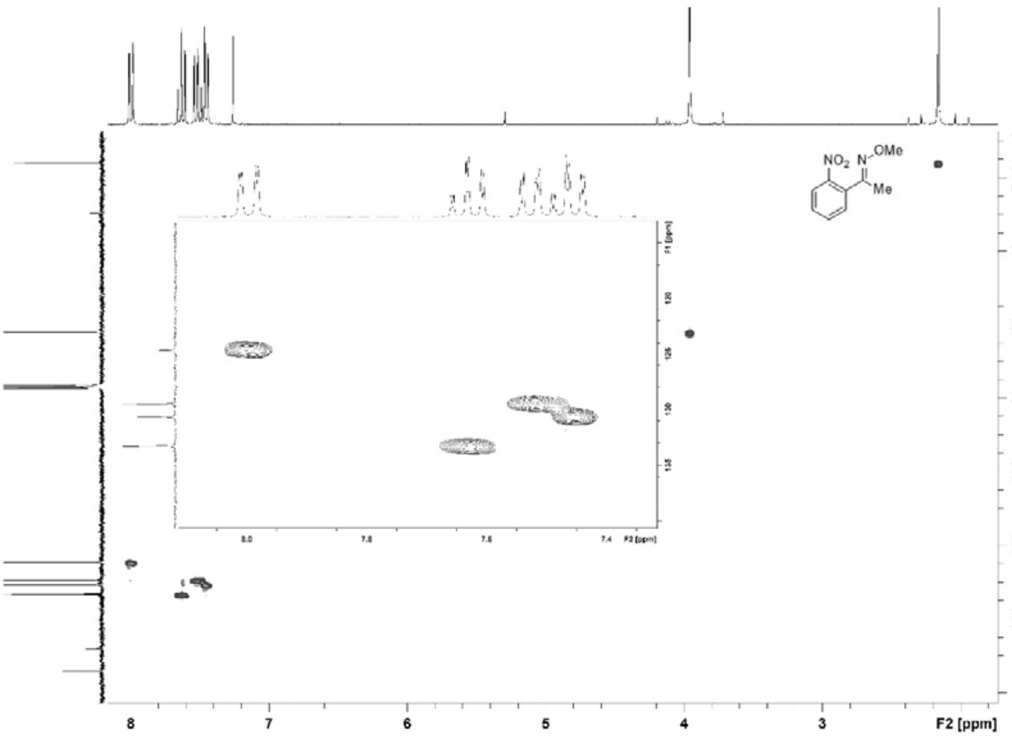

Figure S18. HSQC spectrum of compound **2h**.

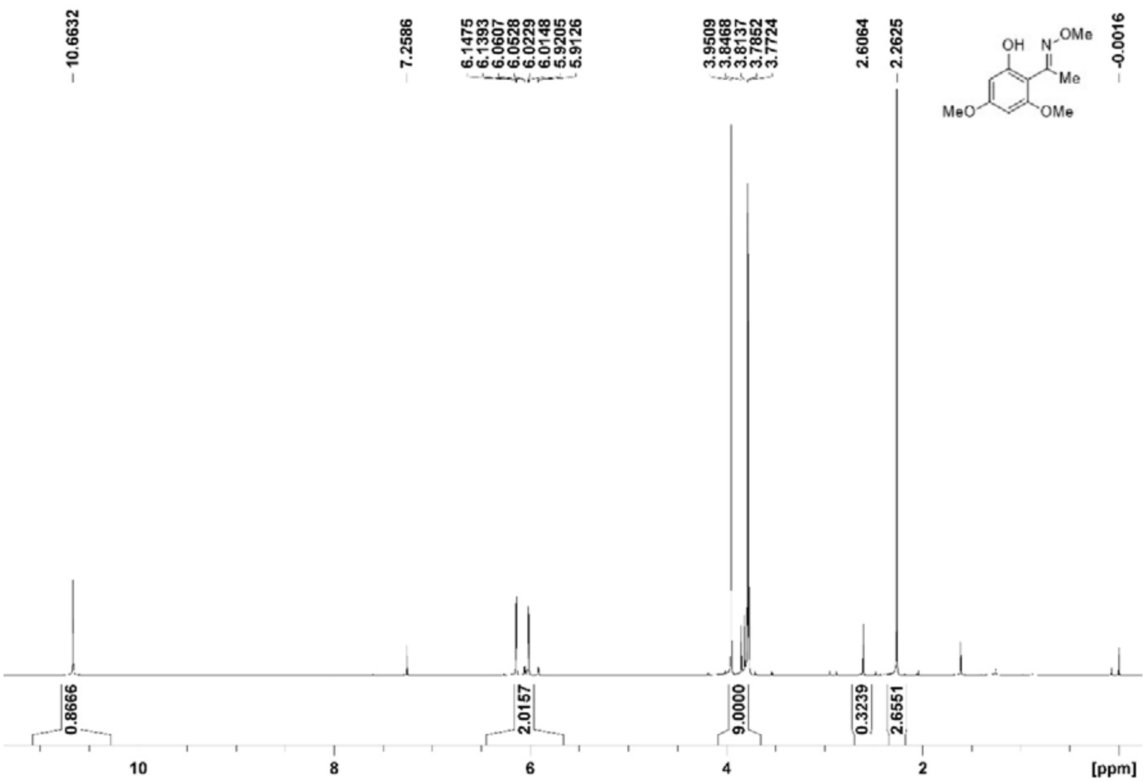

Figure S19. ^{1}H NMR (300 MHz) spectrum of compound **2i**.

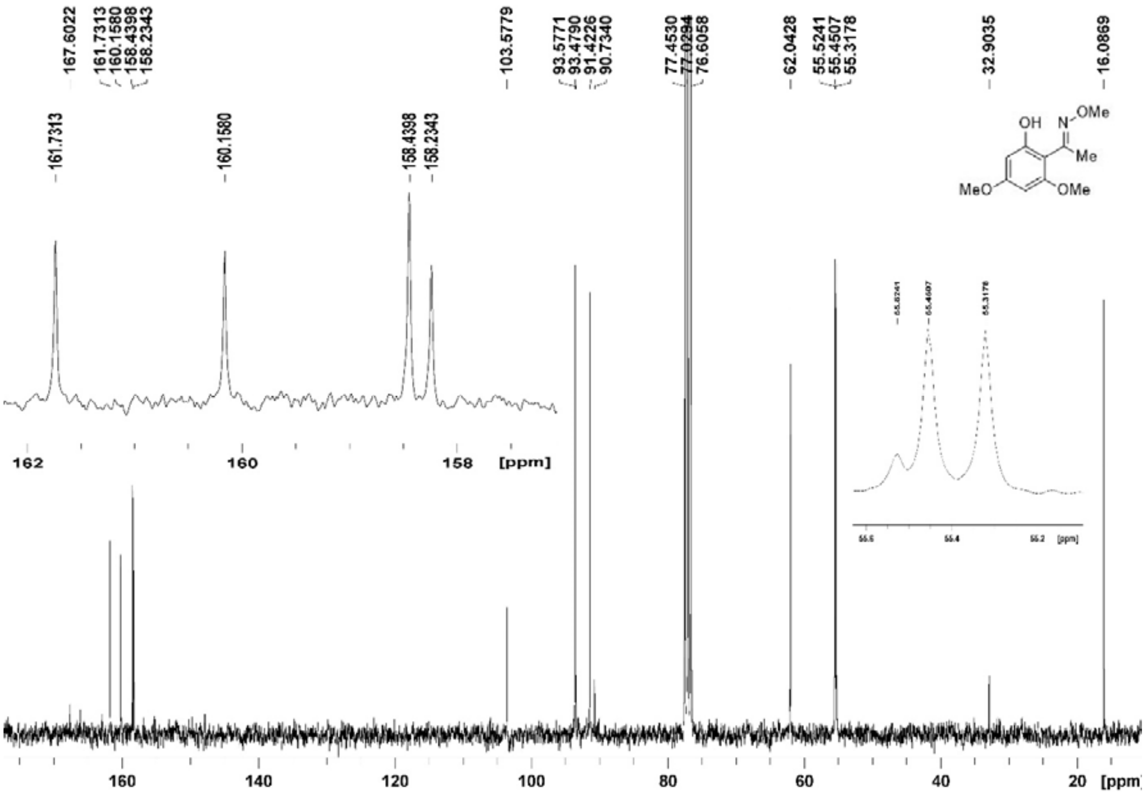

Figure S20. ^{13}C NMR (75 MHz) spectrum of compound **2i**.

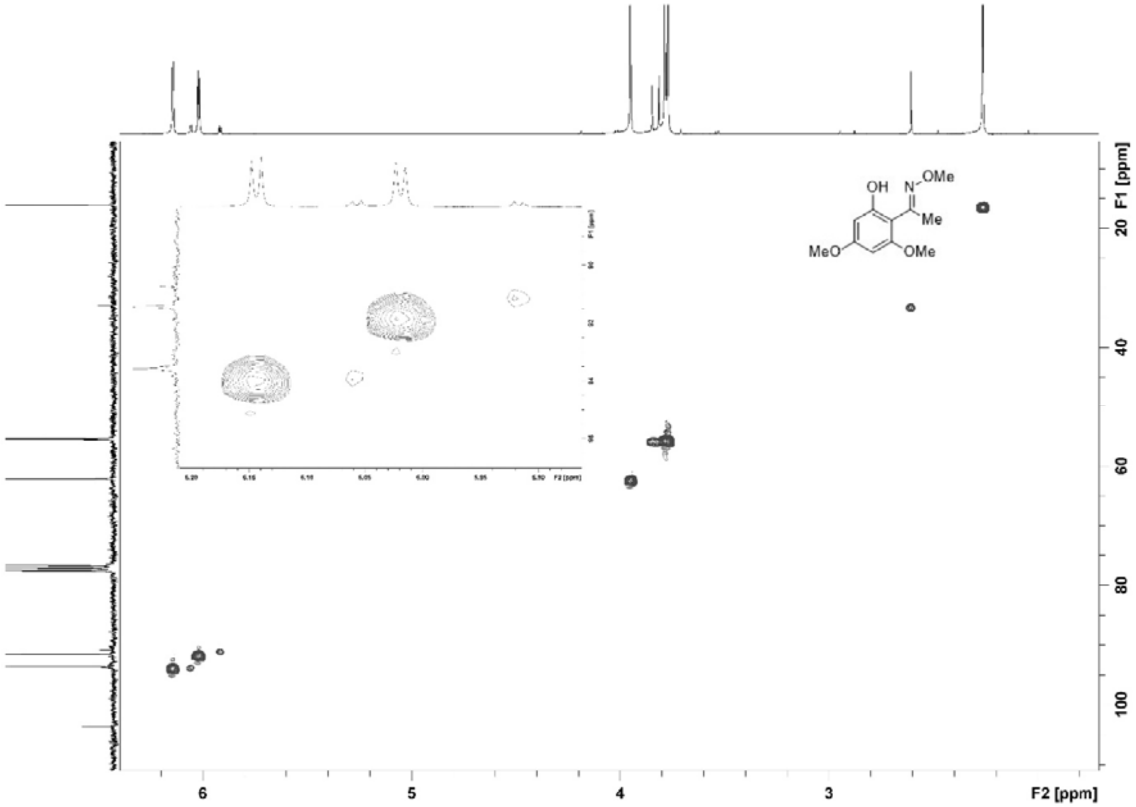

Figure S21. HSQC spectrum of compound **2i**.

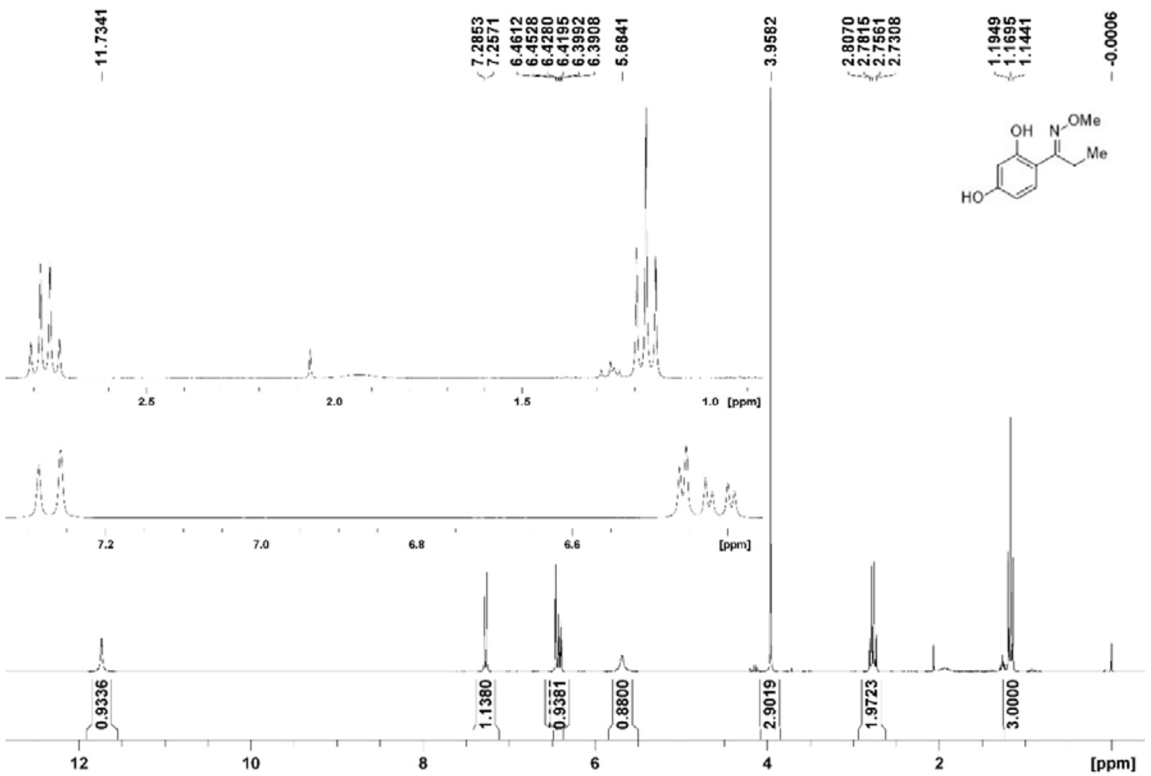

Figure S22. ^{1}H NMR (300 MHz) spectrum of compound **2j**.

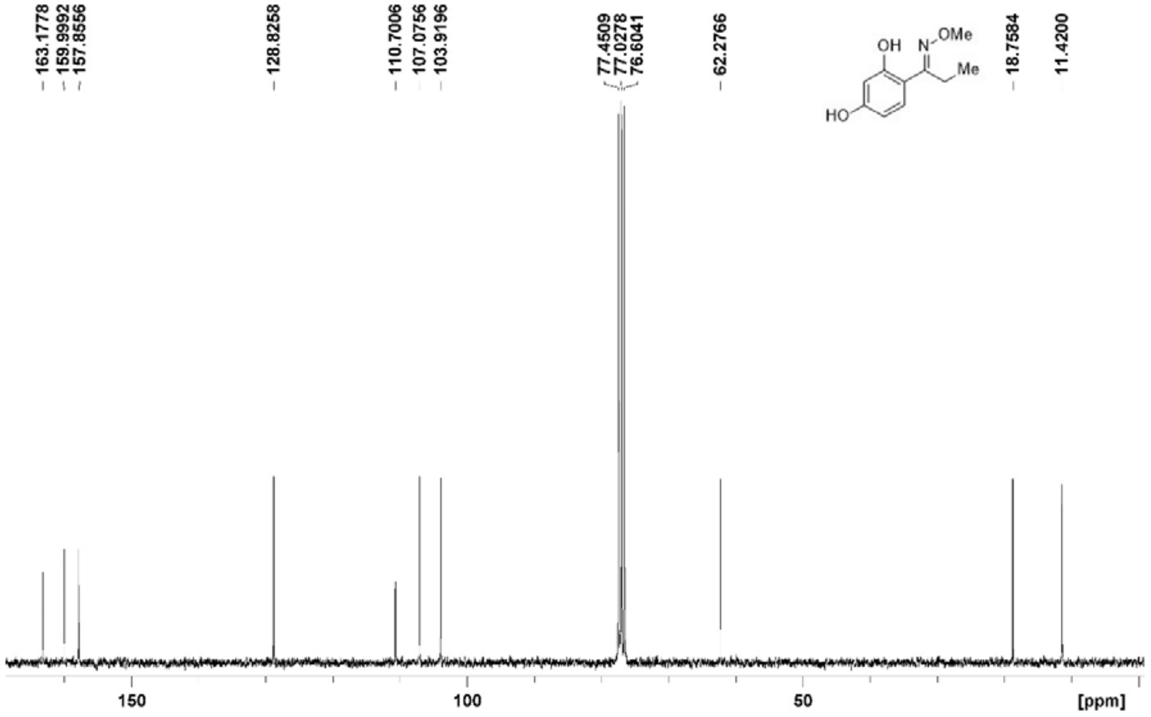

Figure S23. ^{13}C NMR (75 MHz) spectrum of compound **2j**.

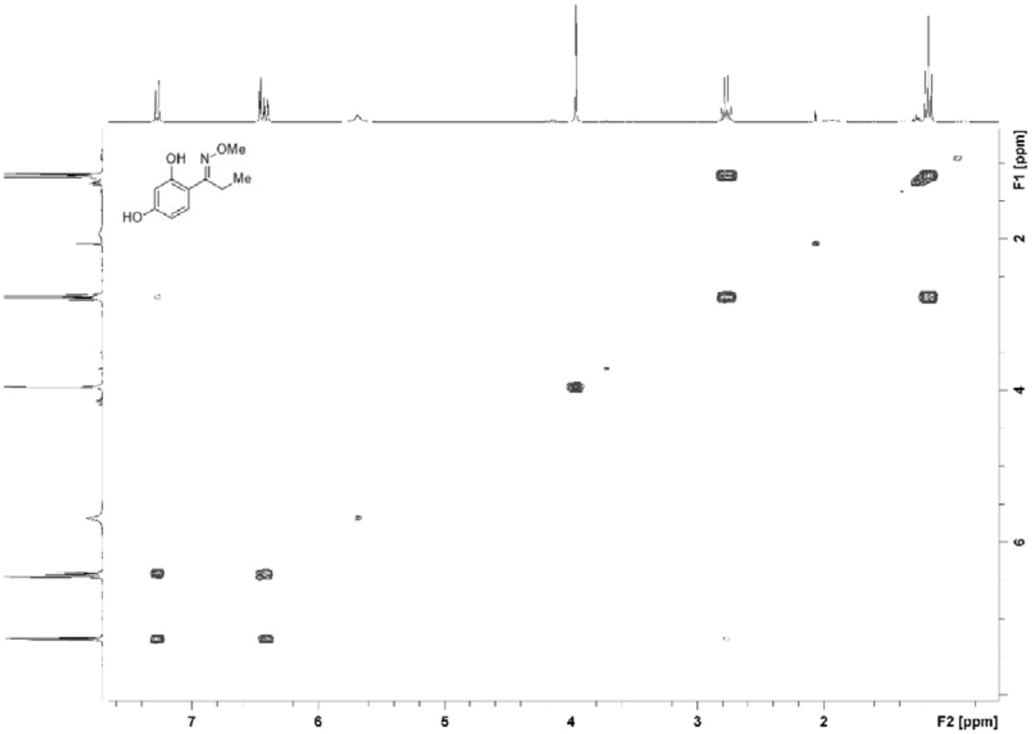

Figure S24. COSY spectrum of compound **2j**.

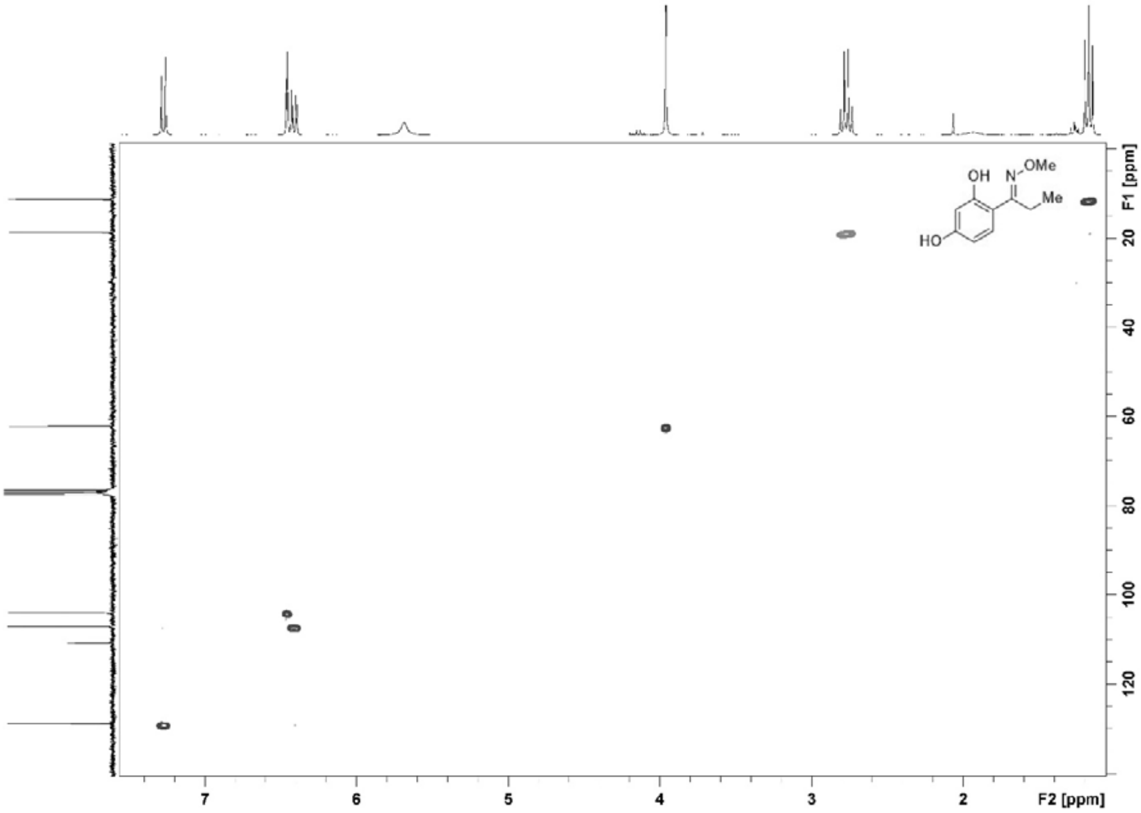

Figure S25. HSQC spectrum of compound **2j**.

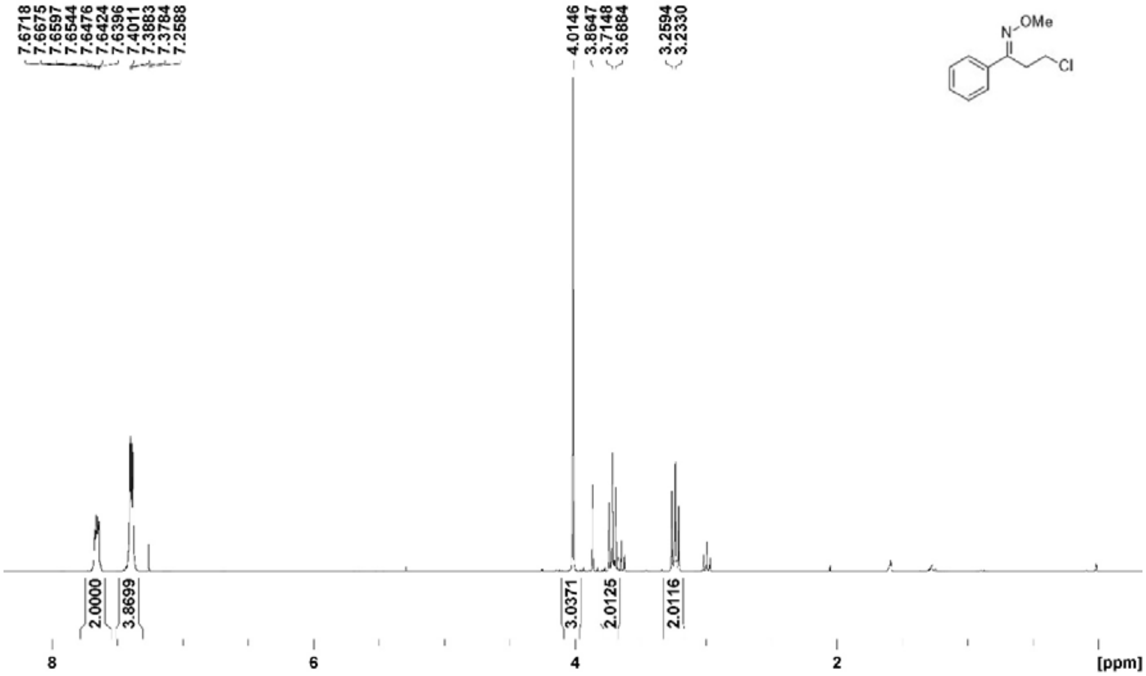

Figure S26. ^{1}H NMR (300 MHz) spectrum of compound **2k**.

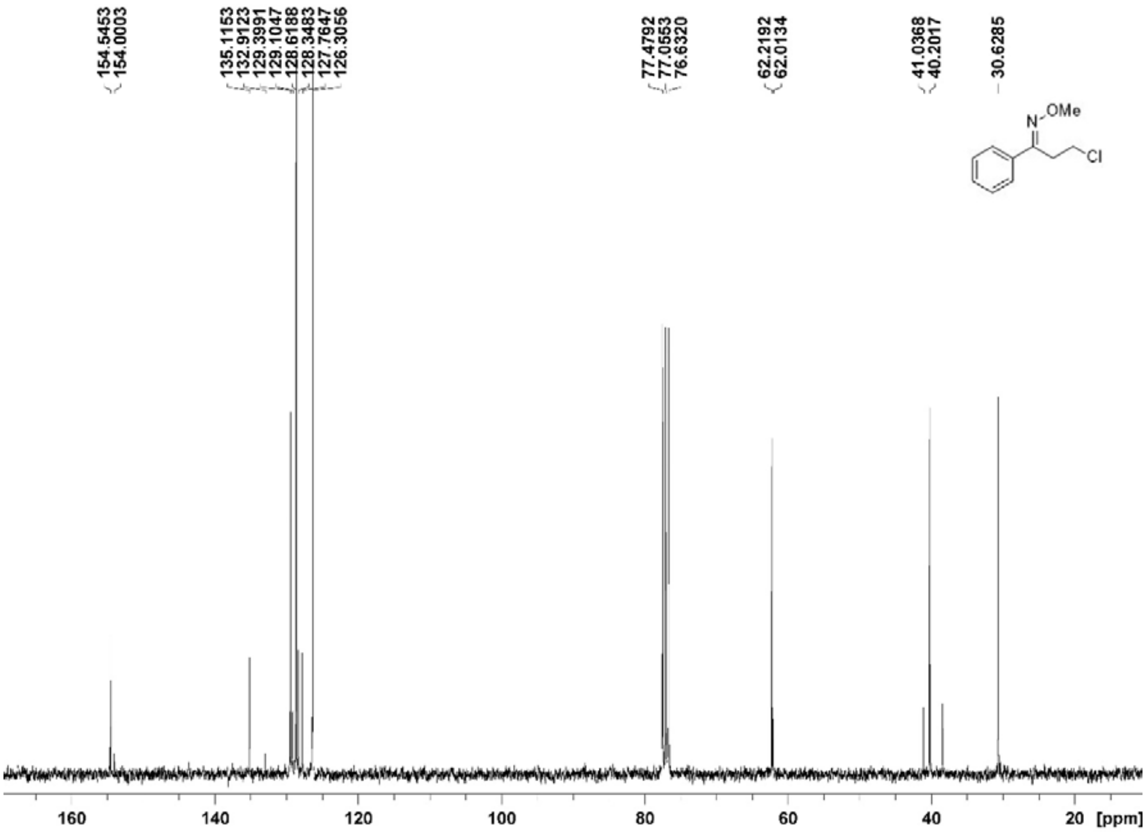

Figure S27. ^{13}C NMR (75 MHz) spectrum of compound **2k**.

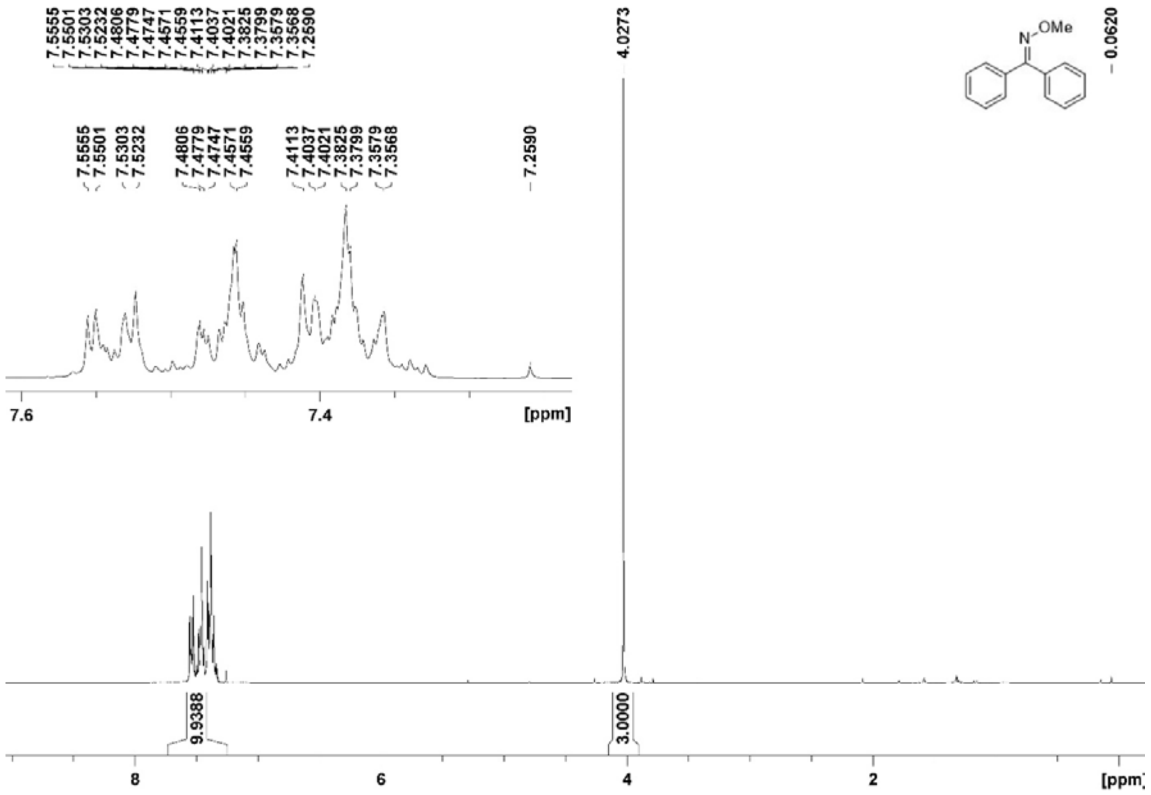

Figure S28. ^{1}H NMR (300 MHz) spectrum of compound **2l**.

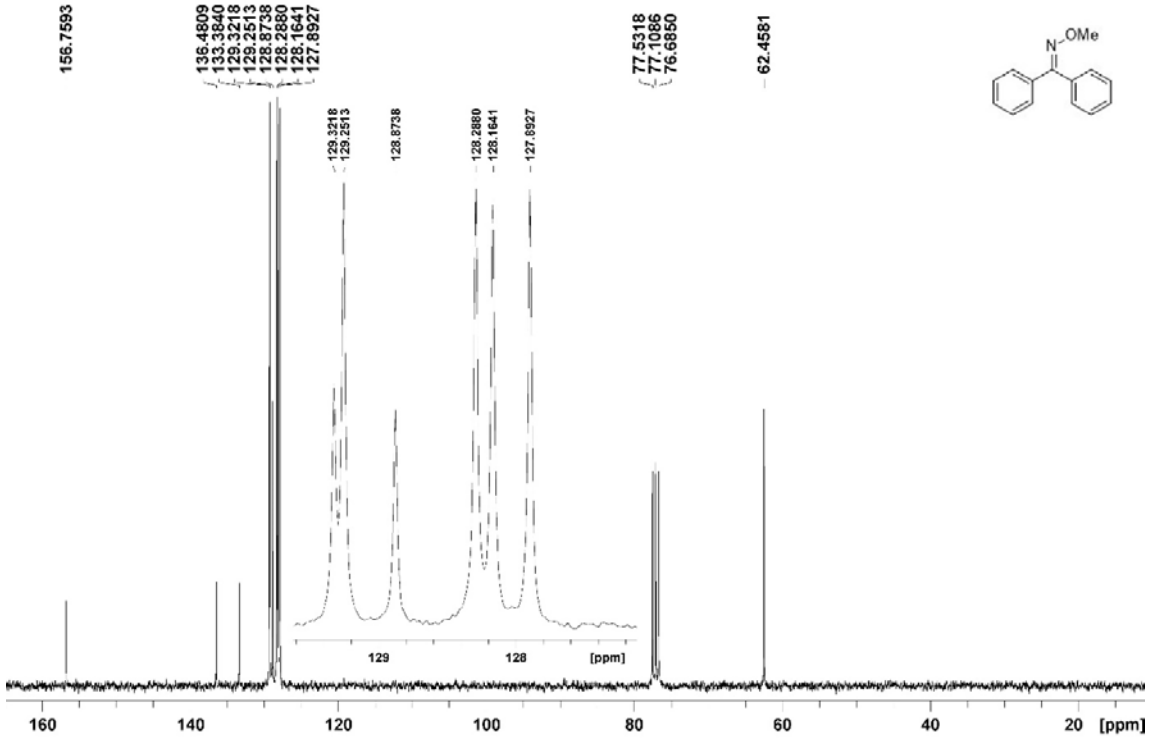

Figure S29. ^{13}C NMR (75 MHz) spectrum of compound **2l**.

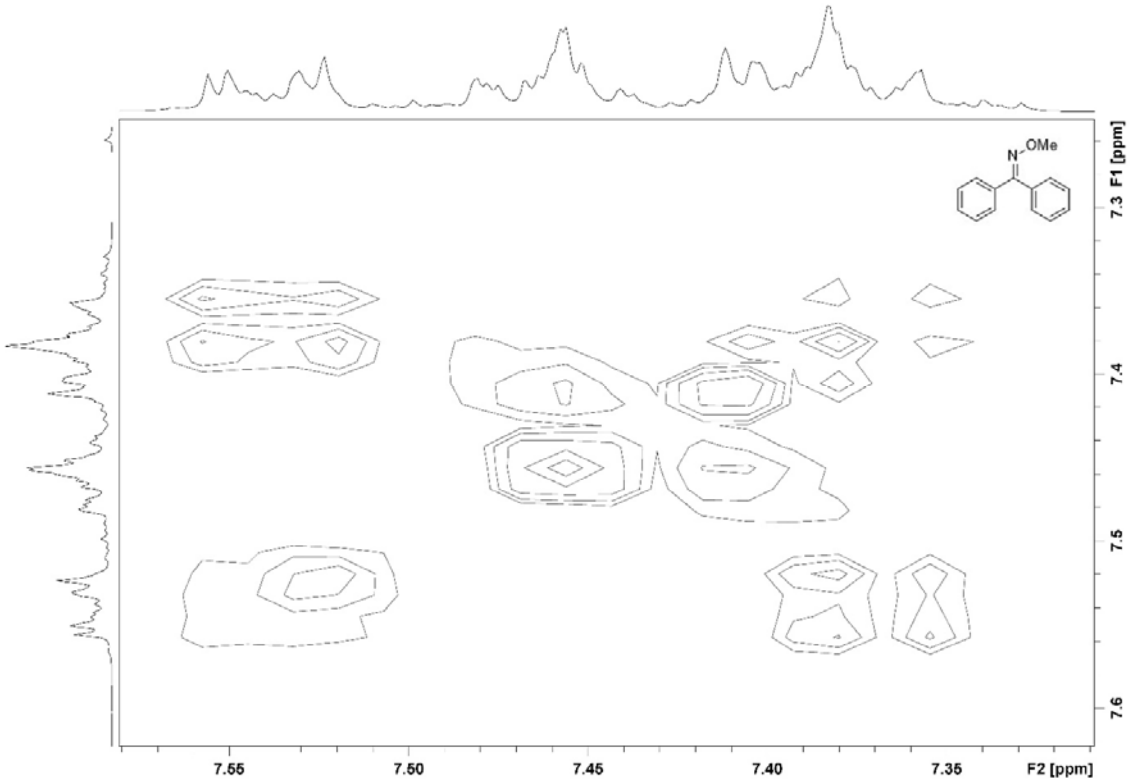

Figure S30. COSY spectrum of compound **2l**.

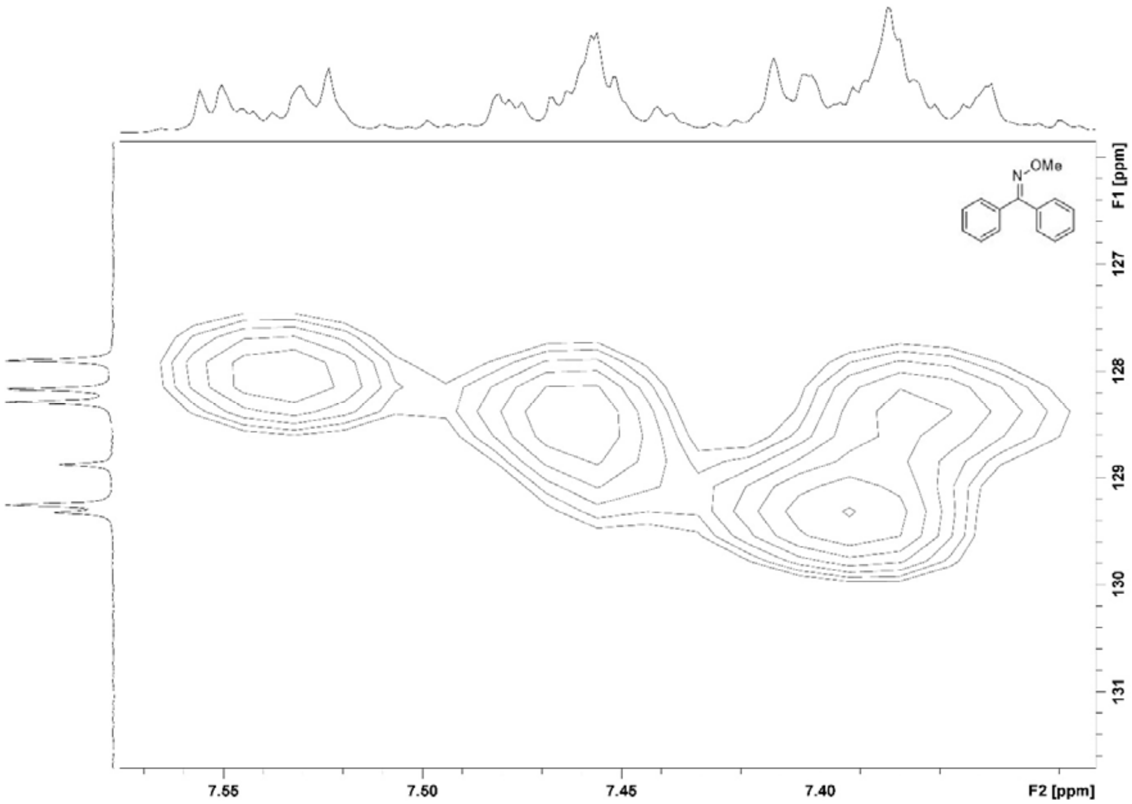

Figure S31. HSQC spectrum of compound **2l**.

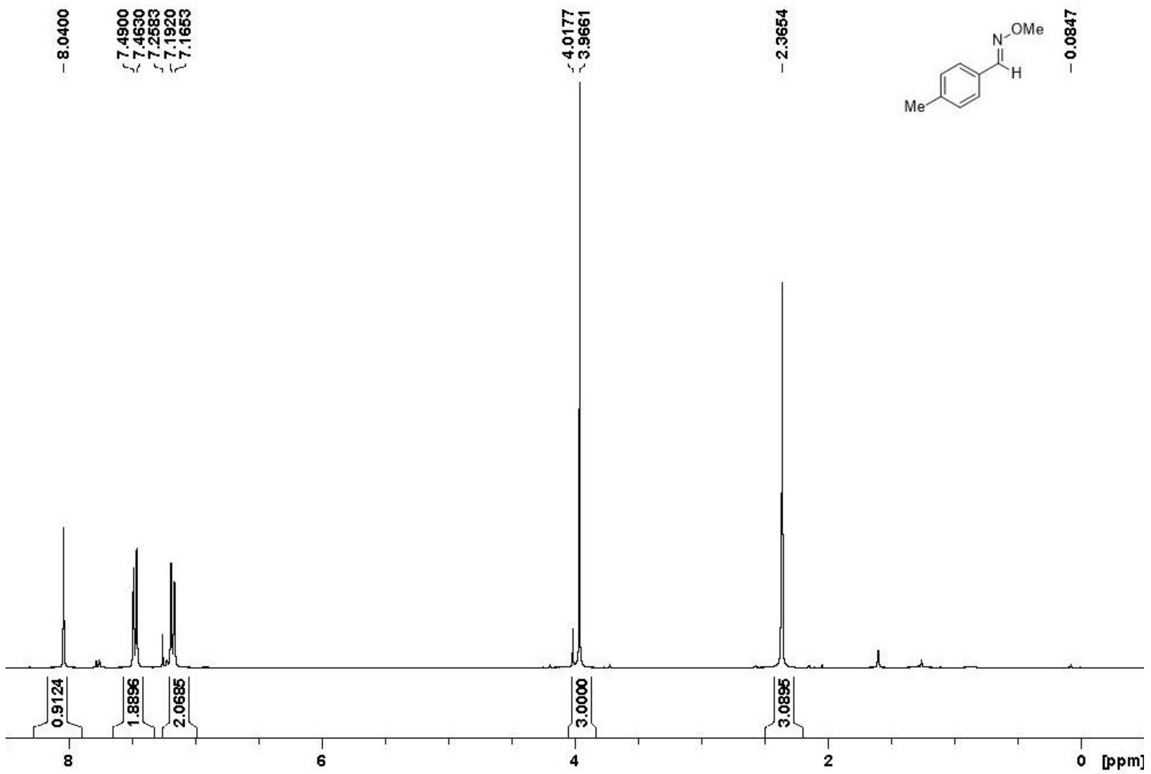

Figure S32. ^{1}H NMR (300 MHz) spectrum of compound **2m**.

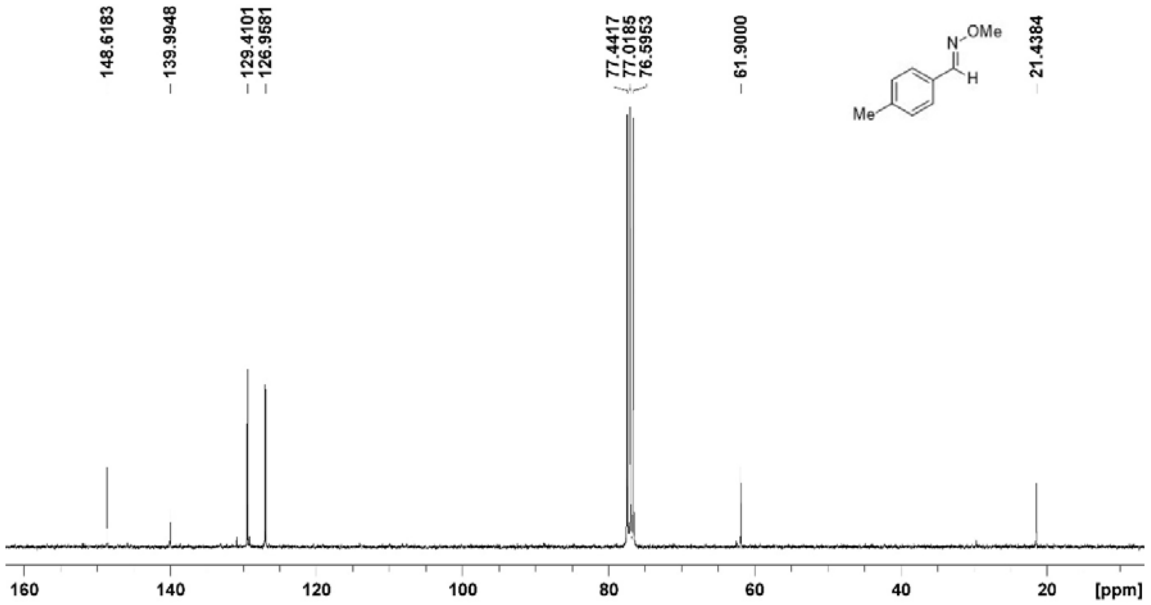

Figure S33. ^{13}C NMR (75 MHz) spectrum of compound **2m**.

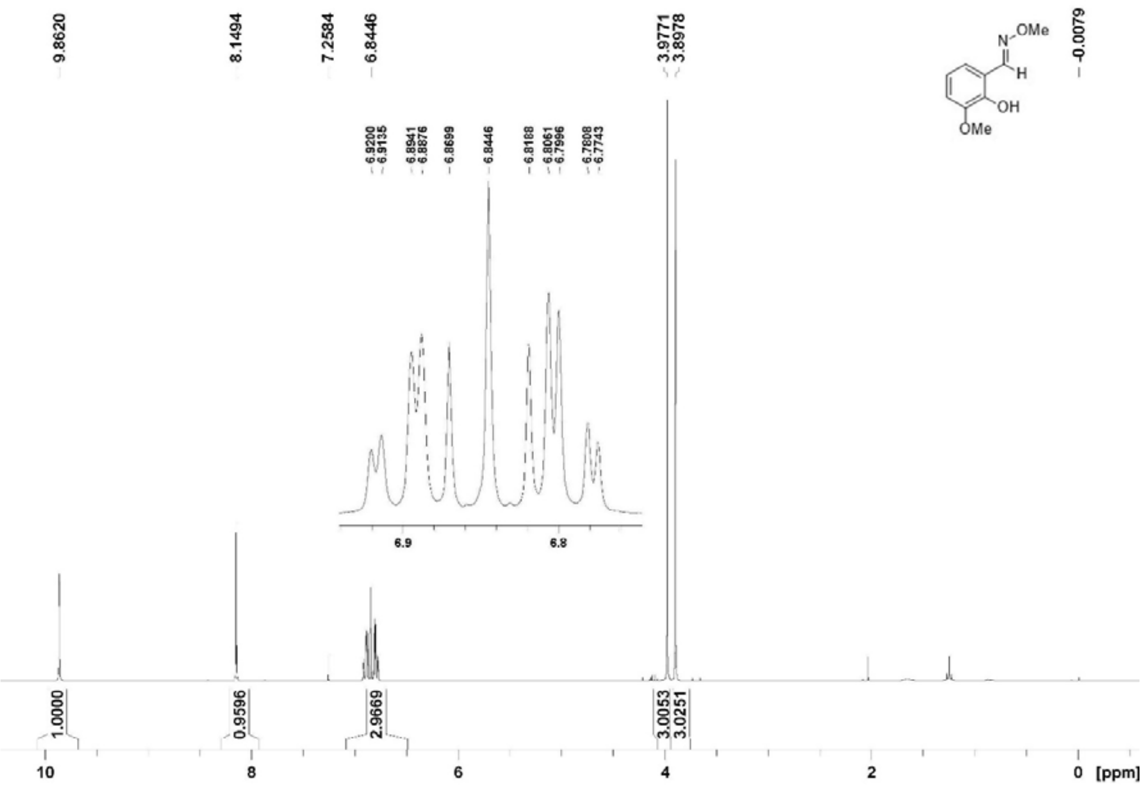

Figure S34. ^{1}H NMR (300 MHz) spectrum of compound **2n**.

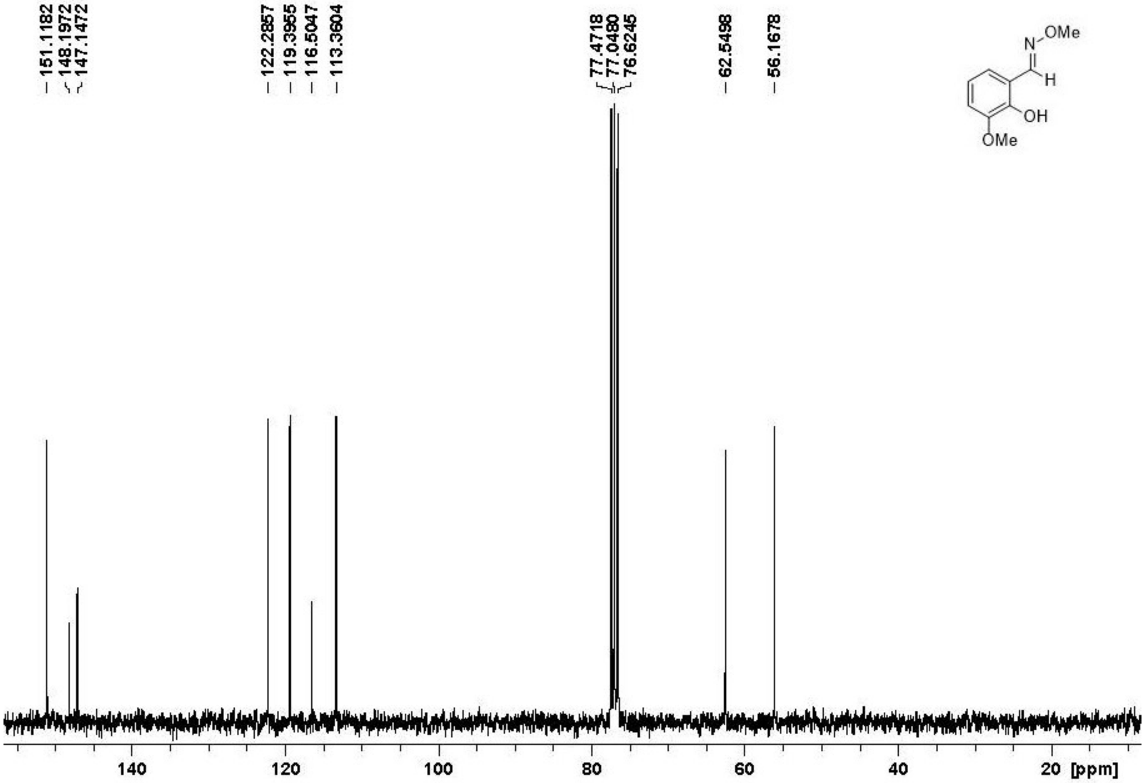

Figure S35. ^{13}C NMR (75 MHz) spectrum of compound **2n**.

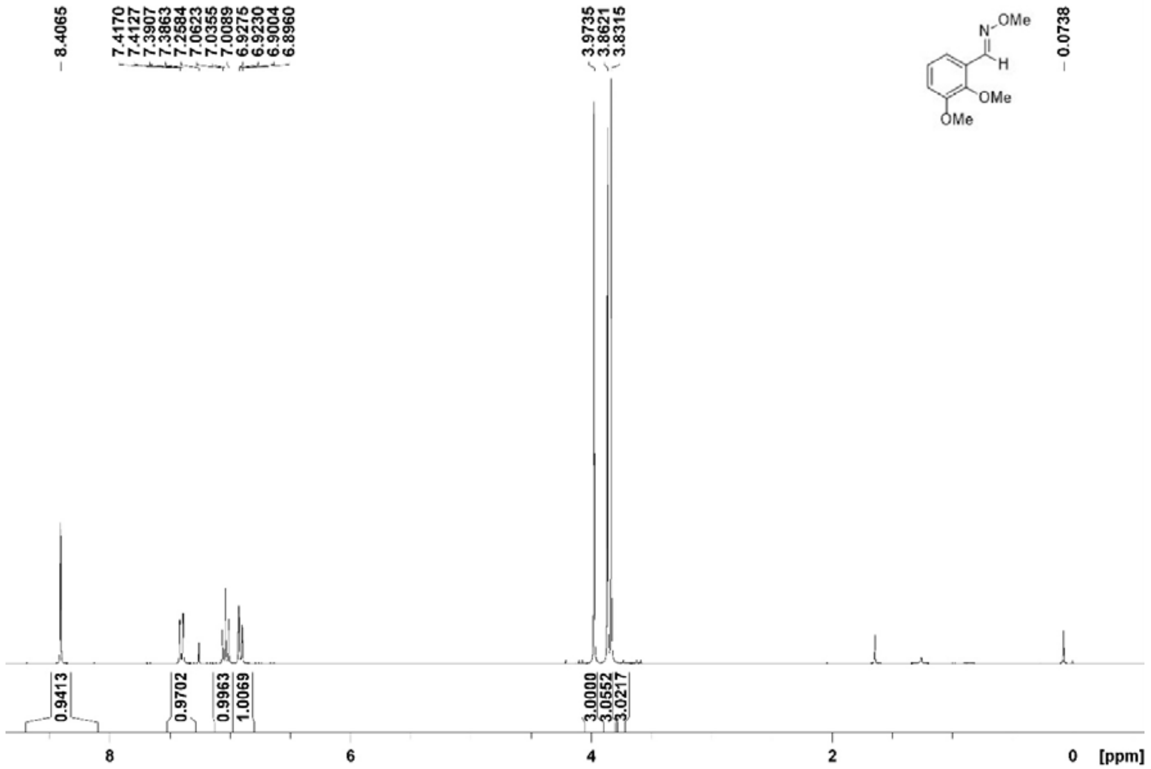

Figure S36. ^{1}H NMR (300 MHz) spectrum of compound **2o**.

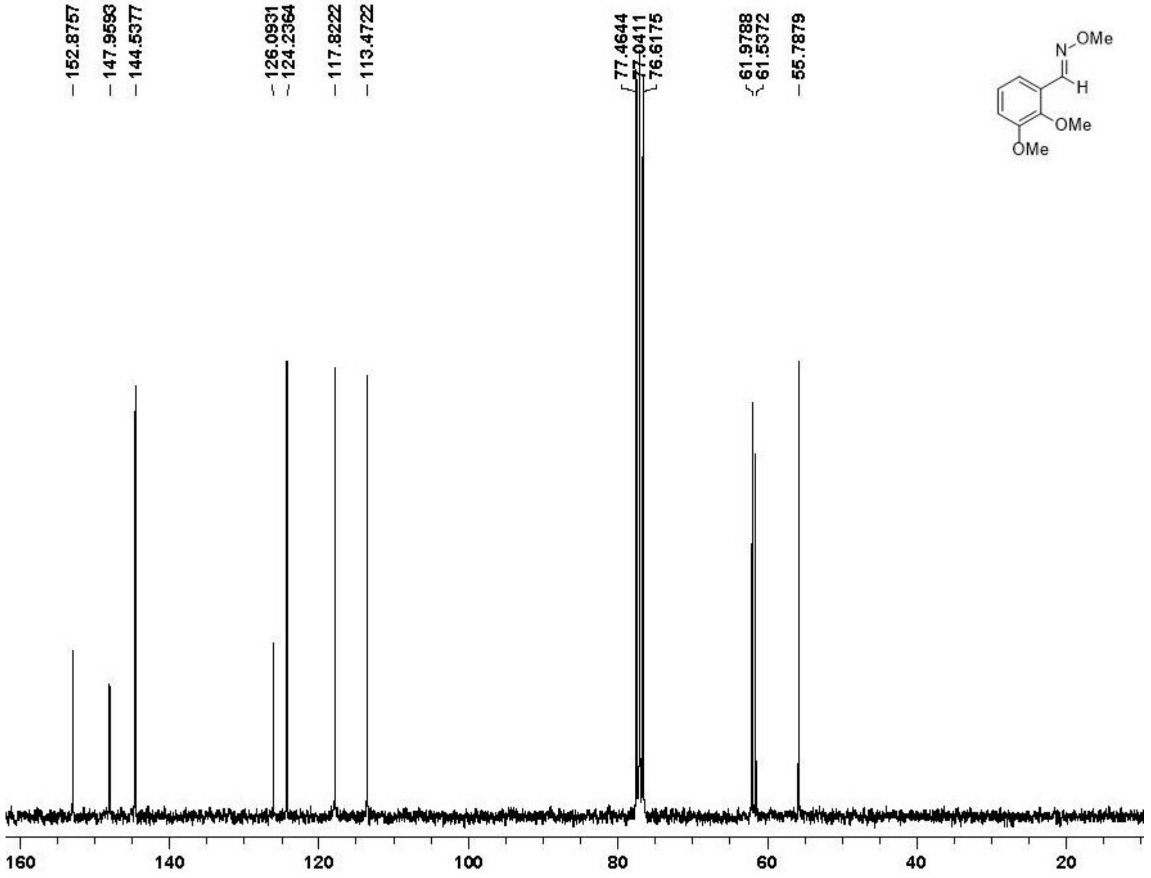

Figure S37. ^{13}C NMR (75 MHz) spectrum of compound **2o**.

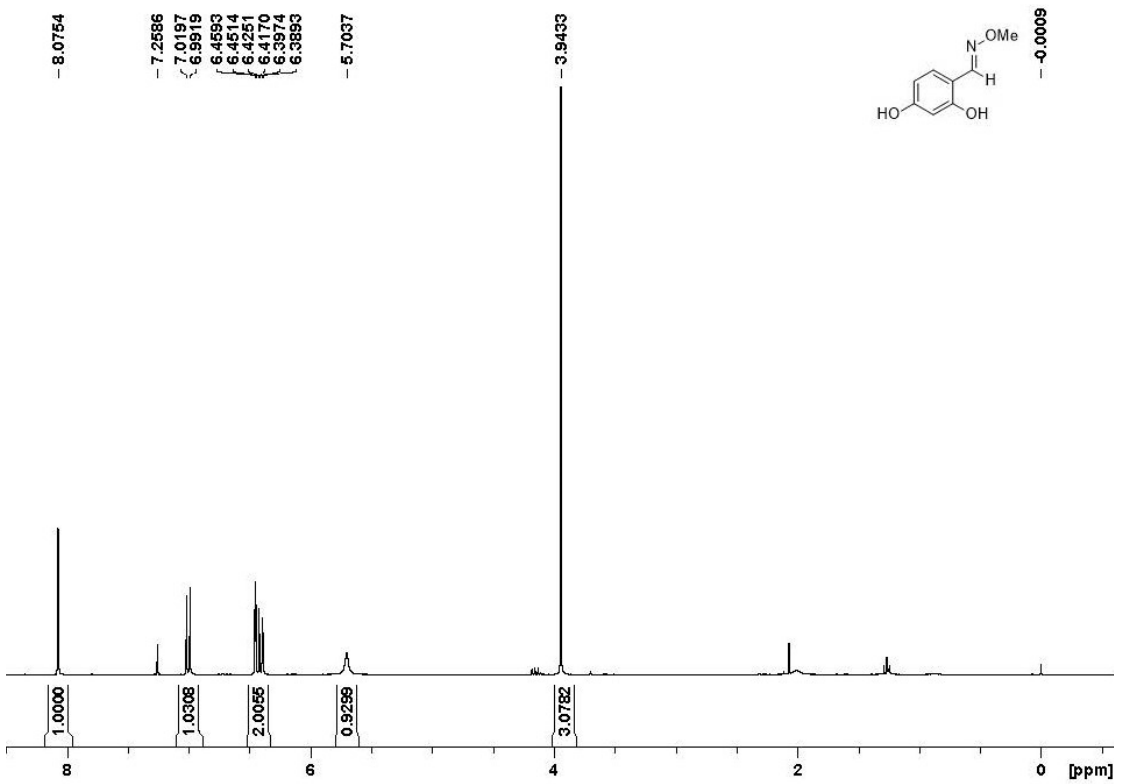

Figure S38. ^{1}H NMR (300 MHz) spectrum of compound **2p**.

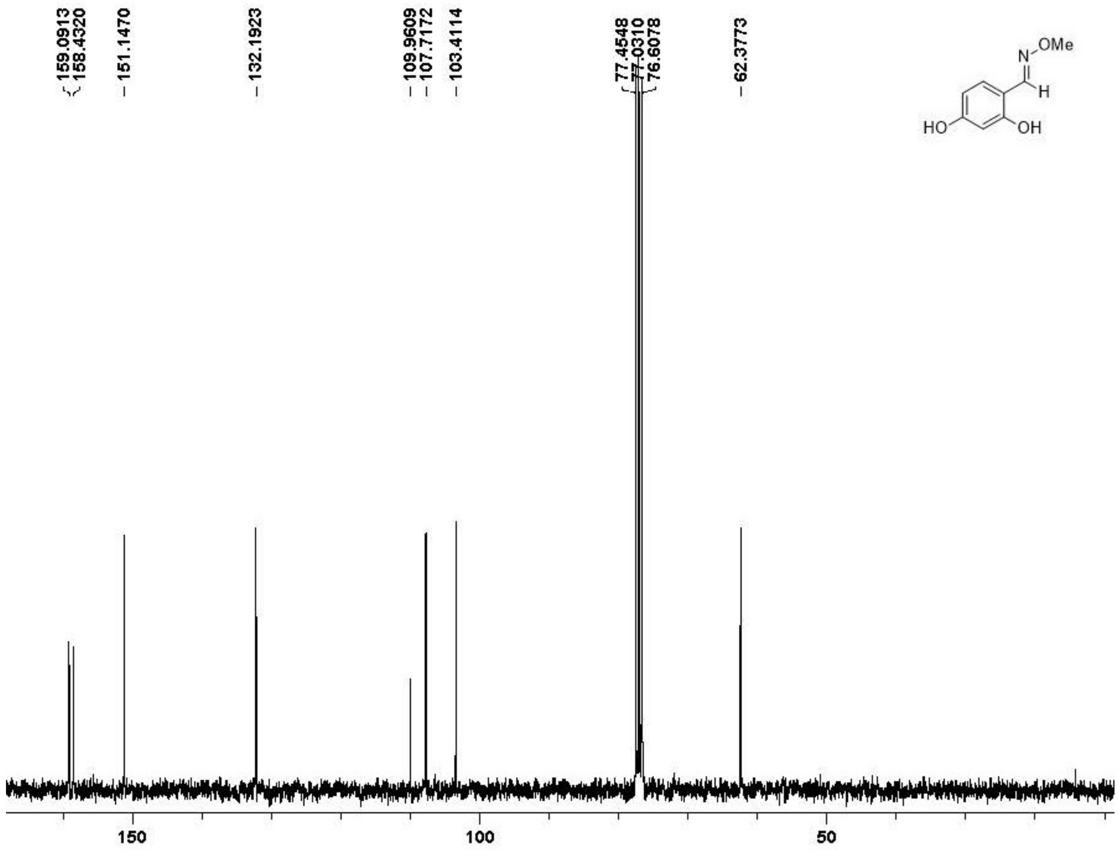

Figure S39. ^{13}C NMR (75 MHz) spectrum of compound **2p**.

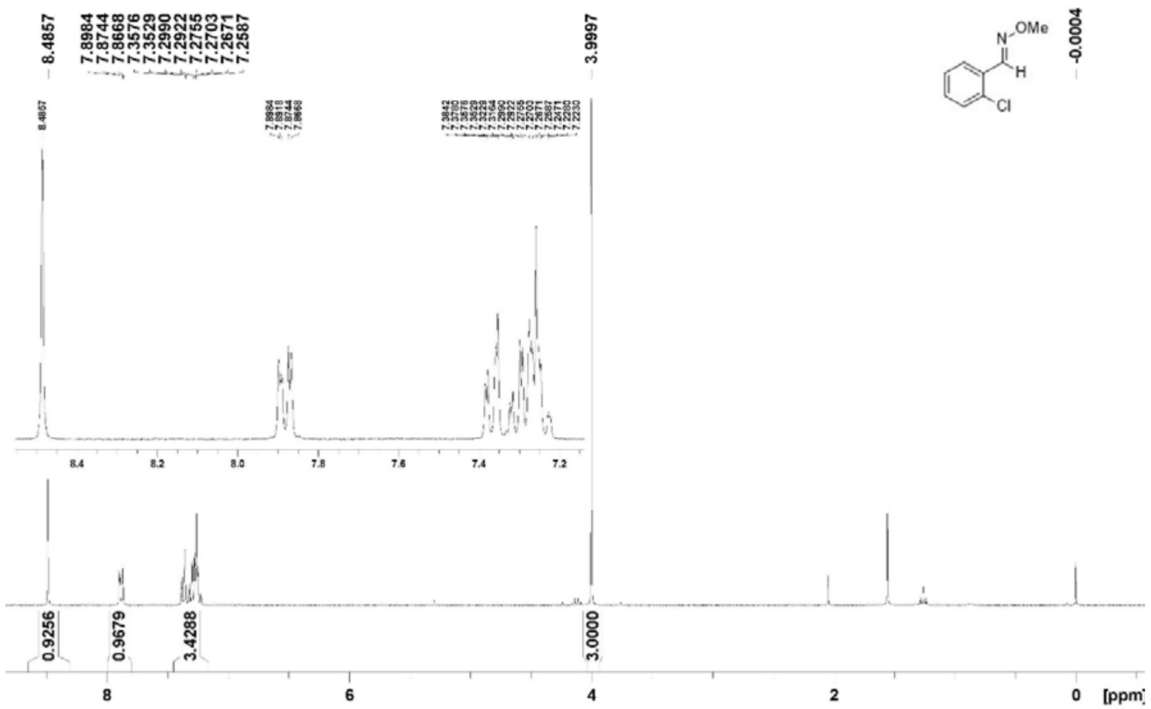

Figure S40. ^{1}H NMR (300 MHz) spectrum of compound **2q**.

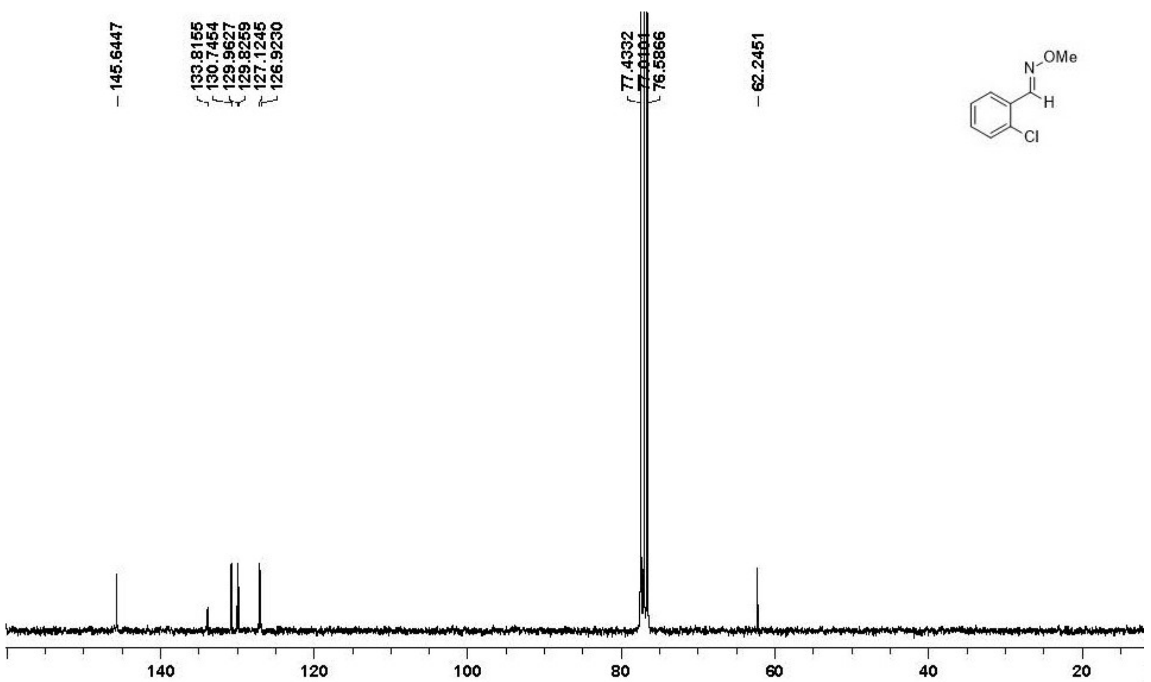

Figure S41. ^{13}C NMR (75 MHz) spectrum of compound **2q**.

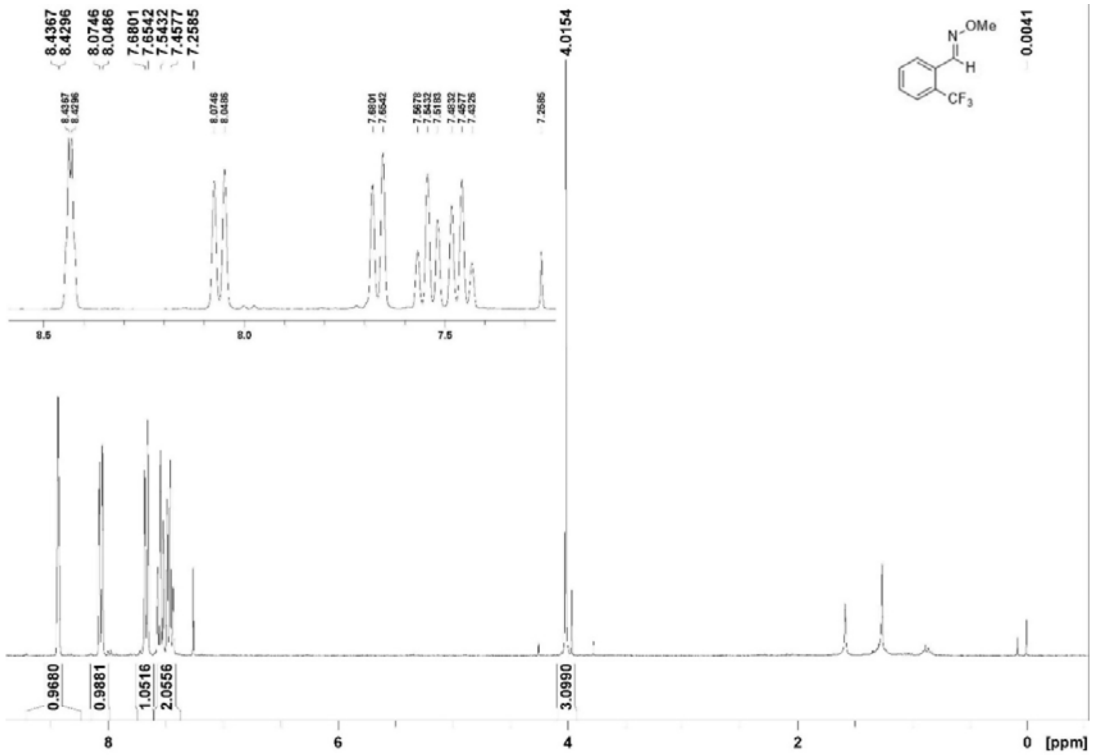

Figure S42. ^{1}H NMR (300 MHz) spectrum of compound **2r**.

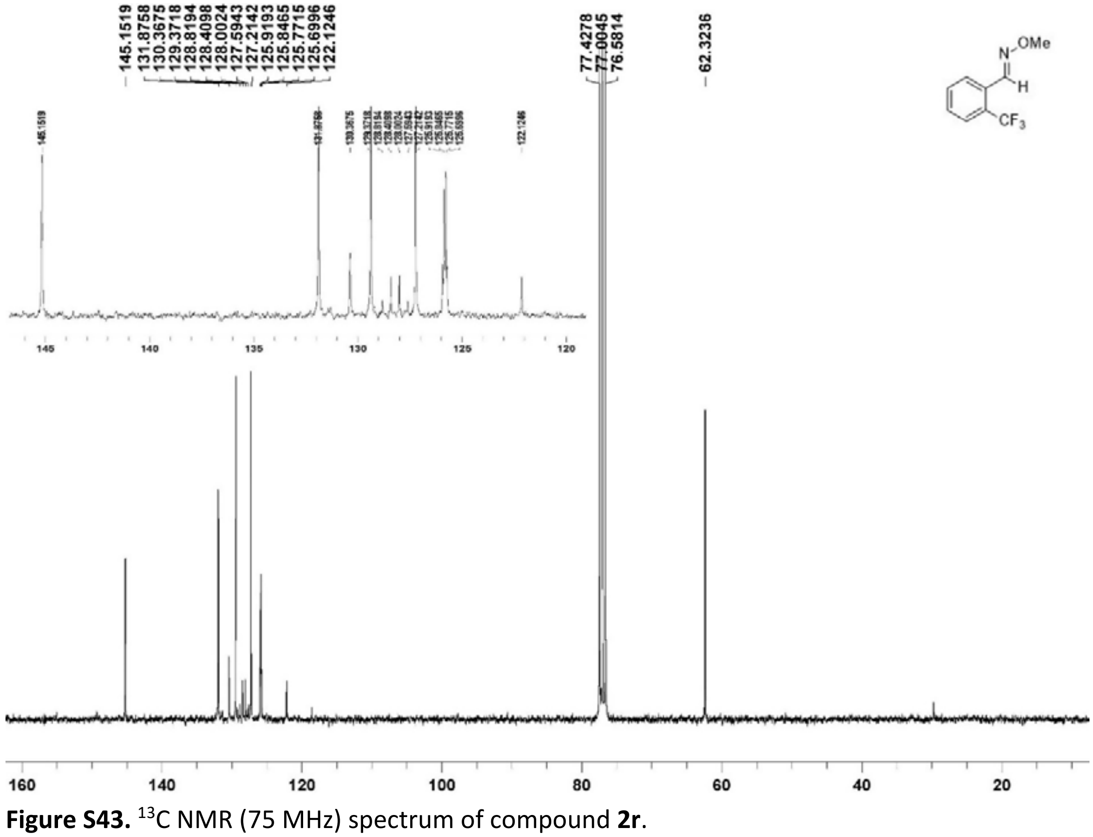

Figure S43. ^{13}C NMR (75 MHz) spectrum of compound **2r**.

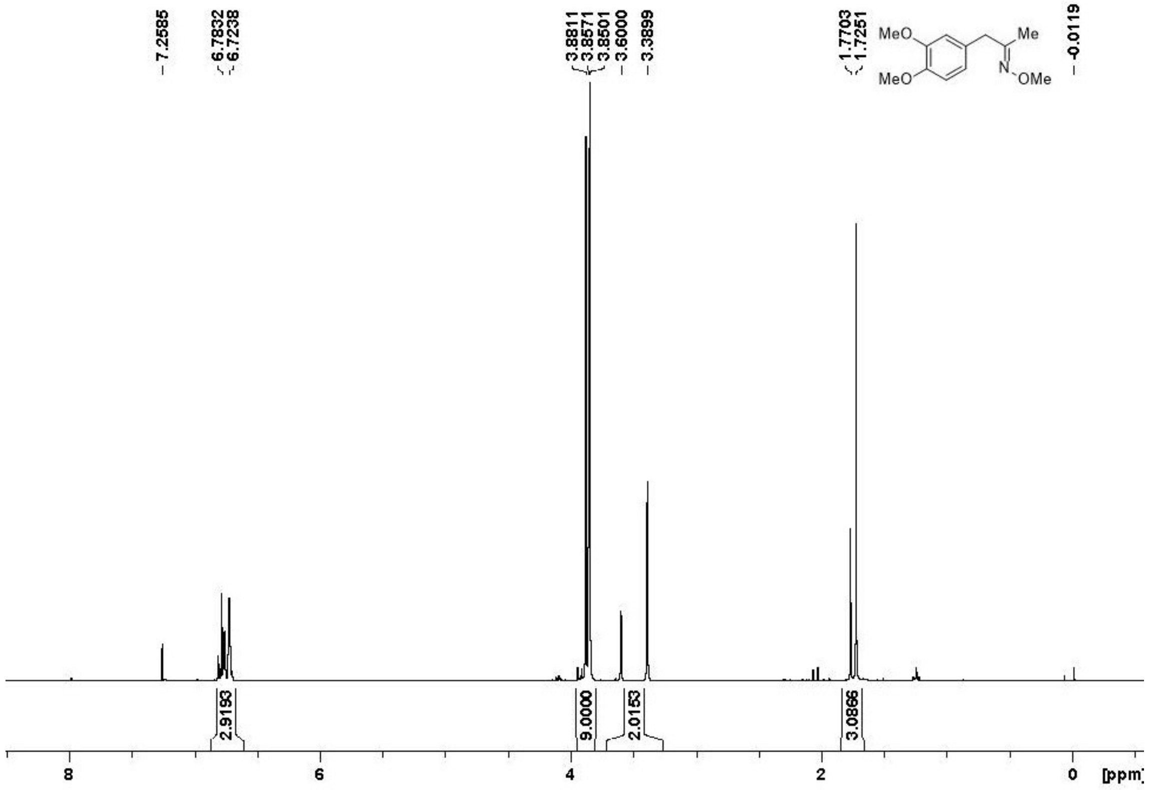

Figure S44. ^{1}H NMR (300 MHz) spectrum of compound **2s**.

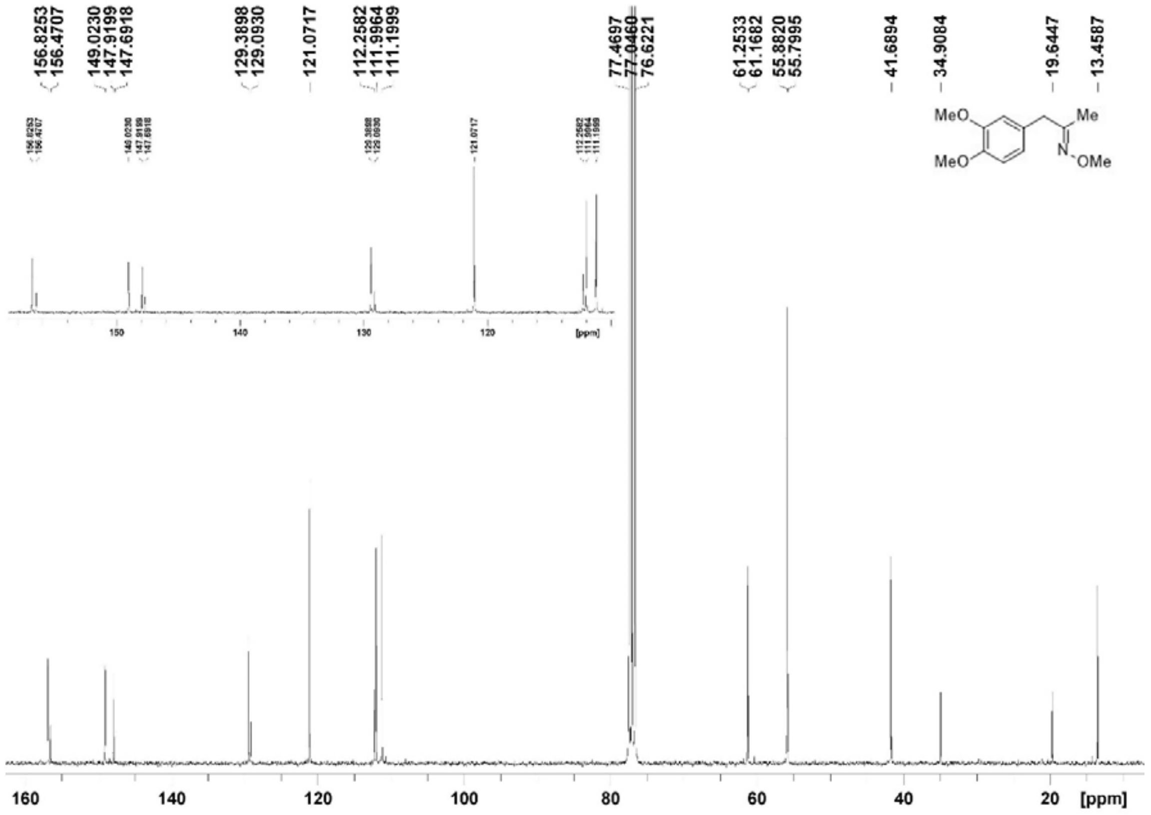

Figure S45. ^{13}C NMR (75 MHz) spectrum of compound **2s**.

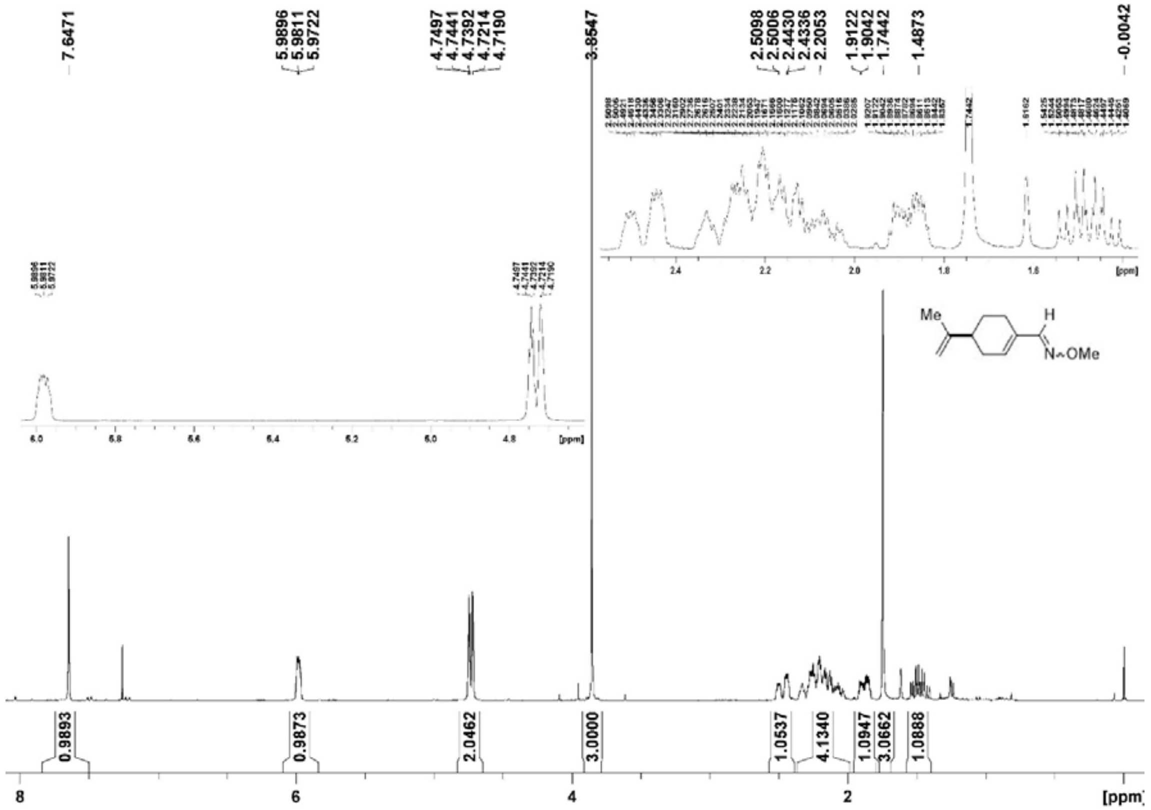

Figure S46. ^{1}H NMR (300 MHz) spectrum of compound **2t**.

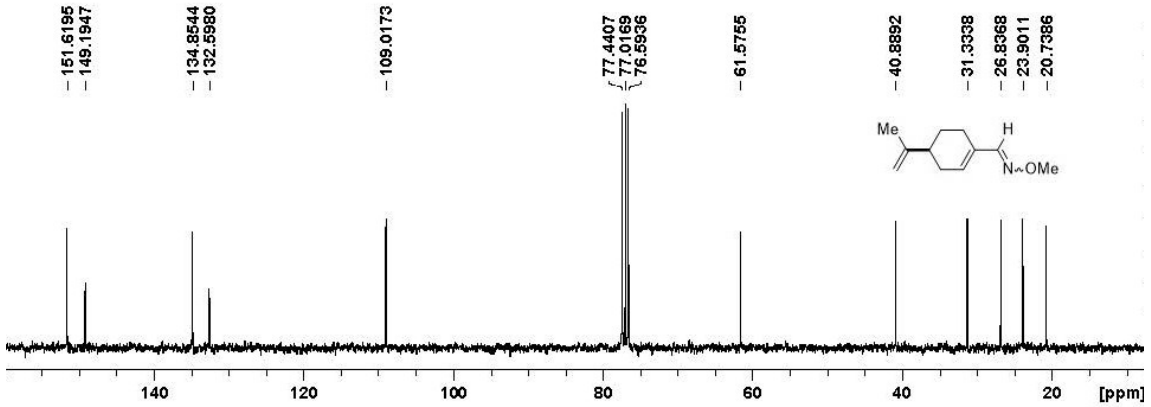

Figure S47. ^{13}C NMR (75 MHz) spectrum of compound **2t**.

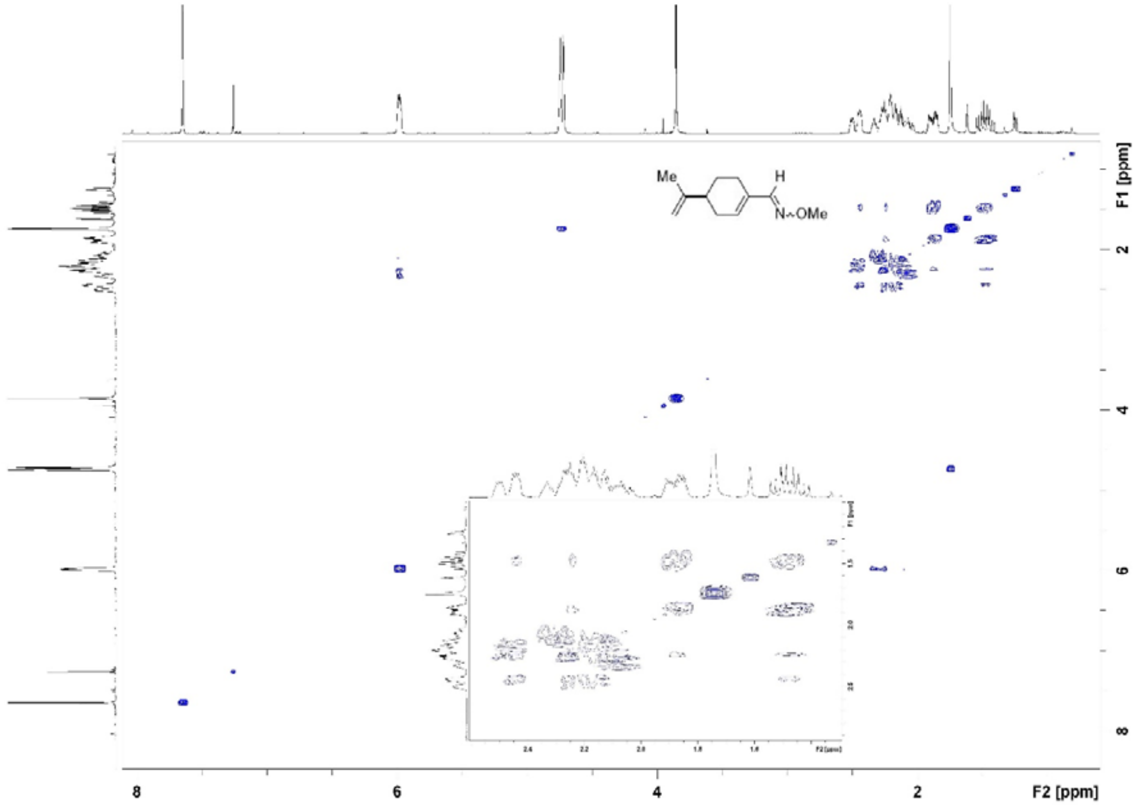

Figure S48. COSY spectrum of compound **2t**.

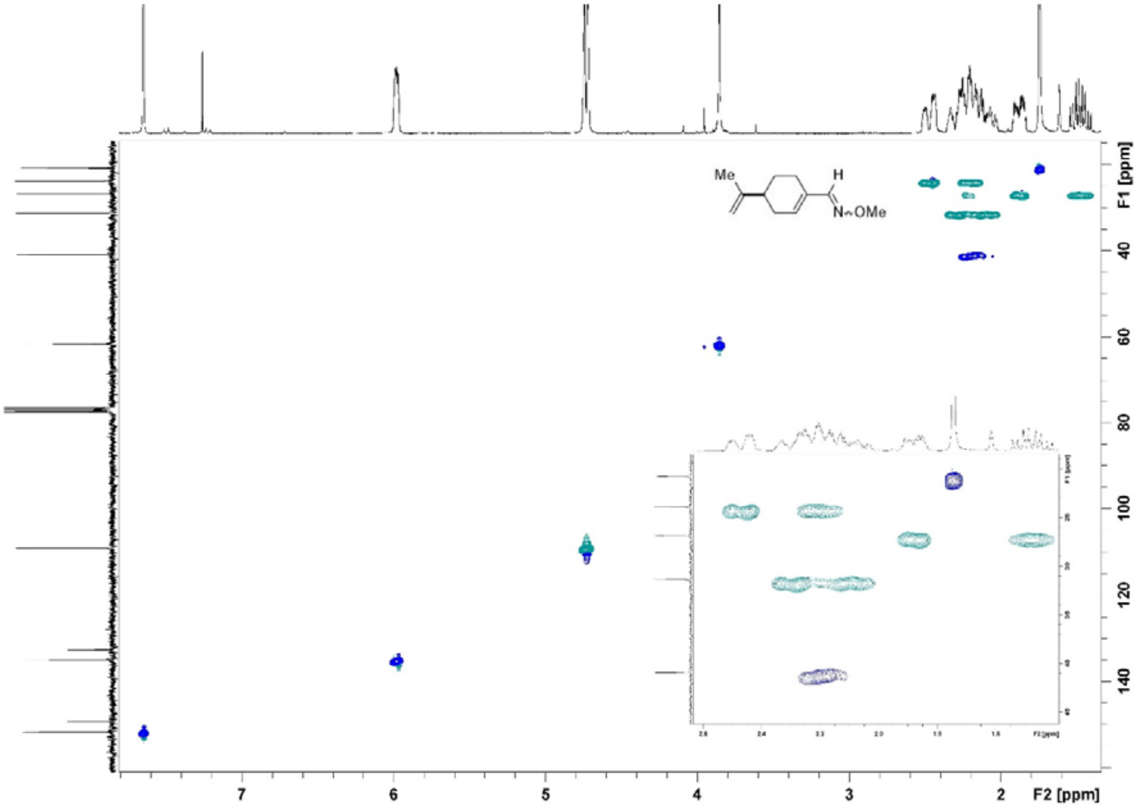

Figure S49. HSQC spectrum of compound **2t**.

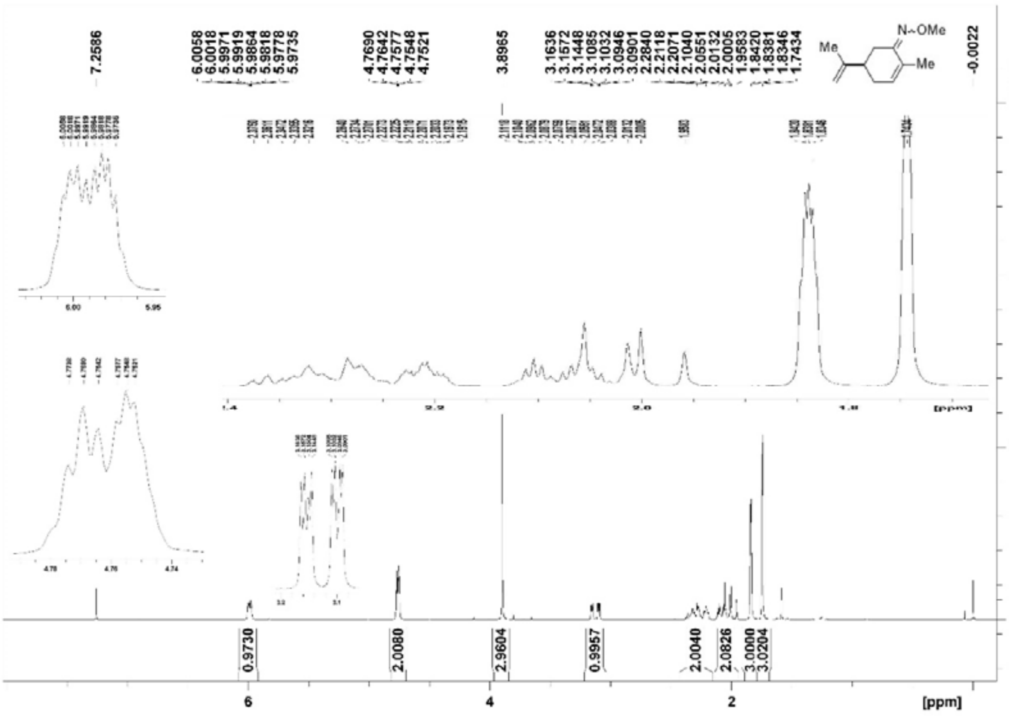

Figure S50. ^{1}H NMR (300 MHz) spectrum of compound **2u**.

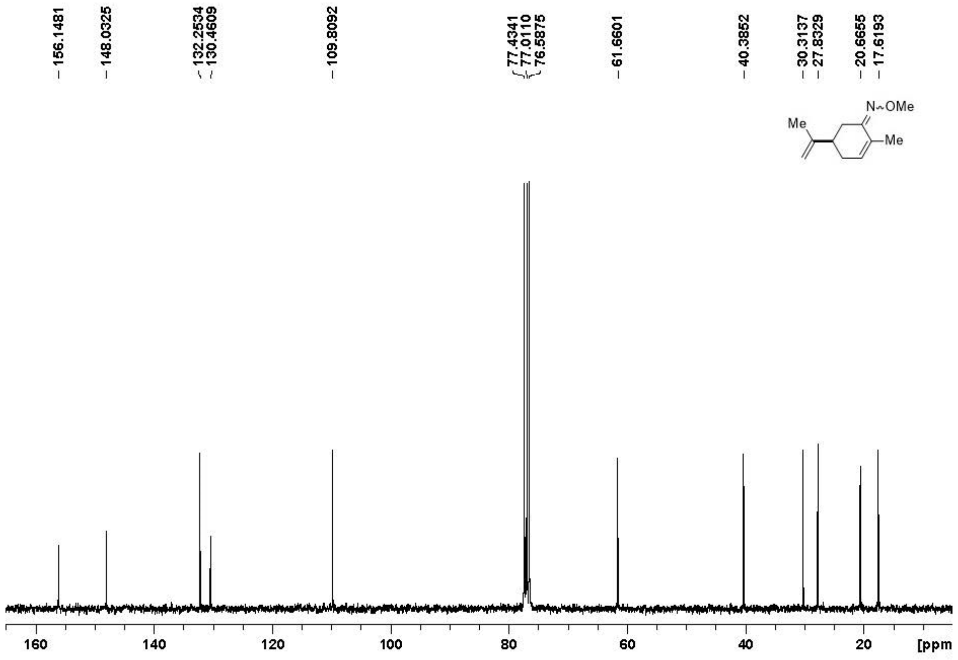

Figure S51. ^{13}C NMR (75 MHz) spectrum of compound **2u**.

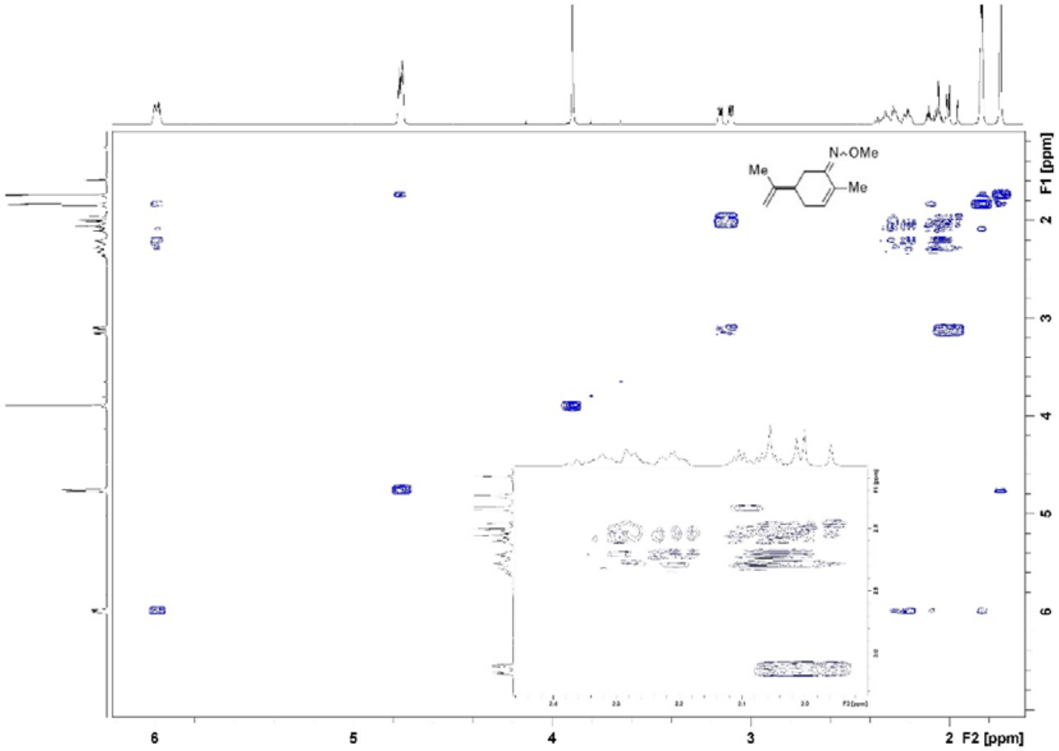

Figure S52. COSY spectrum of compound **2u**.

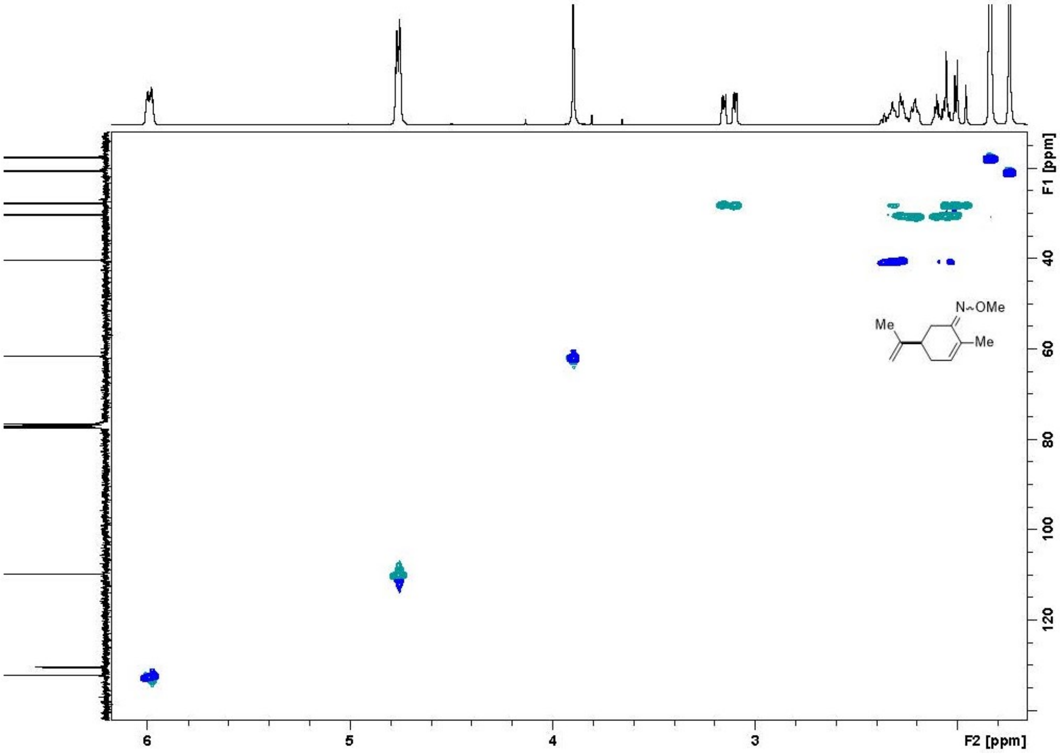

Figure S53. HSQC spectrum of compound **2u**.

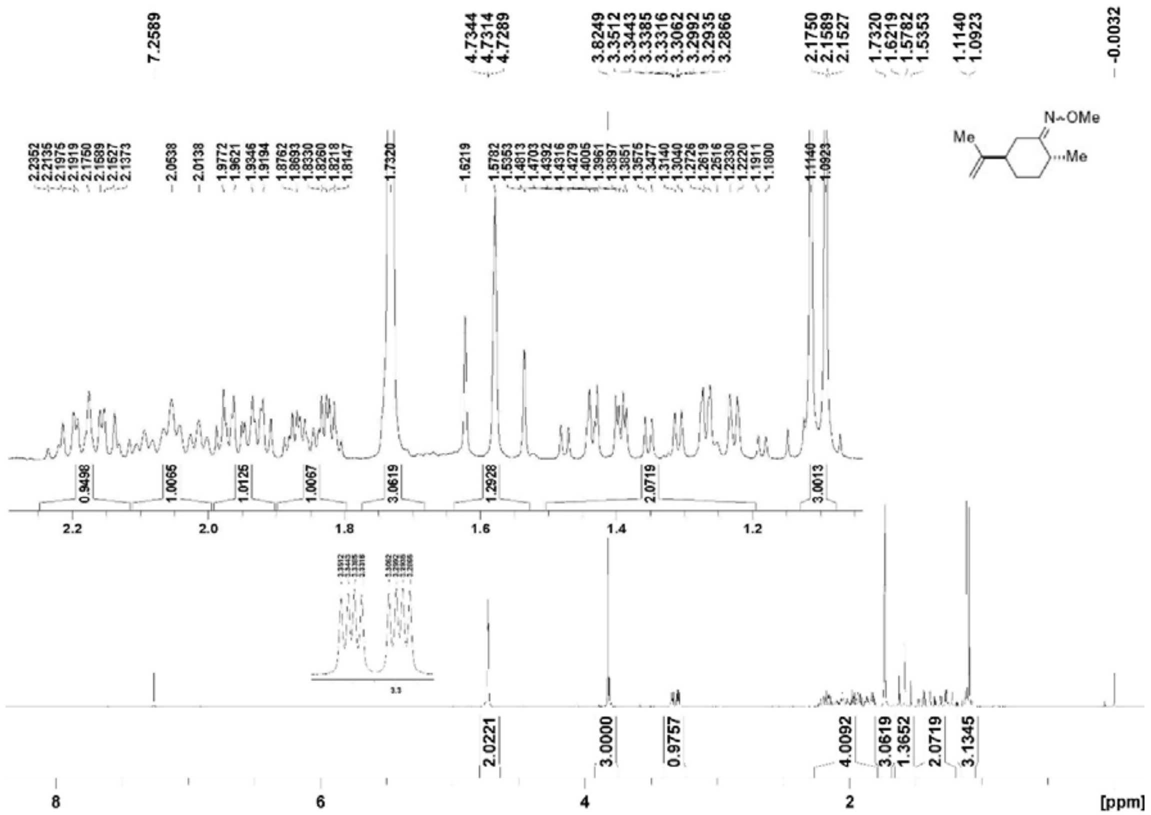

Figure S54. ^{1}H NMR (300 MHz) spectrum of compound **2v**.

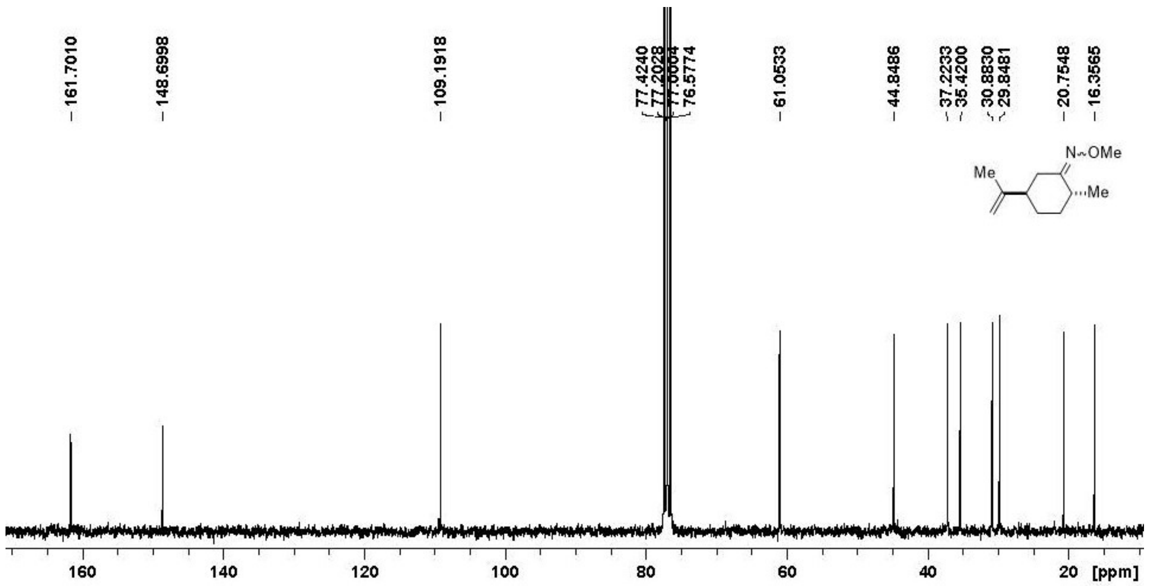

Figure S55. ^{13}C NMR (75 MHz) spectrum of compound **2v**.

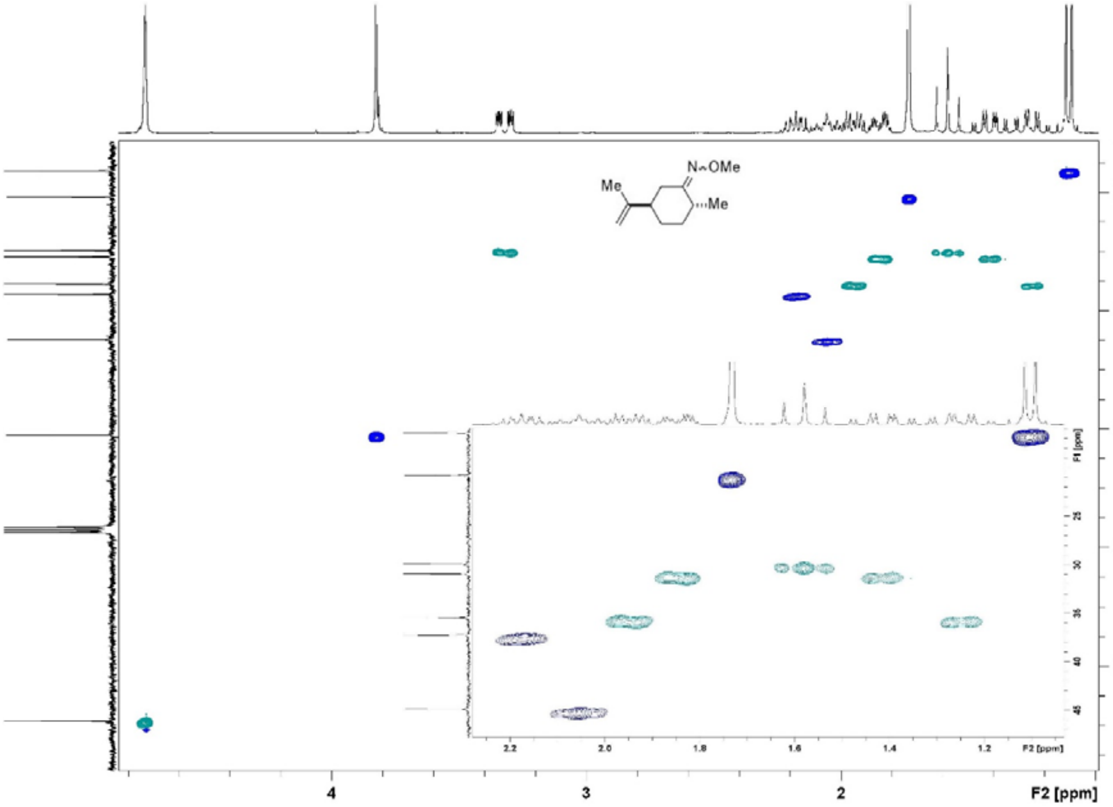

Figure S56. HSQC spectrum of compound **2v**.

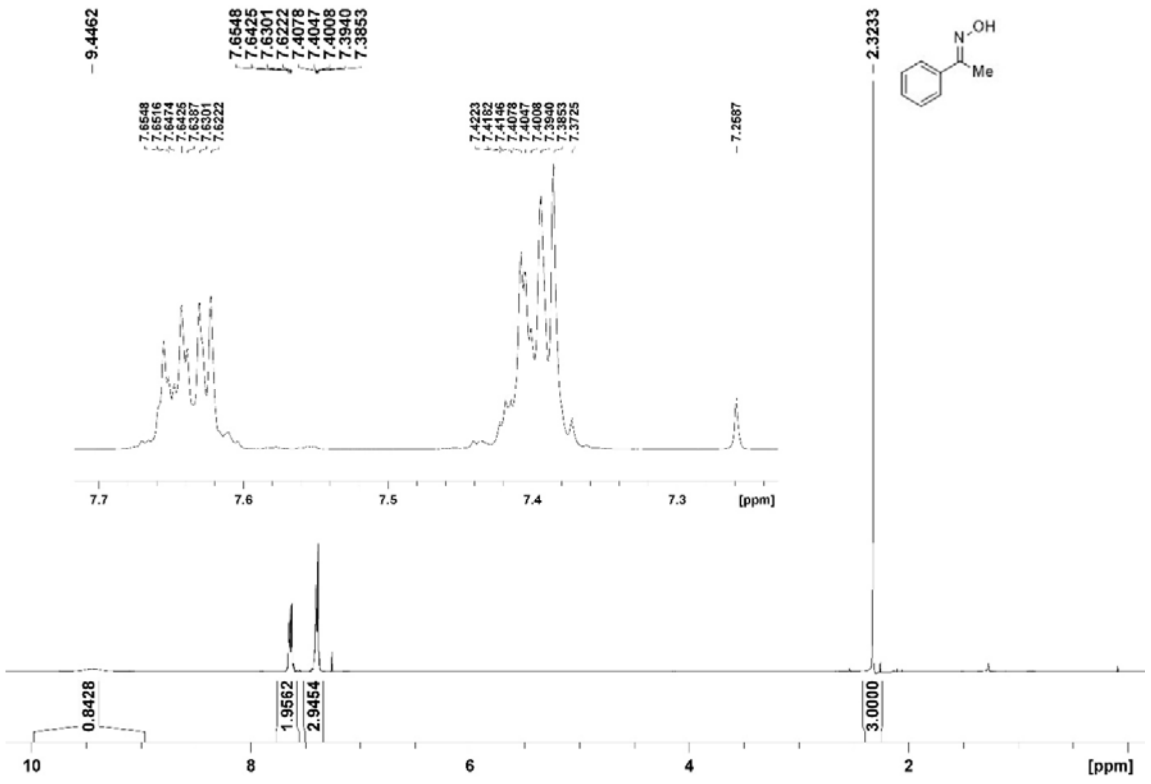

Figure S57. ^{1}H NMR (300 MHz) spectrum of compound **2w**.

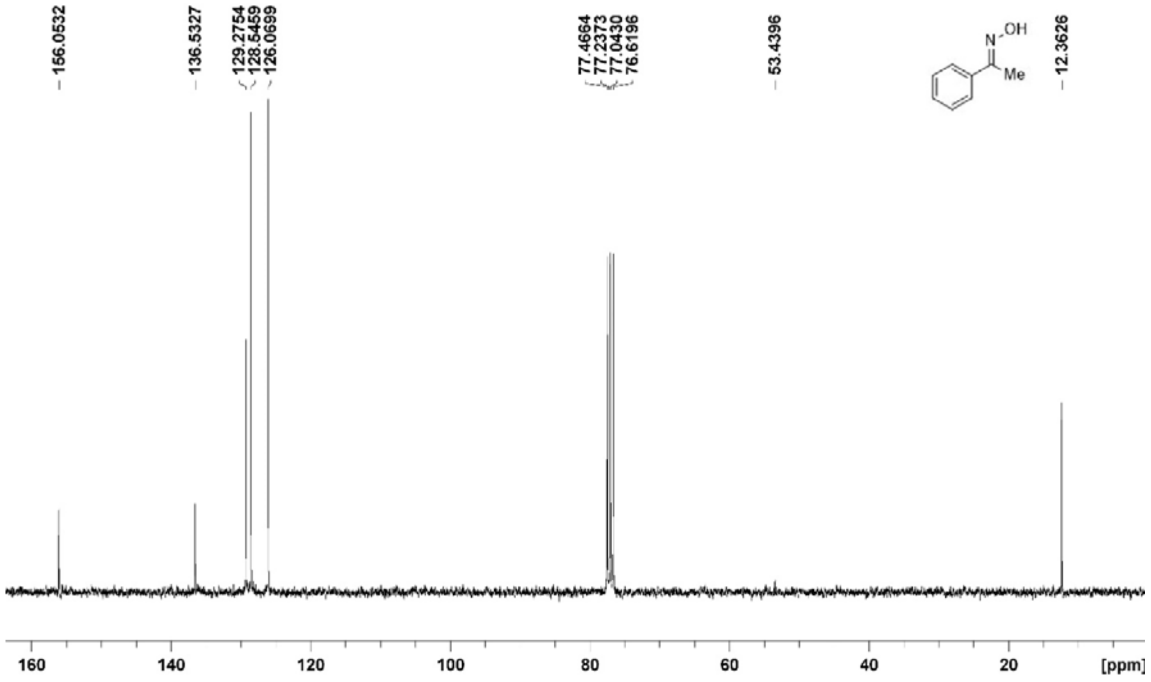

Figure S58. ^{13}C NMR (75 MHz) spectrum of compound **2w**.

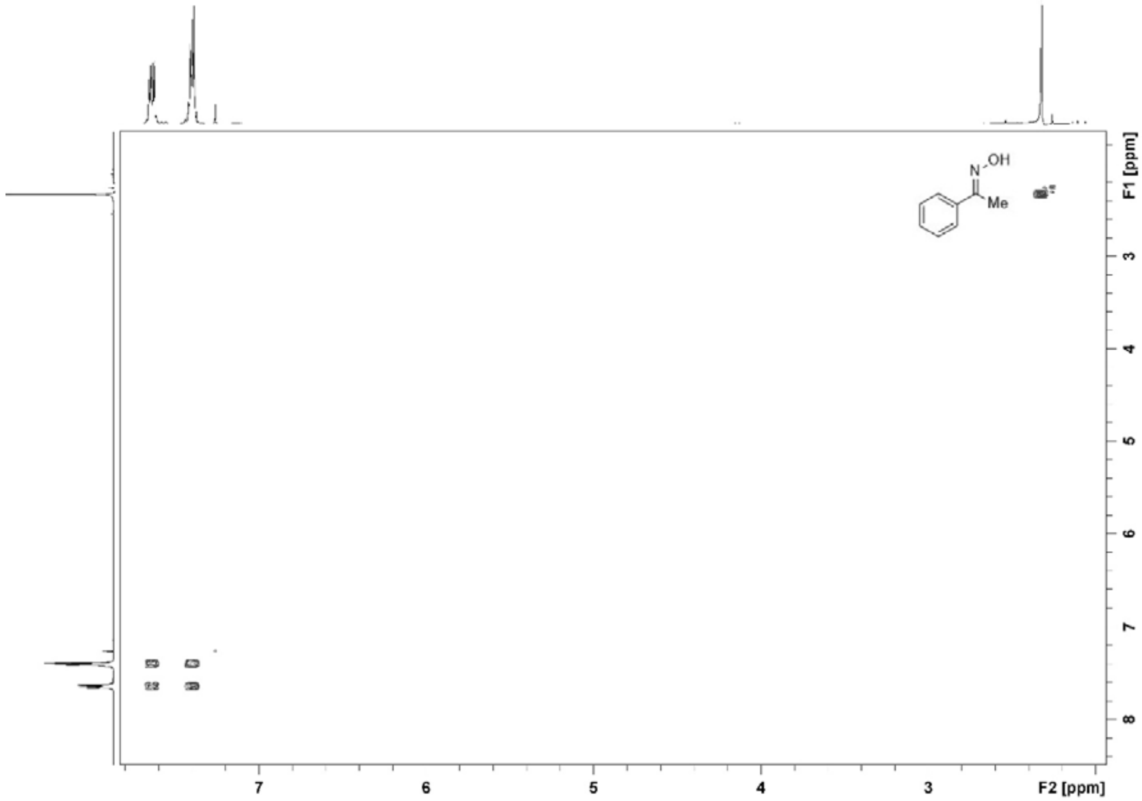

Figure S59. COSY spectrum of compound **2w**.

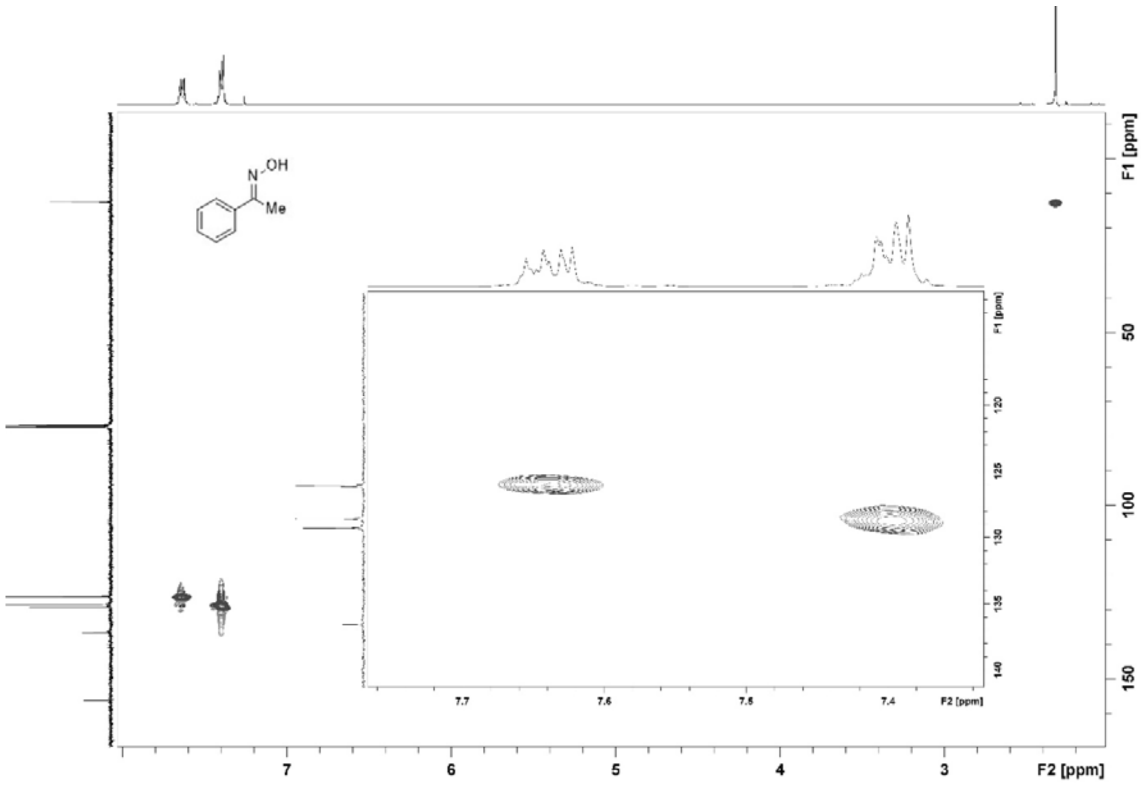

Figure S60. HSQC spectrum of compound **2w**.
