# Supplementary material for: A convenient and eco-friendly cerium(III) chloride-catalysed synthesis of methoxime derivatives of aromatic aldehydes and ketones
Source: R Soc Open Sci. 2018 May 23;5(5):180279. doi: 10.1098/rsos.180279 (PMC5990813; doi:10.1098/rsos.180279)
Supplement: ESM - Literature survey of conditions and yields of other methods [file rsos180279supp2.pdf]

Electronic Supplementary Material (ESI)
for Royal Society Open Science

Supplementary Material for

A convenient and eco-friendly cerium(III) chloride-catalyzed synthesis of methoxime derivatives of aromatic aldehydes and ketones

Iván Cortés, Teodoro S. Kaufman* and Andrea B. J. Bracca*

Instituto de Química Rosario (IQUIR, CONICET-UNR) and Facultad de Ciencias Bioquímicas y Farmacéuticas, Universidad Nacional de Rosario, Suipacha 531, S2002LRK Rosario, Argentina

E-mail: kaufman@iquir-conicet.gov.ar; bracca@iquir-conicet.gov.ar

TABLE OF CONTENTS

Table S1. Literature survey of conditions and yields of previous syntheses of the methoximes S2

Table S1. Literature survey of conditions and yields of previous syntheses of the methoximes

Entry N°	Product	$MeONH_2$•HCl (equiv)	Base (equiv.)	Solvent (*v/v*)	Temp. (°C)	Time (h)	Yield (%)	Ref.
**1**	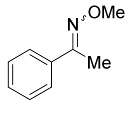	2.7	NaOAc (4.4)	H_2O/EtOH (3:1)	70	2	87	1
		1.2	Pyridine (excess)	MeOH:Pyridine (6.67:1)	Reflux	ON	86	2
		1.0	-	MeOH	rt	Several	72	3
**2**	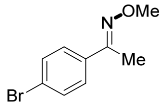	4.7	NaOAc (4.0)	H_2O/MeOH (2.5:1)	80	12	99	4
		2.7	NaOAc (4.4)	H_2O/EtOH (3:1)	70	2	87	1
		1.2	Pyridine (excess)	Pyridine (solvent)	Reflux	ON	86	5
		3.0	$NaHCO_3$ (3.0)	MeOH	rt	4	ND	6
**3**	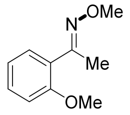	2.7	NaOAc (4.4)	H_2O/EtOH (3:1)	70	2	ND	7
**4**	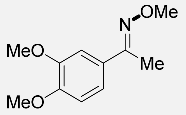							New
**5**	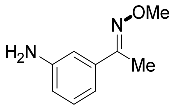	1.0	No	EtOH	55	13	88	8
**6**	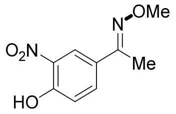	1	HCl (0.6)	EtOH	rt	4	ND	9
**7**	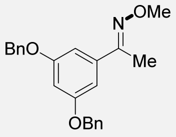							New
**8**	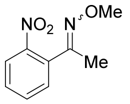	1.0	Pyridine (excess)	EtOH/Pyridine (1:1)	Reflux	3	87	10
		1.0	Pyridine (excess)	EtOH/Pyridine (1:1)	Reflux	ON	ND	11
**9**	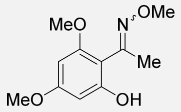							New
**10**	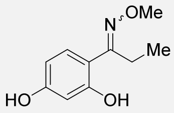							New

**11**	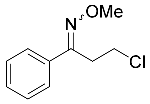	1.5	NaOAc (1.5)	Dioxane/MeOH/H_2O (20:5:7)	rt	24	ND	12
**12**	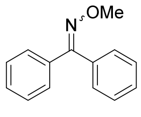	1.6	Pyridine (excess)	EtOH/Pyridine (8:1)	50	24	95	13
		2.0	NaOAc (4.0)	H_2O/EtOH (3:1)	Reflux	3	87	14
		1.0	-	EtOH	Reflux	20	91	15
**13**	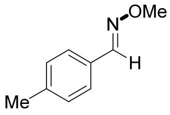	1.7	NaOAc (1.4)	H_2O/THF (3:1)	rt	4	95	16
		ND	NaOAc	EtOH	ND	ND	90	17
		1.4	Pyridine (2.2)	CH_2Cl_2	rt	ON	ND	18
**14**	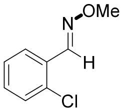	1.1	NaOH (1.1)	H_2O/EtOH (1:4)	45	1.5	60	19
		1.2	Pyridine (4.0)	CH_2Cl_2	rt	1	ND	20
**15**	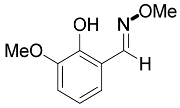	3	NaOAc (9.0)	H_2O/EtOH (5:8)	Reflux	1.5	84	21
**16**	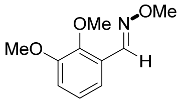	ND	ND	EtOH	Reflux	3-6	ND	22
		1.2	Pyridine (4.0)	CH_2Cl_2	rt	1	ND	16,20
**17**	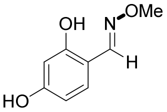	3.5	NaOAc (10)	H_2O/EtOH (2.5:1)	Reflux	1.5	~90	17,21
**18**	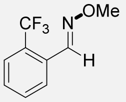							New
**19**	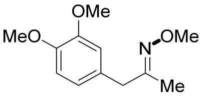	2	NaOAc (2.0)	H_2O/EtOH (1:4)	rt	ND	78	23
		1.5	Na_2CO_3 (0.76)	H_2O	rt	14	91	24
**20**	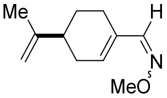	1.3	Et_3N (1.3)	EtOH	rt	ON	84	25
**21**	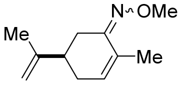	2.7	NaOAc (4.4)	Pyridine	Reflux	ON	ND	26
**22**	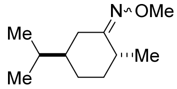	2.7	NaOAc (4.4)	Pyridine	Reflux	ON	ND	20,26

ND= The information was not disclosed; ON= Overnight; rt= Room temperature.

References

1. Tsai AS, Brasse M, Bergman RG, Ellman JA. 2011 Rh(III)-catalyzed oxidative coupling of unactivated alkenes via C−H activation. *Org. Lett.* **13**, 540–542. (doi: 10.1021/ol102890k)

2. Beak P, Basha A, Kokko B, Loo D. 1986 The geometry of displacements at nonstereogenic atoms: The formal displacement of alkoxide from alkoxyamines by organolithium reagents. *J. Am. Chem. Soc.* **108**, 6016–6023. (doi: 10.1021/ja00279a058)
3. Mohr J, Oestreich M. 2014 $B(C_6F_5)_3$-Catalyzed hydrogenation of oxime ethers without cleavage of the *N–O* bond. *Angew. Chem. Int. Ed.* **53**, 13278–13281. (doi: 10.1002/anie.201407324)
4. Park Y, Jee S, Kim JG, Chang S. 2015 Study of sustainability and scalability in the Cp*Rh(III)-catalyzed direct C–H amidation with 1,4,2-dioxazol-5-ones. *Org. Proc. Res. Dev.* **19**, 1024–1029. (doi:10.1021/acs.oprd.5b00164)
5. Liu Y-K, Lou S-J, Xu D-Q, Xu Z-Y. 2010 Regiospecific synthesis of nitroarenes by palladium-catalyzed nitrogen-donor-directed aromatic C–H nitration. *Chem. Eur. J.* **16**, 13590–13593. (doi:10.1002/chem.201002581)
6. Zou M, Liu J, Tang C, Jiao N. 2016 Rh-catalyzed *N–O* bond cleavage of anthranil: A C–H amination reagent for simultaneous incorporation of amine and a functional group. *Org. Lett.* **18**, 3030–3033 (doi: 10.1021/acs.orglett.6b01459)
7. Li Z-Y, Wang G-W. 2015 Palladium-catalyzed decarboxylative *ortho*-ethoxycarbonylation of *O*-methyl ketoximes and 2-arylpyridines with potassium oxalate monoester. *Org. Lett.* **17**, 4866–4869. (doi.org/10.1021/acs.orglett.5b02422)
8. Ren Z-L, Yang C-J, Lu R-E, Li X-R. 2014 Synthesis and crystal structures of 1-(3-{[(*E*)-3,5-dibromo-2-hydroxybenzylidene]amino}phenyl)ethanone *O*-methyloxime. *Asian J. Chem*. **26**, 5113–5115. (doi:10.14233/ajchem.2014.16410)
9. Shioda T, Arimori S, 2015 Patent EP 2940012.
10. Vicker N, Bailey H, Heaton W, Day JM, Purohit A, Potter BVL. 2009 Patent WO 66072.
11. Smith II LM, Pinto DJP, Corte JR, Ewing WR. 2016 Patent WO 205482
12. Kolasa T, Patel M, Mortell KH, Matulenko MA, Hakeem AA, Bhatia PA, Wang X, Daanen JF, Latshaw SP, Stewart AO. 2005 Patent US 2005/176727.
13. Pilgrim BS, Gatland AE, Esteves CHA, McTernan CT, Jones GR, Tatton MR, Procopiou PA, Donohoe TJ. 2016 Palladium-catalyzed enolate arylation as a key C–C bond-forming reaction for the synthesis of isoquinolines. *Org. Biomol. Chem*. **14**, 1065–1090. (doi:10.1039/C5OB02320C)
14. Xu D, Lou S, Xu ZY. 2016 Patent CN 103922904.
15. Matsushita K, Okamoto C, Yoshimoto M, Harada K, Kubo M, Fukuyama Y, Hioki H. 2010 Novel alkoxyamine linker to load ketones for solid-phase synthesis: Application of the synthesis of 1,4-benzodiazepine-2-ones. *J. Comb. Chem*. **12**, 311–314. (doi:10.1021/cc9001795)
16. Walker MA, Banville J, Remillard R, Plamondon S. 2003 Patent US 176495.
17. Konakahara T, Matsuki M, Sugimoto S, Sato K. 1987 Stereoselective synthesis of trans-2-aryl-3-(2-pyridyl) aziridines from an α-silyl carbanion. *J. Chem. Soc. Perkin Trans. 1*. 1489–1493. (doi:10.1039/P19870001489)
18. Langenhan JM, Endo MM, Engle JM, Fukumoto LL, Rogalsky DR, Slevin LK, Fay LR, Lucker RW, Rohlfing JR, Smith KR, et al. 2011 Synthesis and biological evaluation of RON-neoglycosides as tumor cytotoxins. *Carbohydr. Res.* **346**, 2663–2676. (doi: 10.1016/j.carres.2011.09.019)
19. Chang JH, 1983 Patent US 4405357.
20. Dubost E, Fossey C, Cailly T, Rault S, Fabis F. 2011 Selective *ortho*-bromination of substituted benzaldoximes using Pd-catalyzed C–H activation: Application to the synthesis of substituted 2-bromobenzaldehydes. *J. Org. Chem.* **76**, 6414–6420. (doi: 10.1021/jo200853j)
21. Huffman MN, 1953 Patent US 2712031

22. Sun C-L, Liu J, Wang Y, Zhou X, Li B-J, Shi Z-J. 2011 Direct sequential C-O and C-C formation via double sp^2 C-H bond activations to construct 6*H*-benzo[*c*]chromen-6-ones. *Synlett*, 883–886. (doi: 10.1055/s-0030-1259724)
23. Du Y, Chang J, Reiner J, Zhao K. 2008 Formation of *N*-alkoxyindole framework: intramolecular heterocyclization of 3-alkoxyimino-2-arylalkylnitriles mediated by ferric chloride. *J. Org. Chem*. **73**, 2007–2010. (doi: 10.1021/jo7024477)
24. Benington F, Morin RD, Clark LC Jr. 1965 Behavioral and neuropharmacological actions of *N*-aralkylhydroxylamines and their *O*-methyl ethers. *J. Med. Chem*. **8**, 100–104. (doi: 10.1021/jm00325a020)
25. Nandurkar NS, Zhang J, Ye Q, Ponomareva LV, She Q-B, Thorson JS. 2014 The identification of perillyl alcohol glycosides with improved antiproliferative activity. *J. Med. Chem*. **57** 7478–7484. (doi: 10.1021/jm500870u)
26. Kang T, Kim Y, Lee D, Wang Z, Chang S. 2014 Iridium-catalyzed intermolecular amidation of sp^3 C–H bonds: Late-stage functionalization of an unactivated methyl group. *J. Am. Chem. Soc.* **136**, 4141–4144. (doi: 10.1021/ja501014b)
